# Multivalent antiviral conjugates enhance activity against Influenza via cross-linking viruses

**DOI:** 10.1038/s41467-026-78126-3

**Published:** 2026-10-04

**Authors:** Xun Feng, Lunzhi Yuan, Lei Zhao, Lianqi Liu, Fei Xie, Ming Zhou, Gong Cheng, Yi Guan, Ningshao Xia, Tong Cheng, Wu Zhong, Dian Xiao, Xinbo Zhou, Song Li

**Affiliations:** 1 Academy of Military Medical Sciences, Beijing, 100850 China; 2 National Engineering Research Center for the Emergency Drug, Beijing, 100039 China; 3 School of Basic Medical Sciences, Tsinghua University, Beijing, 100084 China; 4 State Key Laboratory of Molecular Vaccinology and Molecular Diagnostics, National Institute of Diagnostics and Vaccine Development in Infectious Diseases, School of Life Sciences, School of Public Health, Xiamen University, Xiamen, 361102 China; 5 State Key Laboratory of Emerging Infectious Diseases, School of Public Health, Li Ka Shing Faculty of Medicine, The University of Hong Kong, Hong Kong, Hong Kong SAR China; 6 Guangdong-Hong Kong Joint Laboratory of Emerging Infectious Diseases, Joint Institute of Virology (STU/HKU), Shantou University, Shantou, Guangdong China; 7 Shanghai Institute of Virology, Shanghai-Jiao-Tong University School of Medicine, Shanghai, China

**Keywords:** Drug development, Antivirals, Influenza virus

## Abstract

Long-acting drug–Fc conjugate (CD388) has emerged as a new class of antivirals against influenza A and B, yet the mechanisms governing how Fc conjugation reshapes small-molecule function remain undefined. Here we establish a programmable multivalent antiviral conjugates (AVCs) platform by click chemistry that enables flexible combination of valencies and conjugated drugs, allowing systematic interrogation of valency-dependent antiviral mechanisms. Using zanamivir as a model, mono-, di-, and trivalent zanamivir are conjugated with Fc, with trivalent zanamivir-Fc conjugates (AVC-Z3) exhibiting higher activity than lower-valent AVCs. AVC-Z3 induces inter-virion aggregation, thereby coupling blockade of viral entry with restriction of viral release. This cross-linking–mediated dual-stage inhibition represents a mechanistic shift from neuraminidase suppression to physical viral immobilization. Furthermore, trivalent peramivir-Fc conjugate (AVC-P3) is synthesised and exhibits stronger antiviral activity than AVC-Z3 in vitro, with much longer half-life in vivo than AVC-Z3, and CD388 with YTE mutation. The in vivo antivirus efficacy of AVC-P3 surpasses other AVCs, baloxavir, and CD388 in H1N1-, H3N2-, H5N1-, and influenza B-infection mice models. Together, our findings define ligand valency as a determinant of antiviral mechanism.

## Abstract (web summary)

A multivalent antiviral conjugates (AVCs) platform was established by conjugating NA inhibitors and Fc domain. AVCs cross-link flu viruses, blocking entry and release. Trivalent peramivir-Fc conjugate outperformed clinical drugs in mice.

## Introduction

Influenza viruses remain a persistent global health threat^1^, with seasonal epidemics and zoonotic strains (H5N1 et al.) posing continuous pandemic risks^2–4^. Current antiviral strategies primarily target viral enzymatic functions, most notably neuraminidase (NA), which facilitates viral release and spread^5,6^. Licensed neuraminidase inhibitors (NAIs), including zanamivir^7^, oseltamivir^8^, and peramivir^9^, act by competitively blocking the catalytic site of NA, thereby reducing viral propagation. However, these inhibitors operate through a purely enzymatic mechanism and do not alter the spatial organisation or physical behaviour of virions. Consequently, antiviral efficacy remains constrained to catalytic inhibition without engaging higher-order structural interference.

Multivalency is a fundamental principle in biological recognition, enabling enhanced avidity and emergent properties beyond monovalent binding^10,11^. In antiviral biology, multivalent antibodies and engineered nanostructures have demonstrated superior neutralisation potency, partly through cross-linking viral particles^12–17^. Small molecule-macromolecule conjugate antiviral drugs has been also previously reported. Hidde L. Ploegh group and Jianzhu Chen group have developed zanamivir conjugates for influenza protection^18,19^, focusing on antibody modification with a single antiviral moiety. Philip S. Low group and Hidde L. Ploegh group have reported a dual-mechanism immunotherapy for influenza, recruiting or activating immune effectors^20,21^. The drug-Fc conjugate (DFC) CD388, developed by Cidara Therapeutics, is a first-in-class antiviral drug for influenza prevention and treatment^22–24^. CD388 comprises a dimeric zanamivir linked to a CH1–Fc hybrid domain of human IgG1 with a drug-Fc ratio of approximately 4.5, exhibiting improved activity and extended half-life compared to zanamivir^23,24^. Phase 2b NAVIGATE Trial showed CD388 achieved all endpoints, with 450 mg providing 76.1% protection^25^. In comparison, seasonal influenza vaccines provide only 40% protection due to mutations, with 10–30% against H3N2 subtypes^26–28^. Nevertheless, whether multivalency in DFC merely increases binding strength or fundamentally reshapes the antiviral mode of action remains unclear.

Structural analyses of influenza NA tetramers reveal spatial arrangements that could theoretically permit ligand engagement across adjacent NA complexes or even between separate virions, depending on inter-ligand spacing. Two-dimensional representation of the exposed top surface of influenza virus neuraminidase (NA) tetramers shows three binding modes: intradermic binding (50 Å)^29^, dimer binding to neighbouring NA tetramers (25–30 Å)^30^, and dimer binding to NA tetramers on different virions (16 Å)^30^. Such geometry raises the possibility that appropriately designed multivalent NA inhibitors might mediate inter-virion cross-linking.

**Fig. 1:**
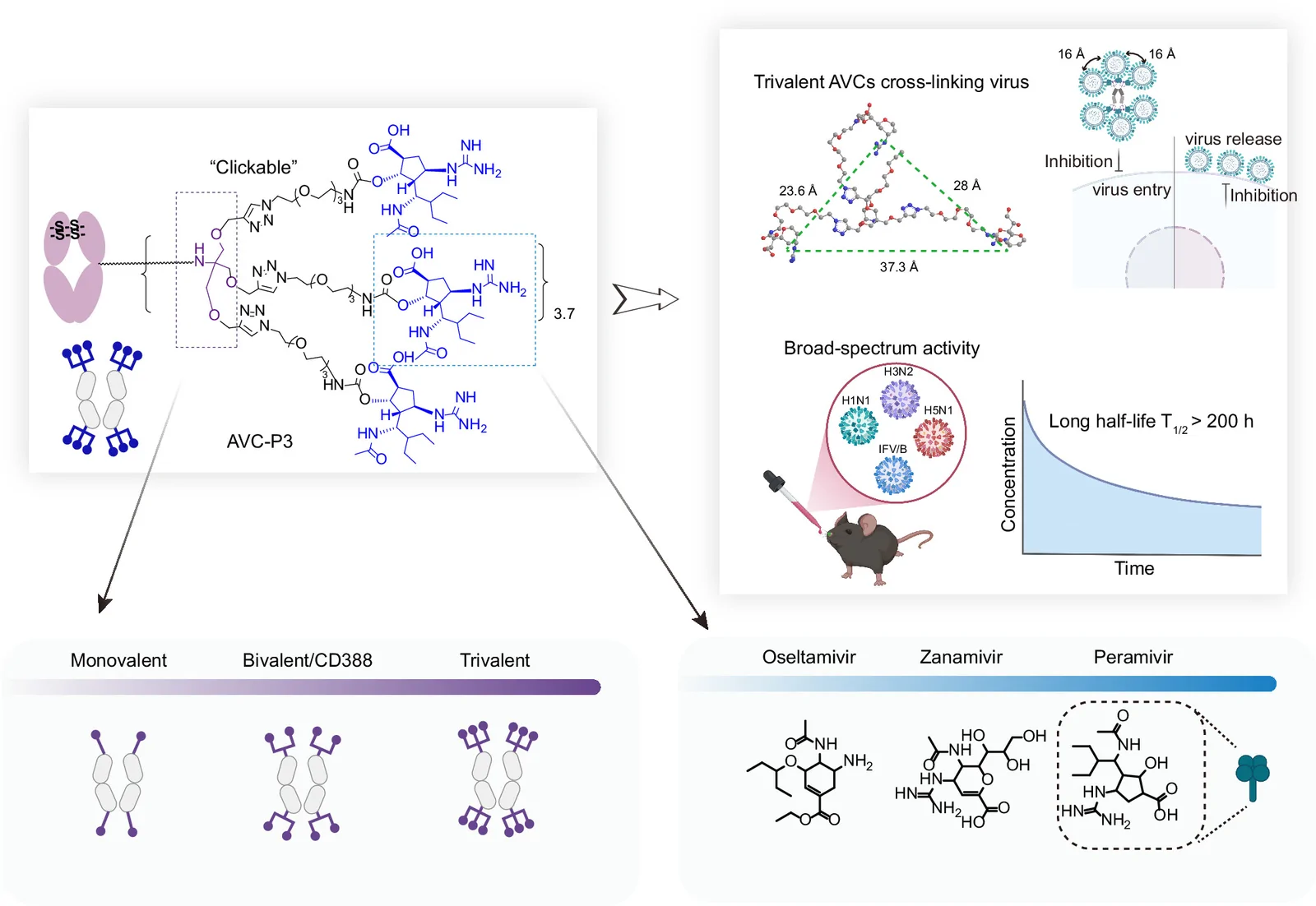
The design of multivalent AVCs platform by click chemistry. Mono-, di-, and trivalent zanamivir were conjugated with Fc to form AVC-Z1, AVC-Z2, AVC-Z1H, and AVC-Z3. Trivalent oseltamivir and peramivir were conjugated to Fc to form AVC-O3 and AVC-P3. The distance between the trivalent NA inhibitors in the AVCs ranged from 23.6 Å to 37.3 Å, ensuring that different viruses (16 Å) can achieve cross-linking. The preferred AVC-P3 displayed a broad-spectrum anti-influenza activity and long half-life in vivo. Created in BioRender. Xiao, D. (2026) https://BioRender.com/dp6lrp9.

In this study, we present the construction of a programmable multivalent drug design platform (Fig. 1, Supplementary Fig. 10) by click chemistry. First, we designed and synthesised mono-, di-, and trivalent zanamivir to conjugate with Fc with a DFR of 4.0, generating tetra- (4), octa- (8), and dodecavalent (12) DFCs (named multivalent antiviral conjugate; AVC). Additionally, listed neuraminidase inhibitors (NAIs), including zanamivir, oseltamivir, and peramivir, were selected for conjugation to the Fc domain for comparison. The linker distance between the multivalent NA inhibitors ranges from 23.6 Å to 37.3 Å, enabling cross-linking virions^30^. We demonstrate that increasing valency induces a mechanistic switch from conventional catalytic inhibition to a supramolecular antiviral mode of action characterised by inter-virion cross-linking and viral aggregation. High-valence constructs reorganise influenza virions into clustered assemblies that restrict both entry into host cells and subsequent viral release. Among the constructs tested, the lead candidate AVC-P3 exhibited the strongest aggregation capacity, prolonged systemic persistence, and superior in vivo protective efficacy compared to lower-valence AVCs and CD388. These findings establish that ligand valency and spatial geometry can reprogram antiviral mechanisms at the virion level. More broadly, owing to the applied click chemistry, this multivalent antiviral conjugates platform is highly programmable, beneficial to the development of drugs for epidemic diseases in the future.

## Results

### Construction of a clickable multivalent AVCs platform

**Fig. 2:**
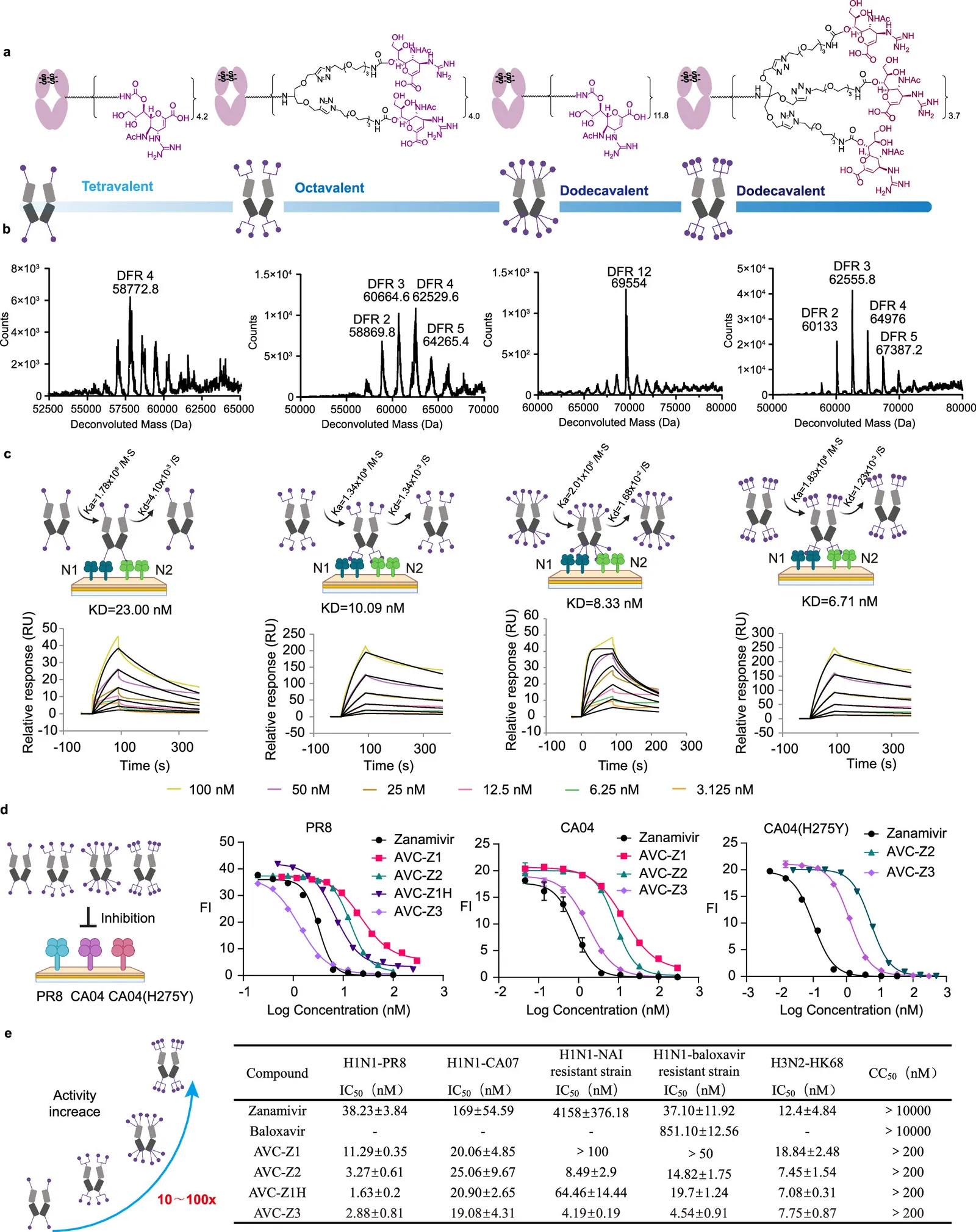
Characterisation, SPR analysis and NA inhibition of AVCs. **a** Conjugating monovalent, divalent, and trivalent zanamivir to Fc to form AVC-Z1, AVC-Z2, AVC-ZH and AVC-Z3. **b** UPLC-Q-TOF-MS analysis of AVC-Z1, AVC-Z2, AVC-Z1H, and AVC-Z3 revealing the DFRs were 4.2, 4.0, 11.8 and 3.7. **c** SPR analysis revealing that AVC-Z1, AVC-Z2, AVC-Z1H, and AVC-Z3 exhibiting progressively higher affinity for N1 and N2 subtypes. RU, response units. **d** NA inhibition analysis revealing that AVC-Z1, AVC-Z2, AVC-Z1H, AVC-Z3 exhibit progressively higher inhibition activity for NA. **e** IC_50_ values [nM] of AVCs, zanamivir, or baloxavir against a panel of influenza strains A/H1N1, A/H3N2, B/Forida/78, and drug-resistant strains. SPR results (**c**), data (**d**) and IC_50_ values (**e**) are representative of three independent experiments. Data are shown as mean ± s.d. Source data are provided as a Source Data file. Created in BioRender. Xiao, D. (2026) https://BioRender.com/dp6lrp9.

To investigate valence impact on antiviral activity, we leveraged “click chemistry” to construct a multivalent AVCs platform. Mono-, di-, and tri- zanamivir were conjugated with Fc to develop AVC-Z1, AVC-Z2, AVC-Z1H, and AVC-Z3, respectively (Fig. 2a, Supplementary Figs. 1–3 and 6, Supplementary methods 1-2, Supplementary Figs. 8, 9). UPLC-Q-TOF-MS analyses showed average DFRs of 4.2, 4.0, 11.8, and 3.7, respectively, indicating AVC-Z1, AVC-Z2, AVC-Z1H, and AVC-Z3 contained approximately 4.2, 8.0, 11.8, and 11.1 zanamivir, respectively (Fig. 2b). Size-exclusion chromatography (SEC) confirmed that the aggregation of all AVCs was below 10% (Supplementary Fig. 11).

Multivalence creates avidity which may improve antivirus activity^12–17^. Surface plasmon resonance (SPR) analysis revealed that AVC-Z1, AVC-Z2, AVC-Z1H, and AVC-Z3 exhibited progressively higher affinities for NA (N1 and N2 subtypes) (Fig. 2c, Supplementary Fig. 12), with equilibrium dissociation constants (KD) = 23.00 nM, 10.09 nM, 8.33 nM, and 6.71 nM (N2), respectively. All AVCs showed greater affinity than zanamivir monomers (Supplementary Fig. 12) and displayed significantly slower dissociation from NA, particularly N2. Among all AVCs, AVC-Z3 exhibited the strongest binding affinity for NA, with KD = 10.10 nM (N1) and KD = 6.71 nM (N2), and the slowest dissociation rates, KD = 1.44 × 10⁻² /s (N1) and KD = 1.23 × 10⁻³ /s (N2) (Fig. 2c), suggesting extended NA binding and prolonged inhibition.

The inhibition assays against NA protein (A/Puerto Rico/8/1934 and A/California/04/2009) and oseltamivir-resistant NA subtype (H275Y mutation) revealed the NA inhibitory activity of AVCs improved with increased valence. The inhibitory activity of AVC-Z3 against PR8 NA was 17, 10, and 5 times higher than that of AVC-Z1, AVC-Z2, and AVC-Z1H, respectively (Fig. 2d). No decrease in the AVC-Z3 inhibitory activity was observed in the oseltamivir-resistant NA subtype (H275Y mutation). Zanamivir was also highly potent against NA, demonstrating nM of half-maximal inhibition concentrations (IC_50_) against H1N1 strains. AVC-Z3 displayed less than 2 nM of IC_50_ across all three NA subtypes, demonstrating strong antiviral potential (Fig. 2d).

To evaluate the antiviral activity, we tested AVCs against five influenza virus strains in MDCK cells (Fig. 2e). AVCs exhibit superior antiviral activity compared to zanamivir and peramivir. Moreover, the antivirus activity increased with valence numbers, showing potency: AVC-Z3 > AVC-Z2 > AVC-Z1H > AVC-Z1 > zanamivir (Fig. 2e). In addition, we have designed a short-linker monovalent zanamivir conjugate (AVC-Z1-H, Supplementary Fig. 7) to investigate the impact of linkers. AVC-Z1H-S possesses a similar DFR value with AVC-Z1H, but its antiviral activity is significantly reduced, with median IC_50_ values of 31.79 nM against CA07 and 12.84 nM against PR8 (Supplementary Table 1). Among them, AVC-Z3 showed the most potent antiviral activity, with median IC_50_ values of 10.98 nM against subtype A/H1N1, 7.75 nM against subtype A/H3N2. The median IC_50_ values for AVC-Z3 were 1–2 logs lower than those for AVC-Z1 and AVC-Z2 against the same strains. AVC-Z3 retained high antiviral activity (~4 nM) against NAI- (A/Liaoning-Zhenxing/1109/2010) and baloxavir-resistant strains (A/Puerto Rico/8/1934, I38T). These findings indicated that higher valences increased the affinity, NA inhibition, and antivirus activity, and AVC-Z3 was selected for further research.

### AVC-Z3 cross-linking virus to inhibit virus invasion and release

**Fig. 3:**
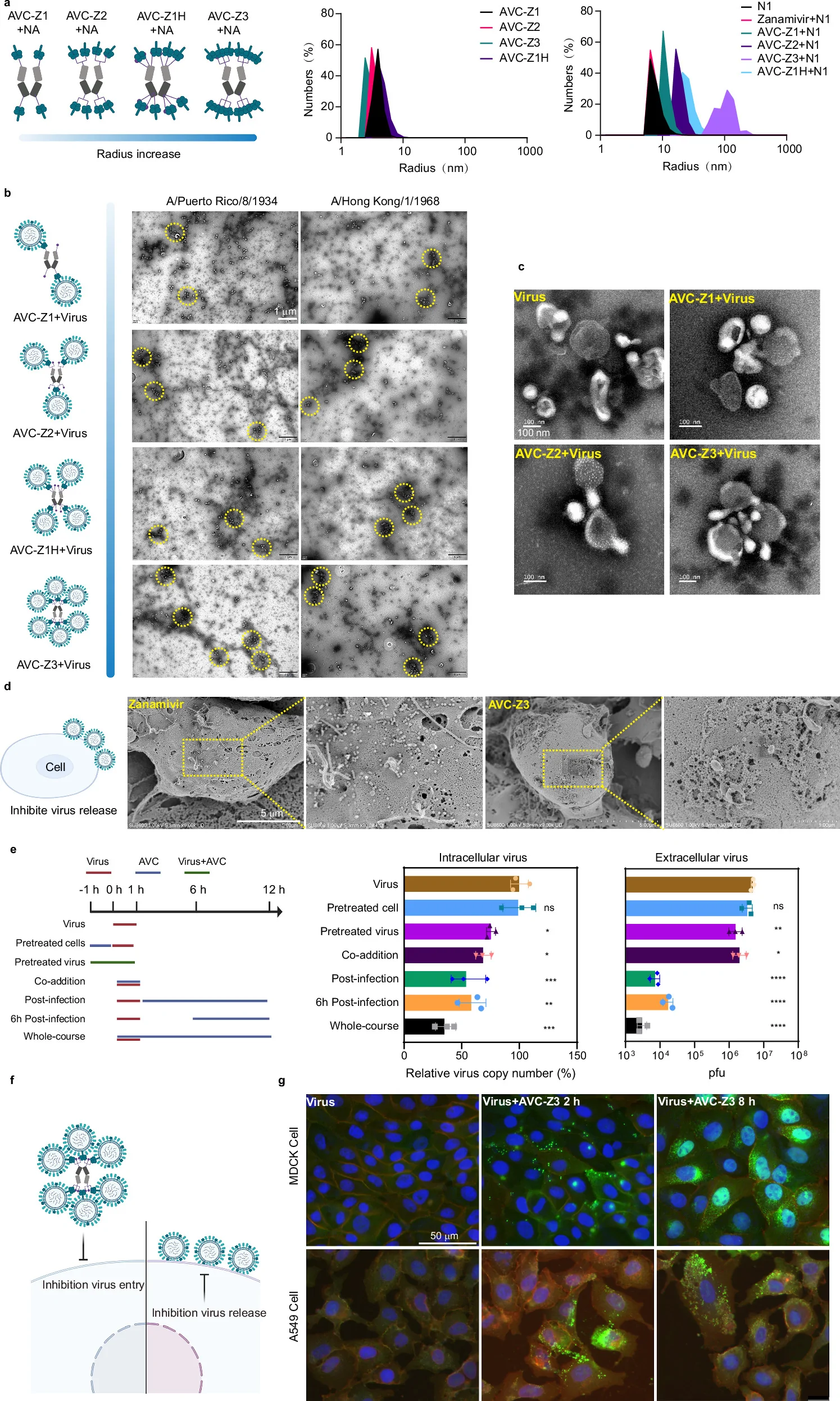
AVCs exerted antiviral effects through cross-linking virus. **a** Particle size distribution patterns of N1, zanamivir + N1, AVCs and AVCs + N1. **b** TEM micrographs of zanamivir + virus, AVC-Z3 + virus, and AVC-P3 + virus. Scale bar, 1 μm. **c** TEM micrographs of virus, Fc + virus, AVC-Z1 + virus, AVC-Z2 + virus, AVC-Z3 + virus and AVC-P3 + virus, Scale bar, 100 nm. **d** SEM analysis revealed that while both AVC-Z3 and AVC-P3 treated cells retained and induced virus aggregation on their surfaces. Scale bar, 5 μm. **e** The strategy of the time-of-drug-addition assay revealed that AVC-Z3 exerted antiviral effects by inhibiting virus invasion and virus release. *P* values (vs. Virus group): Intracellular virus assay (*n* = 3) — pretreated cell, *P* > 0.9999 (ns); pretreated virus, *P* = 0.0495 (*); co-addition, *P* = 0.0113 (*); post-infection, *P* = 0.0006 (***); 6 h post-infection, *P* = 0.0013 (**); whole-course, *P* < 0.0001 (****). Extracellular virus assay (*n* = 3) — pretreated cell, *P* = 0.6178 (ns); pretreated virus, *P* = 0.0052 (**); co-addition, *P* = 0.0308 (*); post-infection, 6 h post-infection, whole-course, all *P* < 0.0001 (****). **f** AVCs could both inhibit virus invasion and release. **g** Fluorescence imaging of AVC-P3 treatment at the indicated time (2 h and 8 h post infection) against the PR/8-NS1-GFP reporter virus, indicating that AVC-P3 induces virus aggregation in the early and late stages of infection. Images (**b**–**d**, **g**) and data (**a**) are representative of three independent experiments. Intracellular and extracellular data (**e**) are representative of two and three independent experiments. Data are shown as mean ± s.d. *P* values were calculated using one-way ANOVA with Dunnett’s multiple comparisons test. NS, not significant. Source data are provided as a Source Data file. Created in BioRender. Xiao, D. (2026) https://BioRender.com/dp6lrp9.

Then we investigated the mechanism of interaction between AVCs and the virus. To explore AVC and NA protein binding, we performed dynamic light scattering analysis to determine AVC–NA complex size. The molecular weight of NA is 55.9 kDa, with a particle diameter of 6 nm, whereas that of AVCs ranges from 1 to 10 nm in diameter (Fig. 3a). The particle size of the AVC–NA complexes increased, correlated positively with AVC valence (Fig. 3a). These results suggested that multivalent compounds can simultaneously bind to multiple NA proteins, leading to their aggregation. However, AVC-Z3 produced significantly larger NA aggregates (~100 nm) than AVC-Z2 and AVC-Z1H (~20 nm), despite AVC-Z1H containing an equivalent (~12) zanamivir monomers and exhibiting comparable antiviral activities. Transmission electron microscopy (TEM) confirmed that AVC-Z3 induced more extensive cross-linking of NA proteins, forming larger aggregates than the other AVCs (Fig. 3a).

To explore AVCs interactions with viral particles, we incubated AVCs with H1N1 (A/Puerto Rico/8/1934) or H3N2 (A/Hong Kong/1/1968) viruses. TEM (Fig. 3b) revealed that AVC induced viral aggregation in a valency-dependent manner (AVC-Z3 > AVC-Z2 > AVC-Z1). Despite identical conjugated zanamivir molecules, AVC-Z3 induced significantly larger and more numerous viral aggregates than AVC-Z1H. Higher magnification shows sparsely distributed when treated with Fc. AVC-Z1 and AVC-Z2 can simultaneously capture multiple viruses, reducing inter-viral distance. With AVC-Z3 treatment, the viruses significantly aggregated and bound tightly (Fig. 3c).

Given that AVC-Z3 demonstrated optimal antiviral activity and superior virus cross-linking capability, we investigated its interaction pattern using an in vitro infection model. H1N1 virus was incubated with MDCK cells for 1 h, followed by extracellular virus removal and treatment with zanamivir (150 μM) or AVC-Z3 (12.5 μM) for 10 h. After washing free viral particles, scanning electron microscopy (SEM) revealed that zanamivir and AVC-Z3-treated cells retained viral particles on their surfaces (Fig. 3d, Supplementary Fig. 13). Zanamivir treatment partially restricted viral release, with sparse individual viral particles on cell surfaces. In contrast, AVC-Z3 treatment markedly inhibited viral release, leading to substantial viral aggregation on the cell surface through mutual adhesion (Fig. 3d). These findings indicate that in the in vitro infection model, the ability of AVC-Z3 to simultaneously engage multiple viral NA proteins facilitates extensive viral aggregation on cell surfaces, restricting viral particle release and preventing viral dissemination.

NAIs restrict progeny virus release from infected cells. However, because AVC-Z3 promotes extracellular viral aggregation, we hypothesised that it may interfere with viral adsorption and entry by trapping free viral particles. Therefore, MDCK cells were treated with AVC-Z3 at different time points prior to or post-infection (Fig. 3e). Twelve hours post-infection (hpi), we assessed intracellular viral replication using qPCR (Fig. 3e). Results showed that AVC-Z3 significantly reduced viral replication in the pretreated virus group (*P* = 0.0495, *) and co-addition group (*P* = 0.0113, *), suggesting that AVC-Z3 inhibited viral adsorption and entry, reducing infection and intracellular viral replication. To evaluate the extracellularly released virus, we performed plaque assays on the supernatants from the different treatment groups (Fig. 3e). To exclude the possibility that the inhibition is simply due to the bulky Fc-conjugate sterically shielding adjacent hemagglutinin (HA) binding sites, we conducted the entry blockade assays using the short-linker AVC-Z1H-S. The results showed that AVC-Z1H-S also containing the bulky Fc groups did not block the viral entry process. The inter-viral cross-linking might need a sufficiently long linker (Supplementary Fig. 14). AVC-Z3 treatment significantly reduced extracellular viral titres, regardless of whether it was administered at the time of infection or post-infection (*P* < 0.0001, ****). In the pretreated virus (*P* = 0.0052, **) and co-addition groups (*P* = 0.0308, *), extracellular viral titres were reduced, supporting that AVC-Z3 inhibits viral adsorption and entry, besides blocking progeny virus release (Fig. 3f). To validate the dual inhibition mechanism, cells were infected with the PR/8 reporter virus (PR8-GFP) and observed using laser confocal microscopy. AVC-Z3 was added at 2 and 8 h post PR8-GFP infection. Results revealed that AVC-Z3 could cross-link the free virus and cell-surface virus, with significant aggregation of green fluorescence on MDCK and A549 cell (Institute of Basic Medicine, Chinese Academy of Medical Sciences, Beijing, China) surfaces (Fig. 3g). We identified that the AVC-Z3 exerts antiviral effects through virus cross-linking, featuring a novel dual inhibition mechanism of virus invasion and release.

### Design and characterisation of AVC-O3 and AVC-P3

**Fig. 4:**
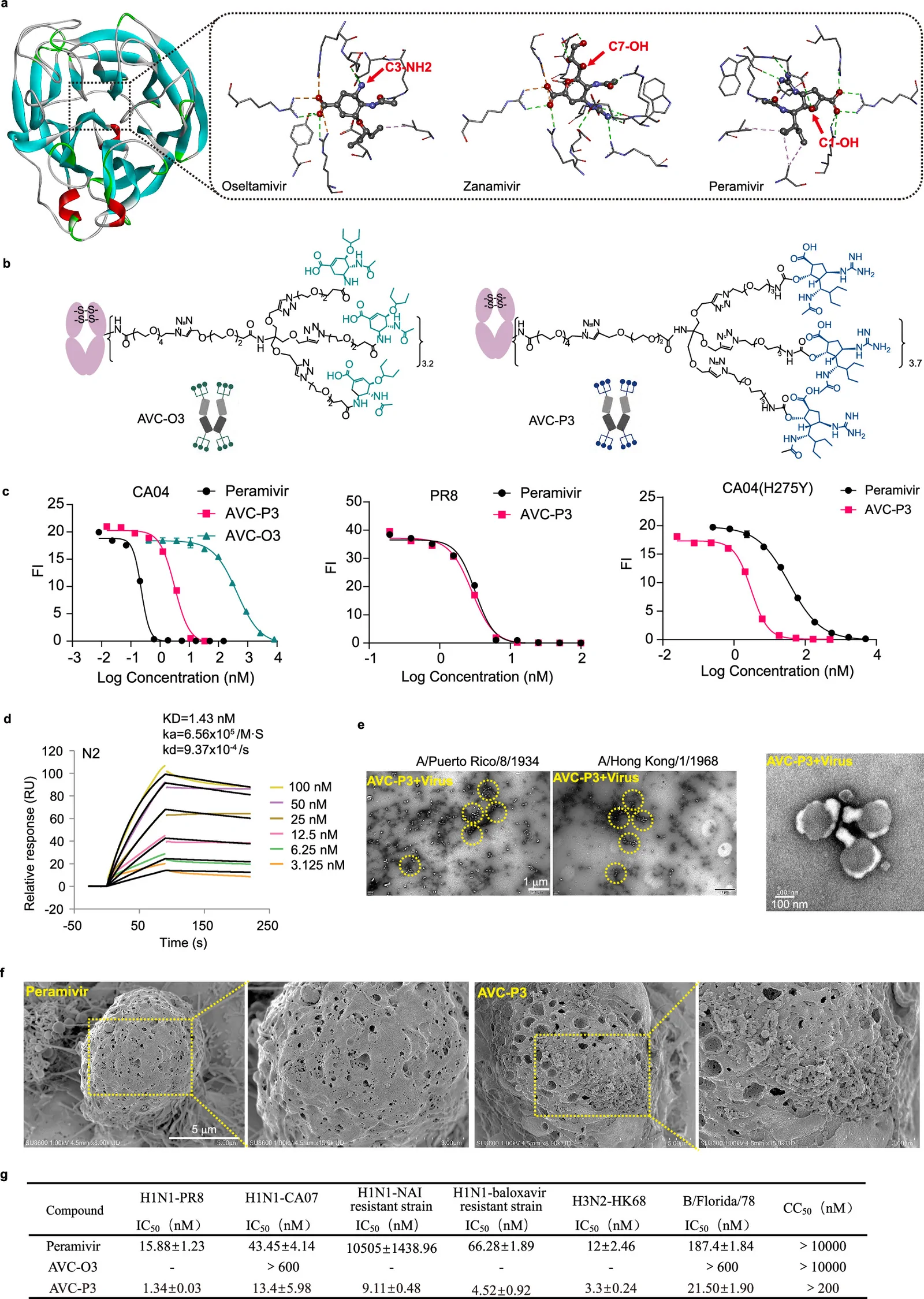
Design and characterisation of AVC-O3 and AVC-P3. **a** The derivatization site analysis for zanamivir, oseltamivir, and peramivir. **b** The simplified structure of AVC-O3 and AVC-P3. **c** NA inhibition analysis reveals that AVC-P3 exhibits high NAI activity. **d** SPR analysis revealing that AVC-P3 exhibits a high affinity for N2 subtypes. **e** TEM micrographs of AVC-P3 + virus. Scale bar, 1 μm. **f** SEM analysis revealed that while both AVC-Z3 and AVC-P3 treated cells retained and induced virus aggregation on their surfaces. Scale bar, 5 μm. **g** IC_50_ and CC_50_ values [nM] of AVCs or peramivir against a panel of influenza strains A/H1N1, A/H3N2, B/Forida/78, and drug-resistant strains. Data (**c**), SPR results (**d**) and Images (**e,** **f**) are representative of three independent experiments. Data are shown as mean ± s.d. Source data are provided as a Source Data file. Created in BioRender. Xiao, D. (2026) https://BioRender.com/dp6lrp9.

Next, we explored the influence of different NAI conjugated with Fc on antivirus activity. First, the linking site of NAI was studied to avoid affecting the NAI activity. Although the amino group at the C5 position of oseltamivir forms hydrogen bonds with Glu119 and Asp151 in the NA active site, it was selected for derivatisation due to its ability to accommodate larger substituents (Fig. 4a, Supplementary Fig. 5, Supplementary methods 1-2, Supplementary Fig. 8, 9). The hydroxyl group at the C7 position of zanamivir faces the outside of the pocket; therefore, it was selected as the derivatisation site. For peramivir, the hydroxyl group at the C2 position, which has fewer interactions and faces the outside of the cavity, was selected as the derivatisation site (Fig. 4a, Supplementary Fig. 4, Supplementary methods 1-2, Supplementary Figs. 8, 2). Therefore, trivalent oseltamivir and peramivir were used to synthesise the forms conjugated with Fc to obtain AVC-O3 and AVC-P3 (Fig. 4b). SEC confirmed that the aggregation of all AVCs was below 5% (Supplementary Fig. 15a, b). UPLC-Q-TOF-MS analyses showed that the DFR of AVC-O3 and AVC-P3 were 3.2 and 3.7, respectively (Supplementary Fig. 15c, d).

AVC-O3 showed weak NA inhibitory activity of IC₅₀ = 442.10 nM, and its cellular antiviral EC₅₀ values were above 600 nM (Fig. 4c, Supplementary table 1). This was attributed to the long PEG chain from C5 position causing significant steric hindrance and impeding oseltamivir entry into the active-site pocket. Therefore, the AVC-O3 design was excluded from this study. In contrast, AVC-P3 maintained potent and equivalent inhibitory activity against wild-type and H275Y mutant NA, with IC₅₀ value 3 nM, significantly better than peramivir (Fig. 4c). For H275Y mutant NA, peramivir showed >10-fold decrease in inhibitory activity, whereas AVC-P3 retained high antiviral activity (Fig. 4c). AVC-P3 demonstrated high affinity for NA protein, with KD of 1.43 nM and 3.29 nM against NA from A/Darwin/6/2021 and A/Sydney/5/2021, respectively (Fig. 4d). AVC-P3 exhibited slow dissociation kinetics (KD = 9.37 × 10⁻⁴ /s and KD = 8.26 × 10⁻³ /s). AVC-P3 also induced cell-free virus and cell-surface virus aggregation (Fig. 4e, f). Similar enhancements in antiviral activity for AVC-P3, with median IC_50_ values of 7.37 nM and 3.3 nM against subtype A/H1N1 and A/H3N2, respectively (Fig. 4g). AVC-P3 retained nanomolar antiviral activity against NAI- and baloxavir-resistant strains, exhibiting 188- to 1167-fold increase in activity compared to peramivir and baloxavir. AVC-P3 had a half-maximal cytotoxic concentration (CC_50_) of >200 nM, resulting in a > 1,00-fold selectivity index for AVCs (Fig. 4g). These data demonstrate AVC-P3 exhibiting high antivirus activity equivalent to AVC-Z3, with a similar cross-linking virus mechanism.

### Superior pharmacokinetic properties of AVC-P3

**Fig. 5:**
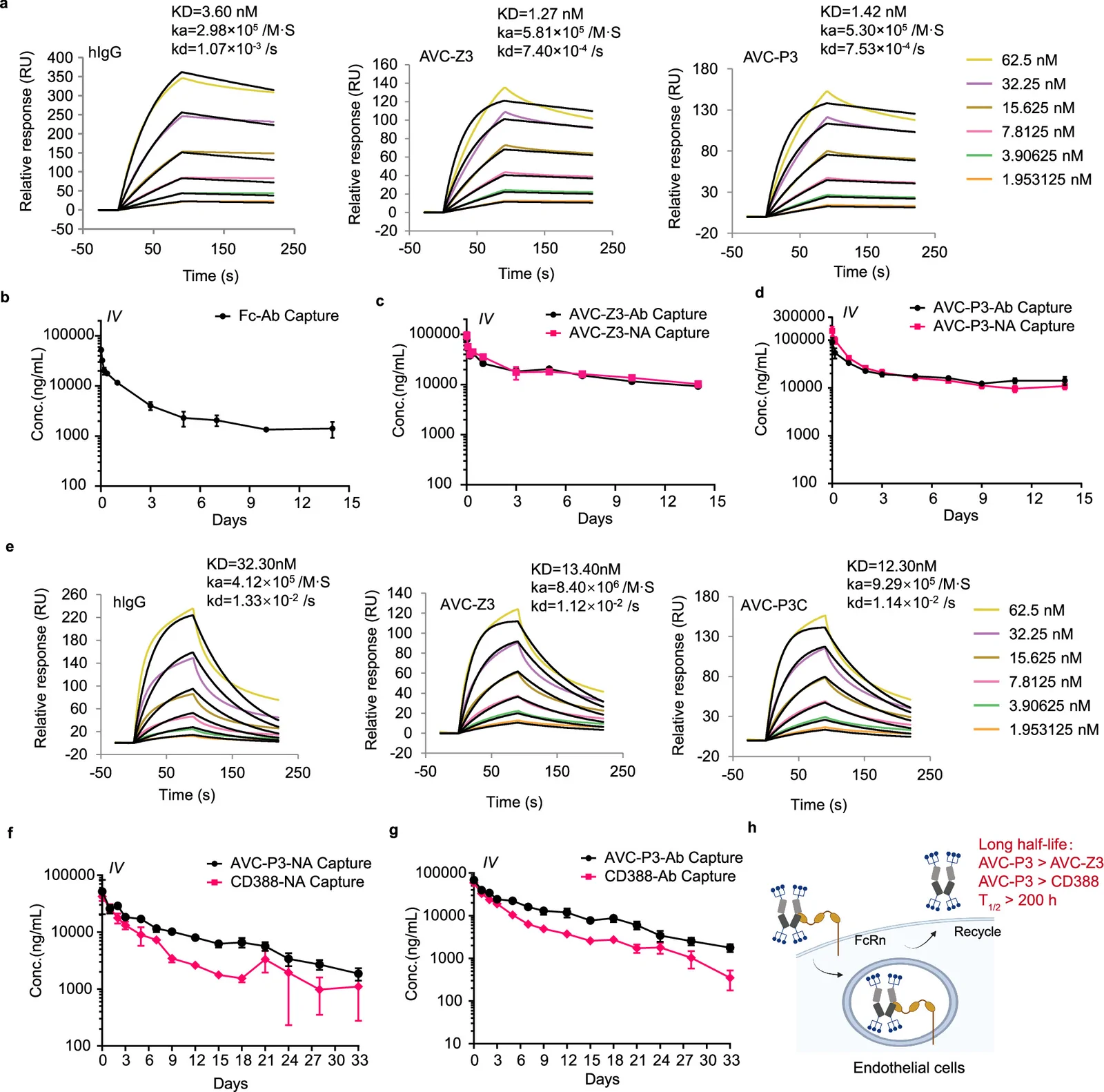
The in vivo pharmacokinetic properties of AVC-Z3 and AVC-P3. **a** The affinity analysis of hIgG, AVC-Z3, and AVC-P3 for the mouse FcRn receptor by SPR. **b** Detection of Fc (*n* = 3 mice per group) protein concentrations in plasma using an anti-human IgG-Fc antibody for capture. **c, d** Detection of AVC-Z3 (*n* = 3 mice per group, C57BL6J mice, **c**) and AVC-P3 (*n* = 4 mice per group, C57BL6J mice, **d**) protein concentrations in plasma using an anti-human IgG-Fc antibody and NA for capture. **e** The affinity analysis of hIgG, AVC-Z3 and AVC-P3 for the human FcRn receptor by SPR. **f, g** Comparing the half-life of AVC-P3 (*n* = 6 mice per group, FcRn knock-in mice, **f**) and CD 388 (*n* = 6 mice per group, FcRn knock-in mice, **g**) using an anti-human IgG-Fc antibody and NA for capture. **h** AVC-P3 displaying a longer half-life than AVC-Z3 and CD388. SPR results (**a, e**), data (**b–d, f, g**) are representative of three independent experiments. Data are shown as mean ± s.d. Source data are provided as a Source Data file. Created in BioRender. Xiao, D. (2026) https://BioRender.com/dp6lrp9.

Although the trimeric NAI conjugate strategy demonstrated potential to boost antiviral activity, we assessed AVC affinity for the FcRn receptor using SPR. Antibodies in the blood are endocytosed by vascular endothelial cells into endosomes. The acidic pH within endosomes (pH 5.0–6.0) promotes binding between the Fc domain and FcRn. Subsequently, the antibody–FcRn complex is recycled to the cell surface via membrane trafficking. At physiological plasma pH (7.4), the antibody dissociates from FcRn and returns to circulation. Owing to FcRn cross-species reactivity, human antibodies can bind to human and murine FcRn receptors. SPR analysis at pH 5.0 revealed that AVC-Z3 (KD = 1.27 nM) and AVC-P3 (KD = 1.42 nM) (Fig. 5a) bound tightly to murine FcRn, with slow dissociation rates comparable to those of human IgG antibody (KD = 3.60 nM, Fig. 5a).

To evaluate the in vivo half-life, we intravenously injected AVC-Z3 or Fc protein into 6–8-week-old female C57Bl/6 J mice. Blood samples were collected at various timepoints. AVC and Fc protein concentrations in the plasma were quantified using an anti-human IgG-Fc antibody for capture. Analysis using a biphasic model showed that AVC-Z3 had a half-life (T₁/₂) of 141.2 h (Fig. 5c, Extended Fig. 6b), exceeding that of the Fc protein (T₁/₂ = 34.3 h, Fig. 5b) and zanamivir (T₁/₂ = 2.5–5.05 h)^31^. Similar results were obtained when the NA protein was used as the capture agent for serum AVC-Z3 quantification, further confirming the in vivo stability and improving the small-molecule plasma half-life. Results showed that the half-life of AVC-P3 (T₁/₂ = 225.02 h; Fig. 5d) exceeded that of AVC-Z3, due to their different isoelectric points.

**Fig. 6:**
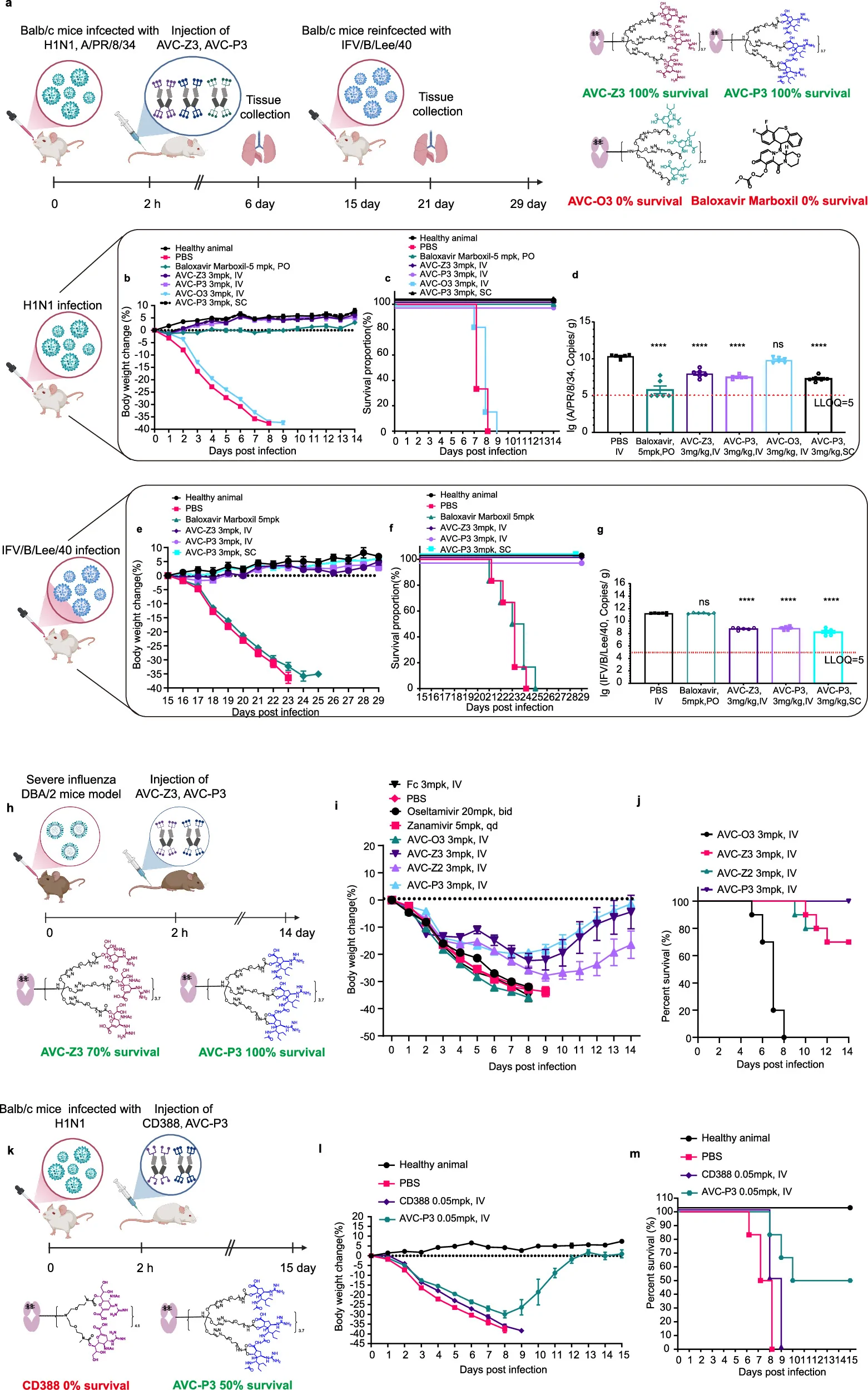
AVC-P3 demonstrated superior effects in vivo than other AVCs and CD388. **a** Experiment design of AVCs and baloxavir in lethal mouse models of reinfected with influenza B. **b**–**d** %body weight change (**b**), survival curve (**c**) and lung viral burden (**d**) of AVCs (*P* < 0.0001; AVC-O3 vs PBS: *P* = 0.4446) and baloxavir (*P* < 0.0001) in the first influenza A infection model (*n*  =  12 mice per group except *n* = 6 mice in PBS group). **e**–**g** %body weight change (**e**), survival curve (**f**) and lung viral burden (**g**) of AVCs (*P* < 0.0001) and baloxavir (*P* = 0.9995) in the influenza B reinfection model (*n*  =  6 mice per group). **h** Experime*n*tal design of AVCs, oseltamivir, and zanamivir on a severe influenza model using DBA/2 mice. **i, j** %body weight change (**i**) and survival curve (**j**) of AVCs **i**n the H1N1 infection DBA/2 mice (*n*  =  10 mice per group). **k** Experiment design of AVC-P3 and CD388 in lethal mouse models of infected H1N1. **l, m** %body weight change (**l**), survival curve (**m**) of AVC-P3 and CD388 in the H1N1 infection model (*n*  =  6 mice per group). Data are shown as mean ± s.d. *P* values were calculated using one-way ANOVA with Dunnett’s multiple comparisons test. NS not significant. Source data are provided as a Source Data file. LLOQ: Lower Limit of Quantification. Created in BioRender. Xiao, D. (2026) https://BioRender.com/dp6lrp9.

Considering the plasma half-life of AVC-P3 in wild-type mice, we used humanised FcRn mice to evaluate its pharmacokinetics properties. AVC-P3 showed strong affinity for human FcRn (KD = 12.3 nM), comparable to that of human IgG antibody (KD = 32.3 nM; Extended Fig. 6a). The plasma concentrations of AVC-P3 were measured using the same protocol (Fig. 5e). Detection using the anti-human IgG-Fc antibody revealed a long half-life of 253 h for AVC-P3 (Fig. 5f, g). Quantification using the NA protein as the capture agent yielded consistent results (Fig. 5f, g). CD388 with YTE mutation showed a shorter half-life of 163 h. These findings demonstrated the exceptional pharmacokinetic properties of AVC-P3 and its potential for long-lasting antiviral protection. The significantly longer half-life of AVC-P3 than AVC-Z3 and CD388 might indicate a better protective effect in vivo.

In addition to the advantage of the Fc protein in prolonging the half-life, we explored the antibody-dependent cellular cytotoxicity (ADCC) and antibody-dependent cellular phagocytosis (ADCP) effects conferred by the Fc protein of AVC-P3. Results showed that AVC-P3 exhibited concentration-dependent ADCC and ADCP with EC_50_ values of 7.3 ng/mL and 99.1 ng/mL, respectively, which was significantly stronger than that of CD388, with an EC_50_ values of 12.5 ng/mL and 136.1 ng/mL, respectively, possibly due to its stronger affinity for NA (Supplementary Fig. 16a, b). These immune effects may enhance its antiviral efficacy in vivo.

### Superior antiviral protection of AVC-P3 in vivo than other AVCs and CD388

Given the superior in vitro and in vivo antiviral activity and favourable pharmacokinetic properties of AVCs, we investigate their long-term effects against influenza A and B. Female BALB/c mice (6–8 weeks old, n = 12 per group) were infected with H1N1 (1 × 10⁶ TCID₅₀) and reinfected with IFV/B/Lee/40 on day 15 after the initial infection (Fig. 6a). The mice received AVCs and baloxavir at 2 hpi. On day 6 post-infection, half of the mice in each group were euthanised for lung viral load quantification. Body weight and survival of the remaining mice were monitored until day 14. Results showed that untreated (PBS) and AVC-O3 treated mice began dying on day 7 and were dead by day 9 (Fig. 6b, c). In contrast, AVC-Z3, AVC-P3 (3 mg/kg), and baloxavir (5 mg/kg) provided 100% protection until day 14 (Fig. 6b, c), with no significant body weight loss (Fig. 6b, c). AVC-P3 (3 mg/kg), AVC-Z3 (3 mg/kg) and baloxavir (5 mg/kg) all significantly reduce viral load in the lungs, with the best effect achieved in the baloxavir-treatment group (Fig. 6d). However, after the second infection on day 15, baloxavir-treated mice began dying on day 22, and died on day 25 (Fig. 6e, f). In contrast, AVC-Z3 and AVC-P3 (3 mg/kg) provided 100% protection until day 29 (Fig. 6e, f). Analysis of the lung viral load revealed that AVCs significantly suppressed viral replication compared to that in the untreated infected and baloxavir groups, reducing viral copies per gram of lung tissue by 100- to 1000-fold (Fig. 6g). These results indicated that AVCs have a long-lasting advantage over baloxavir.

To compare the therapeutic efficacies of different AVCs against severe influenza, we established a mouse model using highly susceptible DBA/2 mice^32^. Mice were intranasally infected with H1N1 virus (A/California/07/2009, 1 × 10⁴ PFU). Two hours after challenge, the mice received either a single dose of AVCs (3 mg/kg) or oseltamivir (20 mg/kg, bid for 5 days) (Fig. 6h). Similar to the PBS and Fc protein control groups, oseltamivir group exhibited a continuous weight loss. Mice treated with AVCs showed attenuated weight loss by day 3 and regained weight between days 7 and 8 (Fig. 6i). At 8–9 days post-infection (dpi) with the H1N1 virus, the maximum body weight loss in the AVC-Z2, AVC-Z3, and AVC-P3 groups was 22.34%, 26.70%, and 19.33%, respectively, indicating that the in vivo protective effect increased with AVC valence. Mortality in the oseltamivir group began on day 6 post-challenge and reached 100% by day 8. In contrast, a single dose of AVC-Z2 or AVC-Z3 achieved 70% protection, whereas AVC-P3 provided 100% protection (Fig. 6j). These results demonstrate that a single AVC dose remains effective in a severe infection model, suggesting potential clinical value for treating patients with severe influenza infection. The in vivo protective effect trend showed that AVC-P3 is the best, followed by AVC-Z3, then AVC-Z2.

AVC-P3 was selected for comparison with CD388, and we synthesised DFC with identical structure and DFR as reported by Cidara Therapeutics. As both exhibited excellent effects, we tested half the protective dose (0.05 mg/kg, Fig. 6k). AVC-P3 still protected 50% of the infected mice, whereas CD388 lost its protective efficacy with the same dose (Fig. 6l, m). Analysis of the structure-activity relationship of the AVCs revealed that the in vivo protective effect increased with AVC valence, with AVC-Z3 > AVC-Z2 > baloxavir. Considering the influence of the different NA inhibitors, AVC-P3 > AVC-Z3 > AVC-O3. AVC-P3 was selected as the candidate drug for further evaluation.

### Long-term and broad-spectrum protective effects of AVC-P3 against H1N1, H3N2 and H5N1 infection

**Fig. 7:**
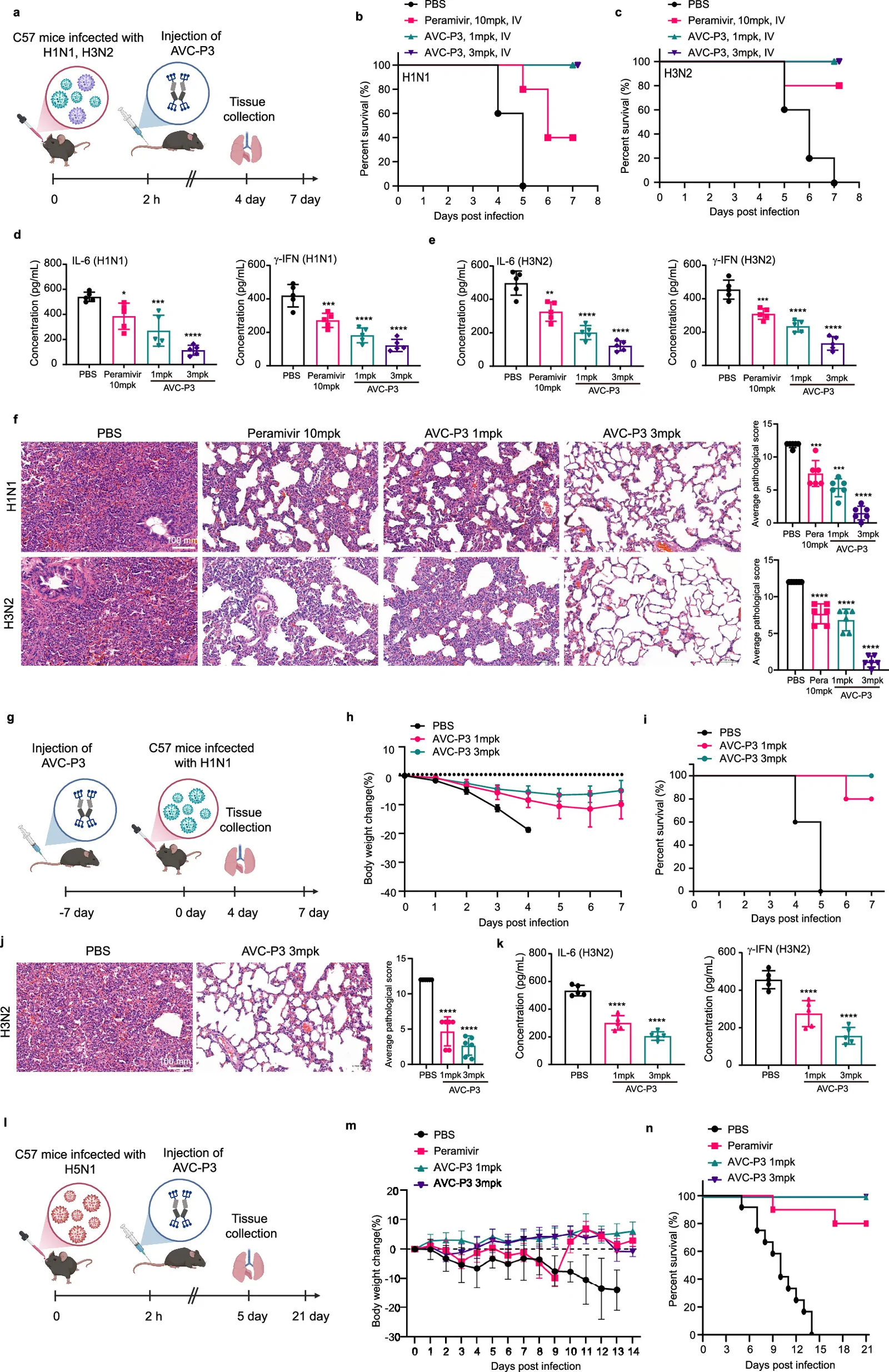
AVC-P3 demonstrated broad-spectrum protective effects against H1N1, H3N2 and H5N1 infection. **a** Experiment design of AVC-P3 in the H1N1 and H3N2 infection model. **b, c** %body weight change (**b**) and survival curve (**c**) in the H1N1 (A/California/07/2009) and H3N2 (A/Hong Kong/1/1968) infection model (*n*  =  5 mice per group). **d, e** For peramivir, AVC-P3 1 mpk, and 3 mpk vs PBS, lung cytokine levels of IL-6 (*P* = 0.0343, 0.0004, <0.0001) and γ-IFN (*P* = 0.0006, <0.0001, <0.0001) at 4 dpi in H1N1 (**d**); IL-6 (*P* = 0.0012, <0.0001, <0.0001) and γ-IFN (*P* = 0.0002, <0.0001, <0.0001) in H3N2 (**e**) infected mouse model, *n*  =  5 mice per group. **f** For peramivir, AVC-P3 vs PBS, H&E staining of lung tissue in the H1N1 (*P* = 0.0004, <0.0001) and H3N2 (*P* < 0.0001, *P* < 0.0001) infection model with AVC-P3, *n*  =  6 mice per group. **g** Prophylactic experiment design of AVC-P3 in the H1N1 infection model. **h, i** %body weight change (**h**) and survival curve (**i**) of pretreatment with AVC-P3 in the H1N1 infection model (*n*  =  5 mice per group). **j** H&E staining of lung tissue in H1N1 infection model pretreatment with AVC-P3 (*P* < 0.0001, AVC-P3 vs PBS). **k** Lung cytokine levels of IL-6 (*P* < 0.0001, AVC-P3 vs PBS) and γ-IFN (*P* < 0.0001, AVC-P3 vs PBS) at 4 dpi in the H1N1 infected mouse model, *n*  =  5 mice per group. **l** Experiment design of AVC-P3 in the H5N1 models. **m, n** %body weight change (**m**) and survival curve (**n**) of treatment with AVC-P3 in the H5N1 infection model (*n*  = 12 mice per group, except *n* = 10 mice in Peramivir group). Data are shown as mean ± s.d. *P* values were calculated using one-way ANOVA with Dunnett’s multiple comparisons test. NS not significant. Source data are provided as a Source Data file. Created in BioRender. Xiao, D. (2026) https://BioRender.com/dp6lrp9.

To evaluate the long-term and broad-spectrum protective effects of AVC-P3, we used C57BL/6 female mice (6–8 weeks age). The mice were intranasally infected with influenza virus (Fig. 7a). Two hpi, the mice received either a single intravenous injection of AVC-P3 (1.0 or 3.0 mg/kg) or peramivir (10 mg/kg, equivalent to 30.45 μmol/kg). Changes in mouse body weights and survival rates were monitored (Fig. 7b). Against H1N1 infection at 7 dpi, the maximum body weight loss in the peramivir and AVC-P3 (1 mg/kg) group was 15.08% and 7.82%, respectively, with no loss in AVC-P3 (3 mg/kg) group (Supplementary Fig. 17a). AVC-P3 at doses ≥1 mg/kg (≥0.015 μmol/kg) provided 100% protection, whereas 10 mg/kg peramivir (30.45 μmol/kg) offered only 40% protection (Fig. 7b). Against H3N2 infection, single-dose AVC-P3 at ≥1 mg/kg was superior to 10 mg/kg peramivir treatment, achieving 100% protection at 1 and 3 mg/kg (Fig. 7c, Supplementary Fig. 17b). A comparison of the molar-injected doses revealed that the AVC dose was only 1/600th to 1/6000th that of the NAIs, with significantly higher antiviral efficacy. Furthermore, in the same infection model, lung tissues were collected from mice treated with a single dose of AVC-P3 or peramivir on day 4 post-infection for histopathological examination (haematoxylin and eosin [H&E] staining) and cytokine measurements. In H1N1 and H3N2-infected mice (Fig. 7d, e), AVC-P3 significantly reduced IL-6 and IFN-γ secretion compared to those in the PBS group, with stronger inhibition at higher doses. Although peramivir significantly suppressed IFN-γ secretion in H1N1-infected mice, its IL-6 inhibition was less pronounced. H&E staining (Fig. 7f, Supplementary Fig. 17c, d) showed PBS and peramivir groups had thickened alveolar walls, disrupted lung architecture, extensive congestion, and inflammatory cell infiltration. In contrast, the mice treated with AVC-P3 displayed a clear lung structure with minimal congestion and inflammation. Lung tissue from the 3 mg/kg AVC-P3 group (4.69 × 10⁻² μmol/kg) showed no significant difference from uninfected (Mock) mice. These results indicate that AVC-P3 significantly prevented influenza-associated pneumonia and reduced inflammation-induced lung tissue damage.

Calculations based on a 20 g mouse receiving a 3 mg/kg AVC-P3 dose show that plasma concentrations after 10 half-lives (AVC-P3: ~105 days) would be 6.26 μM (AVC-P3). These concentrations remained above their respective antiviral EC values (<10 nM). These results demonstrate that, due to its excellent pharmacokinetics and superior antiviral efficacy, a single low dose of AVC-P3 can maintain plasma concentrations above the therapeutic threshold for extended periods, enabling it to function effectively as a prophylactic agent. Therefore, we explored its potential as a prophylaxis. C57BL/6 mice received a single intravenous dose of AVC-P3 7 days before intranasal challenge with H1N1 virus (1 × 10⁵ TCID₅₀). The survival rates and body weight changes were monitored (Fig. 7g). AVC-P3 treatment group showed no significant weight loss (Fig. h). AVC-P3 achieved 100% protection at doses ≥1 mg/kg (Fig. 7i). H&E staining (Fig. 7j, Supplementary Fig. 18) showed inflammatory cell infiltration and mild thickening of the alveolar walls with unclear structures in untreated mice. Analysis of lung tissue on day 4 post-infection revealed that AVC at doses ≥1 mg/kg significantly suppressed inflammatory cytokines (IL-6, IFN-γ) and reduced pneumonia risk (Fig. 7k). In contrast, mice receiving prophylactic AVC-P3 (3 mg/kg) displayed clear alveolar and bronchial structures with minimal inflammatory cell infiltration, indicating that AVC-P3 played a significant role in reducing lung tissue damage.

Finally, we explored the effect of AVC-P3 on H5N1 (HB3-E) infection, which has caused a real threat (Fig. 7l). The mice in the PBS group experienced continuous weight loss, and all died on the 13th day post-infection (Fig. 7m). High dose administration (10 mg/kg for 5 consecutive days, equivalent to 152.25 μmol/kg) of peramivir could not achieve 100% protection (Fig. 7n). AVC-P3 achieved 100% protection at doses ≥1 mg/kg, with no significant weight loss (Fig. 7n). AVC-P3 exhibited broad-spectrum and long-lasting antiviral activity, indicating its dual characteristics of prevention and treatment and was selected as a preclinical candidate compound for further research.

## Discussion

In this study, we leveraged click chemistry to construct a novel multivalent antiviral conjugates platform with modular programmability^33,34^, allowing flexible combination of valencies and conjugated drugs. This platform will benefit rapid drug development for the epidemic disease. Through our study, we made several discoveries.

First, we explored the impact of valency and NAIs on DFC activity. Results demonstrate that higher valence increases NA binding affinity, inhibition activity, and antiviral activity, with AVC-Z3 demonstrating the best activity. Thereafter, trivalent oseltamivir and peramivir were synthesised to generate AVC-O3 and AVC-P3. AVC-O3 did not exhibit comparable antiviral activity, possibly due to shielding of the C3-amino group of oseltamivir^35^. AVC-P3 demonstrated better antiviral activity than AVC-Z3 and bivalent CD388, indicating successful application of the multivalent design platform. For a more detailed comparison between trivalent AVC-P3 and CD388, the key distinctions or connections are as follows. In structural design, AVC-P3 selects the neuraminidase (NA) inhibitor peramivir and adopts a trivalent design to conjugate a total of 11 peramivir molecules, facilitating multivalent cross-linking with viruses. In compression, CD388 selects the NA inhibitor zanamivir and adopts a bivalent design to conjugate a total of 9 zanamivir molecules. Furthermore, the trivalent platform adopts a longer and more flexible linker design compared with CD388 (Supplementary Table 2). The flexible 39-atom central-linker of trivalent platform that separates the peramivir monomers by ~39 Å. However, the distance between zanamivir monomers of CD388 as ~18 Å. In the mechanism, the additional conjugated NAIs and the extended linker of the trivalent platform facilitated cross-linking with viruses, thereby coupling blockade of viral entry with restriction of progeny release. This cross-linking–mediated dual-stage inhibition represents a mechanistic shift from enzymatic suppression to physical viral immobilisation.

Second, we illustrated a dual-inhibition mechanism through AVC-Z3 and AVC-P3 cross-linking. TEM observations confirmed that they could simultaneously bind to multiple viral particles, leading to viral aggregation with a higher AVC valence and larger aggregates. This aggregation effect retains the virus on the cell surface and prevents invasion in the early stages. Viruses are colloidal particles with limited half-lives and no motility. They must diffuse to host cells before decaying^36^. Multivalent engagement aggregation by AVC-P3 hinders diffusion and reduces both the surface-to-volume ratio and the number of infectious particles, decreasing adsorption and the fraction of cells reachable. This dual-inhibition mechanism contributes to increased activity. To the best of our knowledge, this is the first study to demonstrate that NAI-based drugs can act on the early viral infection stages.

Third, the prolonged half-life of AVC-P3 is an unexpected advantage. For antibody–drug conjugates and DFC (CD388), high drug-antibody ratios and DFRs usually limit the half-life and effect of the conjugates in vivo. However, trivalent peramivir conjugated with Fc with a DFR 3.7, conjugating 12 peramivir per Fc, exhibited a half-life of over 200 h, surpassing CD388 with YTE mutation in Fc. The long half-life was related to the high similarity between the structures of peramivir and sialic acid^37^. Sialic acid residues provide negative charges to antibodies, reducing nonspecific cell membrane interactions and receptor-mediated endocytosis, decreasing clearance^38,39^. Ludwig et al. increased anti-IL-2 IgG sialylation using 1,3,4-O-Bu₃ManNAc, extending its plasma half-life from 39.5 h to 60.9 h^40^.

Lastly, AVC-P3 demonstrated superior antiviral efficacy against H1N1, H3N2, H5N1, and IFV/B/Lee/40 infections in vivo, compared to that of AVC-Z3 and CD388. Even at 1/600 to 1/6000 of single-dose NAI treatment, AVC-P3 exhibited stronger antiviral effects, suggesting its potential clinical application in the future. In conclusion, AVC-P3 represents a novel “biochemical vaccine” strategy, providing immediate therapeutic and prophylactic effects, without the need for the 2–4-week immune cycle of traditional influenza vaccines^41,42^. AVC-P3 may be the most powerful anti-influenza drug, offering significant potential to prevent influenza in older adults and children with low immunity.

## Methods

### Ethics statement

All animal experiments involving virus infection in this study were approved by the Ethics Committee of the Guangdong–Hong Kong Joint Laboratory of Emerging Infectious Diseases (Approval No. SUMC2022-051) and the WuXi Institutional Animal Care and Use Committee (Approval No. ID01-QD008-2023v1.2). All experiments were performed in accordance with relevant institutional and national guidelines and regulations.

### NA inhibition assay

Dilute NA (A/Puerto Rico/8/1934, A/California/04/2009 or A/California/04/2009-H275Y mutation, purchased from Sino Biological, Beijing, China) to 20 U/mL in assay buffer. Prepare 3-fold serial dilutions of the test compound in assay buffer. Mix 25 ul 20 U/mL NA with 25 ul series dilutions of test compound, incubate at room temperature for 30 min. Dilute the substrate to 400 μM in assay buffer, and start the reaction by adding 50 μL of 400 μM substrate. Read at excitation and emission wavelengths of 365 nm and 445 nm (Molecular Devices, SpectraMax Gemini EM), respectively, in kinetic mode for 20 minutes. The IC_50_ values were calculated using GraphPad Prism 10.2.

### AVC-NA binding affinity assay

NA protein was resuspended to 200 µg/mL, and the chip was activated with 400 mM EDC and 100 mM NHS (1:1 ratio) for 420 seconds at 10 µl/min. NA protein was diluted to 20 µg/mL in acetate buffer (pH 5.0) and injected into the flow channel for 420 seconds at 10 µl/min. Unbound active sites were blocked with 1 M ethanolamine (pH 8.5) for 420 seconds. The final resonance signal reached 4200 RU. Test samples were prepared in a concentration gradient of 100 nM to 3.125 nM using running buffer (PBS + 0.5% Tween). Each sample was injected sequentially into the channels at 30 µl/min for 120 seconds. NaOH (5 mM) was used to dissociate the samples at 30 µl/min. The chip was regenerated using acetate buffer (pH 5.0) at 30 µl/min for 30 seconds. Data were collected using the Biacore 8 K system (Cytiva) and analyzed (Biacore Insight Evaluation Software, Cytiva, version 5.0.18.22102) using a 1:1 binding model to fit the affinity curve^10^.

### Cells and virus

MDCK cells were obtained from the Institute of Basic Medicine, Chinese Academy of Medical Sciences (Beijing, China), and cultured in DMEM (GIBCO BRL, Grand Island, NY, USA), supplemented with 10% FBS (GIBCO BRL, Grand Island, NY, USA), 100 U/mL penicillin, and 100 μg/mL streptomycin, under conditions of 37 °C and 5% CO₂. The influenza viruses were amplified in the chorioallantois cavity of 10-day-old chicken embryos at 37 °C for 48 h. The influenza viruses were cultured for 3 days in MDCK cells in serum-free Dulbecco’s Modified Eagle’s Medium/Ham’s F-12 medium (DF-12) with 2 μg/mL tosylamido-phenylethyl-chloromethyl-ketone-treated trypsin (TPCK-trypsin; Sigma, Kawasaki, Japan), and the culture temperature was 37 °C. A/ Puerto Rico/8/1934 (H1N1), A/California /07/2009 (H1N1), A/Hong Kong/1/1968 (H3N2), B/Florida/78/2015 (B/V), A/Liaoning-Zhenxing/1109/2010 (H1N1, NAIs-resistant strain) and A/ Puerto Rico/8/1934 (I38T mutation, H1N1-baloxavir resistant strain) were stored in our lab; A/Victoria/2570/2009 (H1N1), A/California/04/2009 (H1N1), A/California/07/2009 (H1N1), A/Hong Kong/1/1968 (H3N2), A/Vietnam/2004 (H5N1), A/Shanghai/02/2013 (H7N9), B/Florida/04/2006 (BY) and B/Phuket/3073/2013(BY) were stored in Professor Xia’s lab of Xiamen University.

### Anti-influenza effects of NAIs and AVC

MDCK cells were seeded in a 96-well plate (5 × 10⁴ cells/well) and cultured for 24 h. Cells were infected with influenza virus at 100 TCID_50_/well, followed by the addition of NAIs or AVCs in serial dilutions (for Baloxavir Marboxil and NAIs-zanamivir, Ostamivir, Peramivir, 0.15–1000 nM; for AVC, 0.02–100 nM). After incubation at 37 °C for 3 days, the antiviral effect was determined using CellTiter-Glo cell viability assay reagent (Promega, Madison, WI, USA), and the EC_50_ values were calculated using GraphPad Prism 10.2.

### Plaque and TCID_50_ assay

#### Plaque assay

MDCK cell monolayers in 12-well plates were inoculated with serially diluted virus (10-fold increments) and incubated at 37 °C for 1.5 h. The viral inoculum was then removed, and the cells were overlaid with DF-12 medium containing 1% agarose, 1 μg/mL TPCK-trypsin, 100 U/mL penicillin, and 100 μg/mL streptomycin. After further incubation at 37 °C for 72 h, the cells were fixed with 4% formaldehyde for 4 h and stained with 1% crystal violet for plaque counting.

#### TCID₅₀ assay

MDCK cell monolayers in 96-well plates were washed with PBS, followed by the addition of DF-12 medium containing 2 μg/mL TPCK-trypsin to each well. The test virus was serially diluted 10-fold (ranging from 10⁰ to 10⁻⁸), and each dilution was inoculated into MDCK cells in the 96-well plates. After incubation at 37 °C for 72 h, the 50% tissue culture infectious dose (TCID₅₀) was calculated using the Reed–Muench method.

### Protein cross-linking size analysis

To determine whether AVC can bind multiple NA proteins simultaneously, we measured the change in particle size upon binding. Test samples at 2 µM and 6 µM concentrations were prepared in water, along with N1 (H1N1, A/Sydney/5/2021) protein at 6 µM. The samples were mixed at a 1:6 molar ratio and incubated at room temperature for 1 h before particle size measurements were taken.

### Protein cross-linking TEM analysis

To explore whether AVC can cause NA protein aggregation, transmission electron microscopy (TEM) was used to observe changes in NA upon binding with the drug. Samples of 2 µM and 6 µM AVC and 6 µM N1 (H1N1, A/Sydney/5/2021) protein were mixed at a 1:6 molar ratio and incubated at room temperature for 1 h. Negative staining was performed on the samples, which were then imaged using a transmission electron microscope (JEM-1400, Japan).

### Viral cross-linking TEM analysis

To investigate whether AVC induces viral aggregation, influenza virus (10⁷ pfu/150 µl) was incubated with AVC (1 mM in PBS, 10 µl) in DMEM for 3 h at 37 °C. An equal volume of 4% paraformaldehyde was added to fix the samples for 15 minutes. A 10 µl aliquot was transferred onto carbon-coated copper grids, left to stand at room temperature for 10 min, and the excess liquid was removed. The sample was negatively stained with 1% uranyl acetate (30 µl), air-dried, and analyzed by TEM (JEM-1400, Japan).

### Scanning electron microscopy of AVC inhibition of viral release

MDCK cells were seeded onto round coverslips (8 mm diameter) in 24-well plates at 2 × 10⁴ cells/mL (1 mL/well) and incubated overnight. The next day, cells were infected with A/Puerto Rico/8/1934 (H1N1) virus at MOI:10 for 1 h. Unbound virus was washed off with PBS, and cells were incubated with the test drug for 10 h. The cells were then fixed with 2.5% glutaraldehyde and 2% paraformaldehyde for 2 h, followed by ethanol gradient dehydration (30%-100%) in 5–10-minute steps. Samples were air-dried and coated for SEM imaging.

### Pharmacokinetic analysis

To determine the pharmacokinetic properties of AVC-Z3 and AVC-P3 in mice, 6-8-week-old female healthy BALB/c mice (3 or 4 per group) were administered 25 mg/kg of the test drug via tail vein injection. Blood samples (20 µl) were collected at various time points (0.2 h, 2 h, 5 h, 9 h, 1 d, 3 d, 5 d, 7 d, 10 d, 14 d) and mixed with an anticoagulant (saturated EDTA-Na_2_). FcRn knock-in mice (6 per group) were administered 5 mg/kg of the test drug via tail vein injection. Blood samples (20 µl) were collected at various time points (4 h, 24 h, 48 h, 72 h, 120 h, 168 h, 216 h, 288 h, 360 h, 432 h, 504 h, 576 h, 672 h, 796 h) and mixed with an anticoagulant (saturated EDTA-Na_2_). After centrifugation (2000 g, 10 min), plasma was collected for testing. A standard curve was prepared using NA protein-coated 96-well plates and different concentrations of AVC. The drug concentration in plasma was measured via sandwich ELISA using monoclonal anti-human-IgG-Fc antibody (1:500, clone: 6F11C8, CAT: IGG-S307, ACRO Biosystems) for capture and goat anti-human IgG (Fc) polyclonal antibody for test (1:1000, SeraCare, CAT:5220-0279). Only female mice were used in all animal experiments in this study, as they are less aggressive during group housing and sex-dependent differences have not been established for the infection models used. All mice were housed in a specific pathogen-free (SPF) facility under controlled environmental conditions, with a 12-hour light/dark cycle and ad libitum access to standard rodent diet and water.

### In vivo antiviral activity of AVC in BALB/c mice model and C57 mice model

To assess whether AVC can inhibit viral replication in the lungs and nasal turbinates of mice, 6-8-week-old female BALB/c mice and C57 mice were intranasally inoculated with H1N1 virus, H3N2 virus, H5N1 virus, or B/Lee/40. Two h post-infection, the indicated doses of AVC (0.05 mg/kg, 1 mg/kg, or 3 mg/kg), PBS, or negative control Fc protein (JS95A, 3 mg/kg) were administered intravenously. Positive control peramivir (10 mg/kg) was administered intravenously once, while oseltamivir (20 mg/kg) and baloxavir marboxil (5 mg/kg) was given orally twice daily for 5 days. On day 4 post-infection, the mice were euthanized, and viral loads in the lungs were measured. Weight loss, survival, and H&E staining of lung tissue were also monitored, with a weight loss of 35% considered fatal.

### In vivo antiviral activity of AVC in DBA/2 mouse model

To evaluate AVC’s efficacy in severe influenza, we used the DBA/2 mouse model, which is highly susceptible to influenza. Six-week-old female DBA/2 mice (10 per group) were intranasally inoculated with 1 × 10⁴ PFU of H1N1. Two h post-infection, single doses of AVC (3 mg/kg), PBS, or negative control Fc protein (JS95A, 3 mg/kg) were administered intravenously. Positive control zanamivir (5 mg/kg) was administered intravenously daily for 5 days, while oseltamivir (20 mg/kg) was given orally twice daily for 5 days. Weight loss and survival were monitored for 14 days, with a weight loss of 35% considered fatal. Statistical analyses and curve-fitting were performed using GraphPad Prism (version 10.2).

### Reporting summary

Further information on research design is available in the Nature Portfolio Reporting Summary linked to this article.

## Supplementary information

Supplementary Information

Reporting Summary

Transparent Peer Review file

## Source data

Source data

## Data Availability

All data generated in this study are provided within the paper and its Supplementary Information. Source data are provided with this paper.
