# Supplementary material for: Multivalent antiviral conjugates enhance activity against Influenza via cross-linking viruses
Source: Nat Commun. 2026 Oct 4;17:11320. doi: 10.1038/s41467-026-78126-3 (PMC13724737; doi:10.1038/s41467-026-78126-3)
Supplement: Supplementary file 1 [file 41467_2026_78126_MOESM1_ESM.pdf]

Supplementary Information

Multivalent anti-viral conjugates enhance activity against Influenza via cross-linking viruses

Xun Feng[1,2,3†], Lunzhi Yuan[4†], Lei Zhao[2†], Lianqi Liu[1,2†], Fei Xie[1,2], Ming Zhou[4], Gong Cheng[3], Yi Guan[5,6,7], Ningshao Xia[4], Tong Cheng[4*], Wu Zhong[1,2*], Dian Xiao[1,2*], Xinbo Zhou[1,2*] and Song Li[1,2]

This file includes

Supplementary Figure 1. Synthesis of I-7 (Z1).

Supplementary Figure 2. Synthesis of I-11a (Z2).

Supplementary Figure 3. Synthesis of I-11b (Z3).

Supplementary Figure 4. Synthesis of II-7 (P3).

Supplementary Figure 5. Synthesis of II-7 (O3).

Supplementary Figure 6. Conjugation of AVCs.

Supplementary Figure 7. Synthesis of negative control AVC-Z1-S.

Supplementary Method 1 Synthetic methods of Z1, Z2, Z3, P3 and O3.

Supplementary Method 2 Synthetic methods of AVCs.

Supplementary Figure 8 Characterizations of Fc.

Supplementary Figure 9 Purity analysis of Z1, Z2, Z3, P3 and O3.

Supplementary Figure 10 Structures of AVCs.

Supplementary Figure 11 Size-Exclusion Chromatography analysis of AVCs.

Supplementary Figure 12 SPR analysis of zanamivir and AVCs.

Supplementary Figure 13 TEM micrographs of cell.

Supplementary Figure 14 The entry blockade assays of AVC-Z1H-S with short linker.

Supplementary Figure 15 Characterization of AVCs.

Supplementary Figure 16 ADCC, ADCP effects analysis of AVC-P3, CD388 and Fc.

Supplementary Figure 17 Treatment efficacy of AVC-P3 in lethal mouse models of H1N1 and H3N2 infection.

Supplementary Figure 18 Hematoxylin and eosin staining of lung tissue in AVC-3 pretreatment group.

Supplementary Table 1 The antivirus activities of oseltamivir, AVC-O3 and short linker control AVC-Z1H-S.

Supplementary Table 2 Comprehensive linker information of different AVCs.

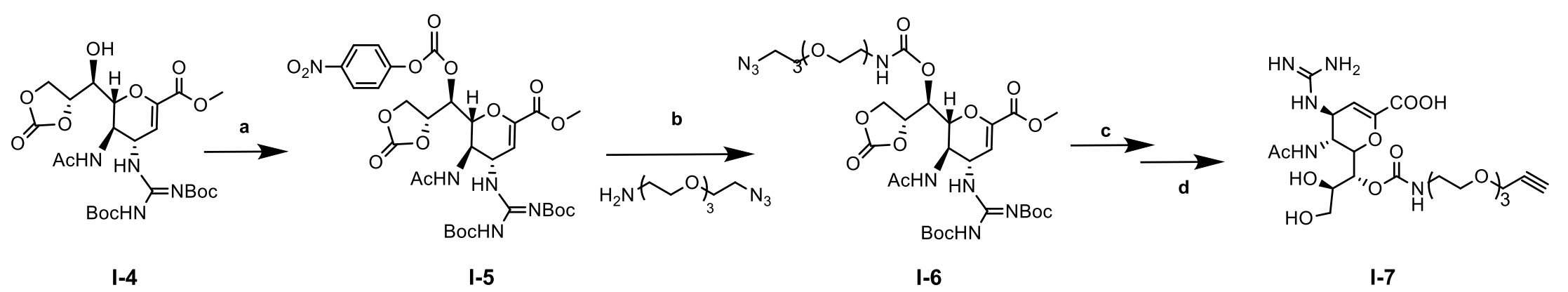

Supplementary Figure 1. Synthesis of I-7 (Z1). (a) 4 dimethyl amino pyridine, DIPEA, 4-Nitrophenyl chloroformate, DCM, 0℃, yield:70%; (c) HOBT, DIPEA, DCM, r.t., 12 h, Yield:80%; (c) TFA, DCM, r.t., 4 h, Yield:55%; (d) NaOH, THF/H_2O, 4N HCl/dioxane, 0 ℃, 4 h, Yield: 40%.

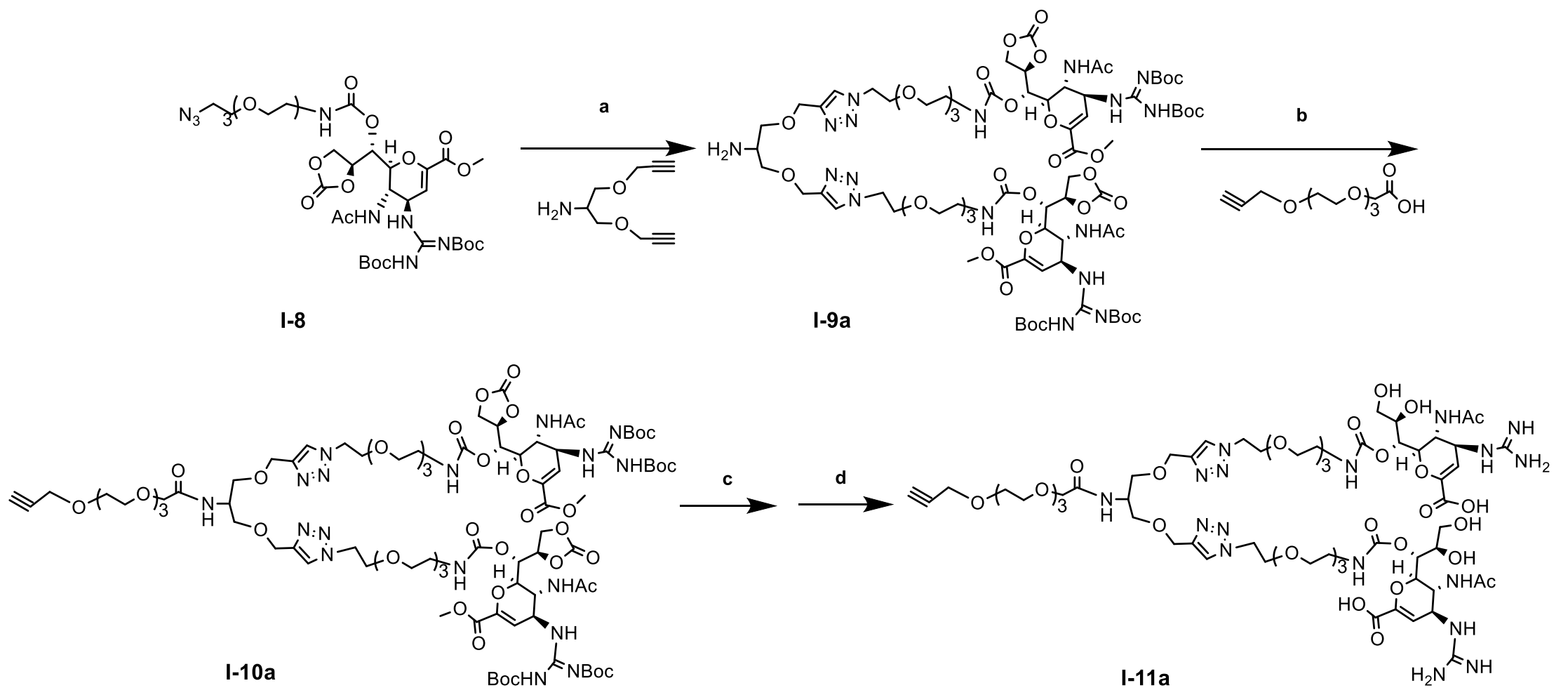

Supplementary Figure 2. Synthesis of I-11a (Z2). (a) $CuSO_4 \cdot 5\ H_2O$, Sodium ascorbate, THF/H_2O, r.t., 4 h, Yield:54%; (b) EDCI, HOBT, DMAP, DIPEA, DMF, r.t., 24 h, Yield:78%; (c) TFA, DCM, r.t., 4 h, Yield:55%; (d) NaOH, THF/H_2O, 4N HCl/dioxane, 0 ℃, 4 h, Yield: 40%

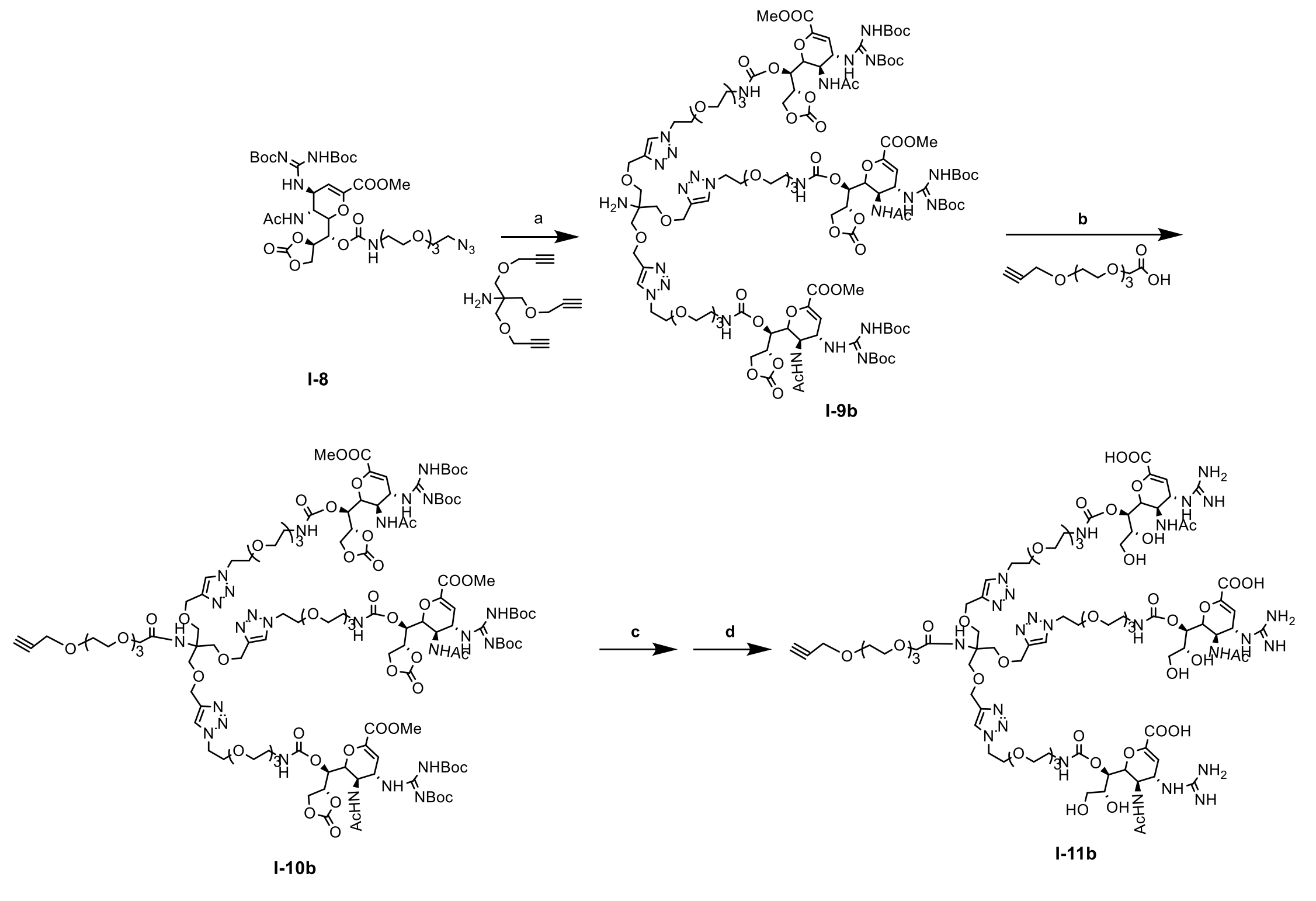

Supplementary Figure 3. Synthesis of I-11b (Z3). (a) CuSO4·5 H2O, Sodium ascorbate, THF/H2O, r.t., 4 h, Yield:54%; (b) EDCI, HOBT, DMAP, DIPEA, DMF, r.t., 24 h, Yield:78%; (c) TFA, DCM, r.t., 4 h, Yield:55%; (d) NaOH, THF/H2O, 4N HCl/dioxane, 0 °C, 4 h, Yield: 40%.

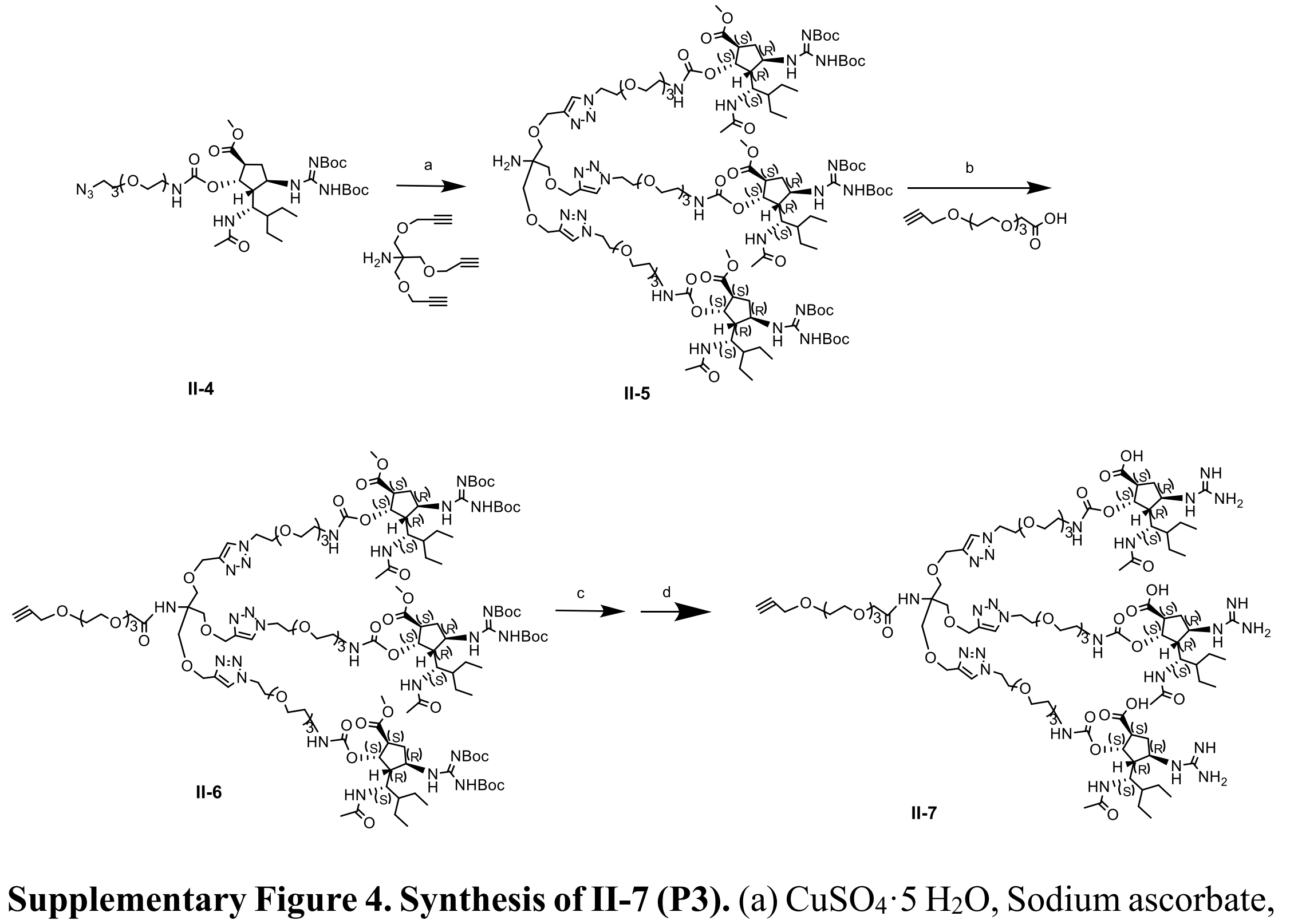

Supplementary Figure 4. Synthesis of II-7 (P3). (a) $CuSO_4 \cdot 5\ H_2O$, Sodium ascorbate,

THF/H_2O, r.t., 4 h, Yield:46%; (b) EDCI, HOBT, DMAP, DIPEA, DMF, r.t., 24 h, Yield:90%; (c) TFA, DCM, r.t., 4 h, Yield:65%;(d) NaOH, THF/H_2O, 4N HCl/dioxane, 0 °C, 4 h, Yield: 40%.

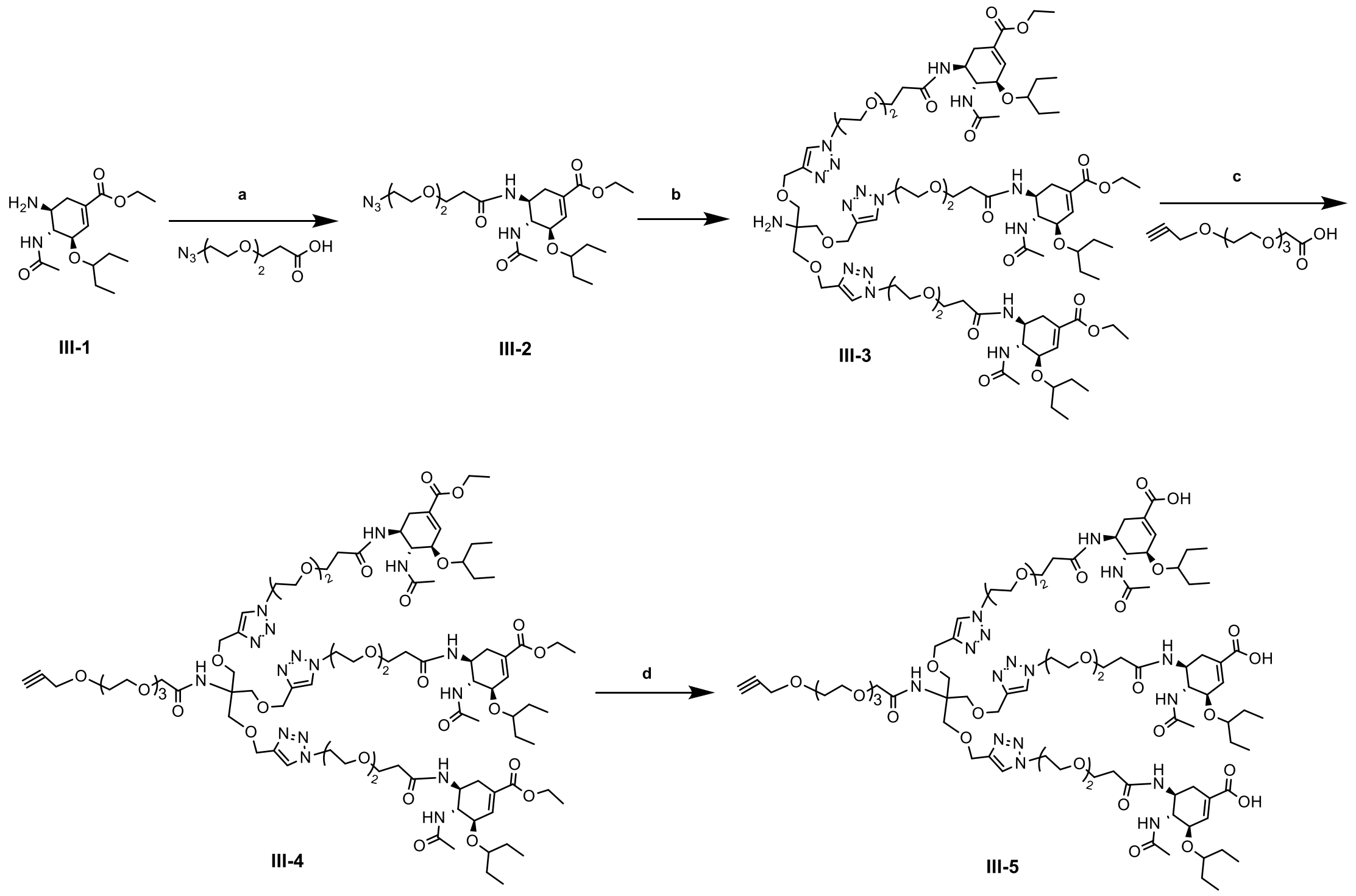

Supplementary Figure 5. Synthesis of II-7 (O3). (a) EDCI, HOBT, DMAP, DMF, r.t., 24 h, Yield: 82%; (b) $CuSO_4 \cdot 5\ H_2O$, Sodium ascorbate, THF/H_2O, r.t., 4 h, Yield:50%; (c) EDCI, HOBT, DMAP, DIPEA, DMF, r.t., 24 h, Yield:89%; (d) NaOH,MeOH, 2N HCl, r.t., 4 h, Yield: 46%.

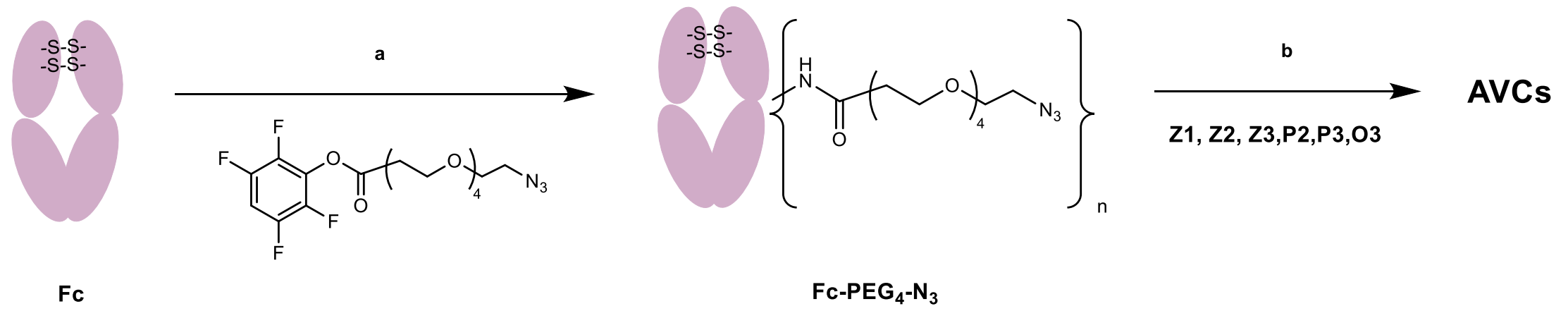

Supplementary Figure 6. Conjugation of AVCs. (a) PBS, r.t., 12 h, Yield:80%; (b) CuSO4-5H2O, Sodium Ascorbate, THPTA, r.t., 4 h, Yield:80%.

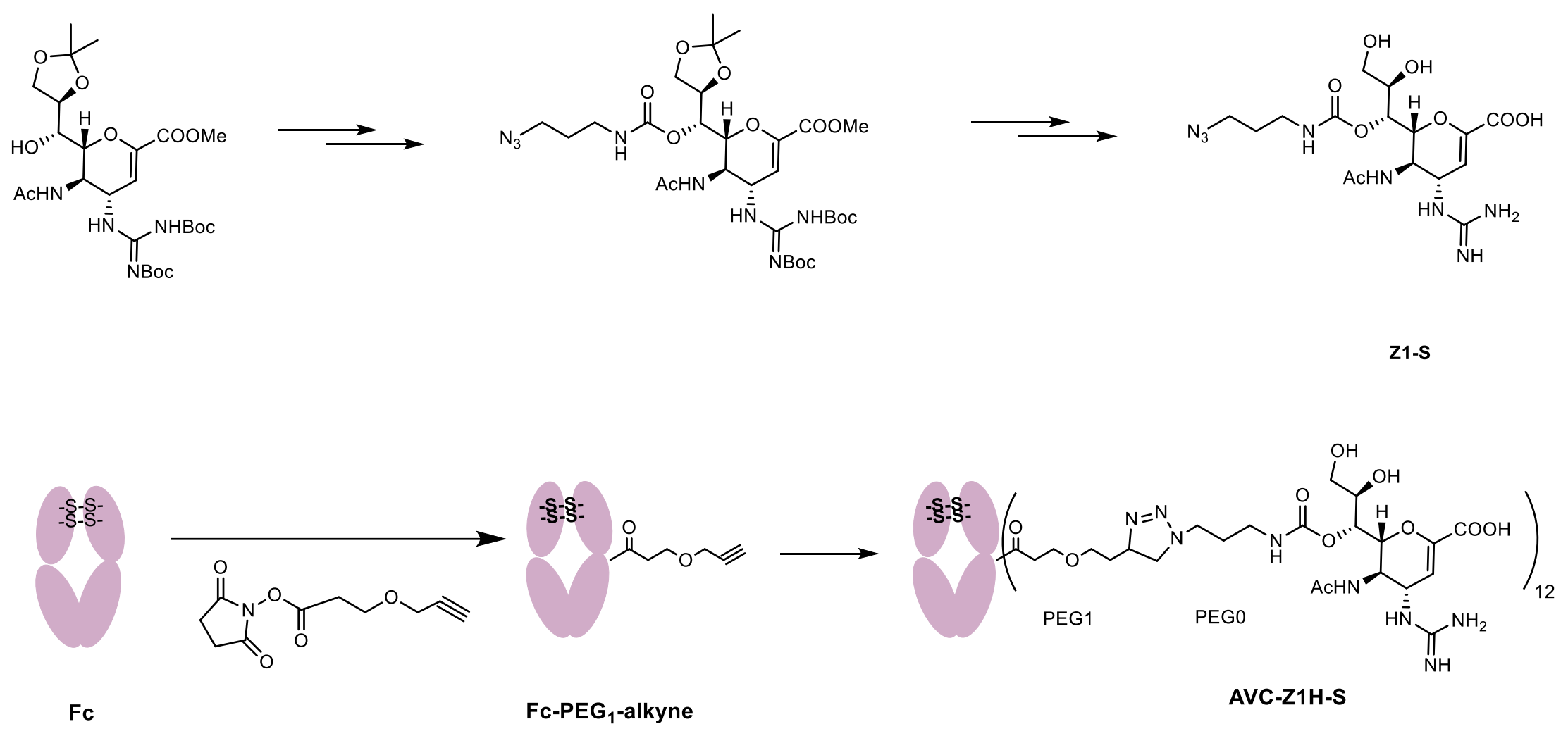

Supplementary Figure 7. Synthesis of negative control AVC-Z1-S. The reactions and conditions are the same as AVC-Z1.

Compounds I-4

The compound I-3(1 g, 1.82 mmol) was dissolved in anhydrous dichloromethane (DCM, 6 mL) and cooled in an ice water bath, and then 4-dimethylaminopyridine (DMAP, 221.26 mg, 1.82 mmol) and N,N-Diisopropylethylamine (DIPEA, 728 mg, 5.6 mmol) were added. Then 4-nitrophenyl chloroformate (537.75 mg, 3.05 mmol) was added to the reaction solution drop by drop. The ice bath was then removed, the reaction liquid stirred for 3 h and monitored by LCMS (with the addition of 4-nitrophenyl chloroformate if needed). After adding water (10 mL) to end the reaction, the organic layer is separated and concentrated by a rotary evaporator, and then purified by C18 reversed phase column chromatography (100 g, 20 % to 70 % acetonitrile). The acetonitrile in collected eluent was removed by a rotary evaporator at room temperature and then extracted with a 1:1 mixture of ethyl acetate and hexane. The organic phase added anhydrous Na_2SO_4 and dry for 8 h. The white solid product (712 mg, 68.34 %) was obtained by vacuum drying after concentration by rotary evaporator. ^{1}H NMR (600 MHz, DMSO-*d*$_6$) δ 11.39 (s, 1H), 8.26 (d, *J* = 7.7 Hz, 1H), 8.15 (d, *J* = 8.3 Hz, 1H), 5.81 (d, *J* = 2.4 Hz, 1H), 5.72 (d, *J* = 5.9 Hz, 1H), 4.98 (ddd, *J* = 8.4, 6.8, 3.3 Hz, 1H), 4.88 (ddd, *J* = 10.1, 7.7, 2.5 Hz, 1H), 4.60 (d, *J* = 32.3 Hz, 0H), 4.61 – 4.53 (m, 2H), 4.18 (dd, *J* = 10.5, 1.6 Hz, 1H), 4.10 (td, *J* = 10.1, 8.4 Hz, 1H), 4.01 (ddd, *J* = 6.0, 3.4, 1.6 Hz, 1H), 3.73 (s, 3H), 1.85 (s, 3H), 1.47 (s, 9H), 1.47 (d, *J* = 16.0 Hz, 0H), 1.40 (s, 9H). $[M+H]^+ = 573.24$

Compounds I-5.

The compound I-4 (700 mg, 1.22 mmol) was dissolved in 5 mL ultra-dry DCM,

cooled in an ice water bath, and then added 4-nitrophenyl chloroformate (294.6 mg, 1.46 mmol), then added DMAP (123.2 mg, 0.61 mmol), nitrogen protection. Continue stirring after removing the ice bath for 3 h. After the reaction was completed, the solvent was removed by rotary evaporator, and the product (540 mg, 60 %) was purified by column chromatography. ^{1}H NMR (600 MHz, DMSO-d_6) δ 11.41 (s, 1H), 8.37 – 8.34 (m, 2H), 8.26 – 8.23 (m, 1H), 7.62 – 7.58 (m, 2H), 6.95 – 6.91 (m, 1H), 5.88 (d, *J* = 2.4 Hz, 1H), 5.36 (td, *J* = 8.4, 8.0, 2.6 Hz, 1H), 4.93 (ddd, *J* = 10.3, 8.0, 2.5 Hz, 1H), 4.73 (t, *J* = 8.8 Hz, 1H), 4.61 (dd, *J* = 9.3, 7.3 Hz, 1H), 4.54 (dt, *J* = 13.4, 4.1 Hz, 2H), 4.31 (q, *J* = 10.0 Hz, 1H), 3.76 (s, 3H), 1.79 (s, 3H), 1.46 (s, 9H), 1.41 (s, 9H). $[M+H]^+$ = 738.23

Compounds I-6.

The compound I-5 (240 mg, 0.33 mmol) was dissolved in 5 mL of anhydrous DCM, followed by the addition of alkynyl-PEG$_3$-amine (61.7 mg, 0.33 mmol), 1-hydroxybenzotriazole (HOBT, 43.96 mg, 0.33 mmol), DIPEA (84.11 mg, 0.65 mmol), nitrogen protection, room temperature stirring, reaction for 8 h. The product (129 mg, 49 %) was obtained by column chromatography. ^{1}H NMR (600 MHz, Chloroform-d_6) δ 11.39 (s, 1H), 8.48 (d, *J* = 8.5 Hz, 1H), 6.68 (d, *J* = 9.3 Hz, 1H), 5.92 (d, *J* = 2.4 Hz, 1H), 5.65 (t, *J* = 5.8 Hz, 1H), 5.54 (dd, *J* = 4.4, 2.0 Hz, 1H), 5.21 (d, *J* = 8.0 Hz, 1H), 5.03 (td, *J* = 7.8, 4.2 Hz, 1H), 4.73 – 4.60 (m, 2H), 4.44 (dd, *J* = 10.6, 2.0 Hz, 1H), 4.21 (d, *J* = 2.4 Hz, 2H), 4.16 (q, *J* = 10.0 Hz, 1H), 3.79 (s, 3H), 3.73 – 3.24 (m, 12H), 2.47 (t, *J* = 2.4 Hz, 1H), 1.91 (s, 3H), 1.48 (s, 9H), 1.46 (s, 9H). $[M+H]^+$ = 786.34

Compounds I-7 (Z1).

The compound I-6 (129 mg, 0.16 mmol) is dissolved in 2 mL of anhydrous DMF, followed by the addition of 2 mL of trifluoroacetic acid (TFA). The mixture is stirred at room temperature for 4 h, then TFA is removed using a rotary evaporator. The product is purified by C18 reversed-phase chromatography, yielding 80 mg with 85 % yield. The product (80 mg, 0.136 mmol) is dissolved in 1.5 mL of methanol, to which 200 µl of double-distilled water is added. The pH is adjusted to 12-13 with a 10 M NaOH solution, and the mixture is stirred at room temperature for 4 h. The pH of the reaction system is then adjusted to 4 using a 4 M hydrochloric acid solution in 1,4-dioxane. Finally, the product is purified by C18 reversed-phase chromatography, yielding 40 mg with a 53.8 % yield. ^{1}H NMR (600 MHz, Deuterium Oxide) δ 4.45 (t, *J* = 10.2 Hz, 1H), 4.34 (d, *J* = 8.9 Hz, 1H), 4.17 (dq, *J* = 2.3, 1.1 Hz, 6H), 3.63 – 3.59 (m, 12H), 3.54 (tt, *J* = 10.5, 5.6 Hz, 6H), 3.25 (d, *J* = 5.4 Hz, 2H), 3.24 (s, 1H), 2.82 (t, *J* = 2.1 Hz, 2H), 1.97 (d, *J* = 1.2 Hz, 3H), 1.91 (s, 1H). ^{13}C NMR (201 MHz, D_2O) δ 174.33, 168.97,

158.55, 156.99, 149.06, 103.91, 79.30, 75.95, 75.17, 69.56, 69.41, 69.37, 69.28, 68.64, 67.86, 67.59, 66.27, 57.87, 51.08, 47.72, 40.05, 22.02. $[M+H]^+$ = 546.449

Compounds I-8.

The compound I-5 (240 mg, 0.33 mmol) was dissolved in 5 mL of anhydrous DCM, followed by the addition of Azide-PEG3-amine (N_3-PEG_3-NH_2, 71 mg, 0.33 mmol), HOBT (43.96 mg, 0.33 mmol), DIPEA (84.11 mg, 0.65 mmol), nitrogen protection, room temperature stirring, reaction for 8h. The product (140 mg, 51 %) was obtained by column chromatography. ^{1}H NMR (600 MHz, Chloroform-d_6) δ 7.68 – 7.58 (m, 1H), 7.36 – 7.25 (m, 1H), 4.44 – 3.17 (m, 8H), 2.27 (t, *J* = 7.6 Hz, 1H), 1.60 (dp, *J* = 54.0, 7.3 Hz, 2H), 1.38 (ddd, *J* = 29.7, 19.8, 15.3 Hz, 3H), 1.18 (s, 32H), 0.89 (t, *J* = 7.4 Hz, 1H), 0.81 (t, *J* = 7.0 Hz, 4H). $[M+H]^+$ = 817.34

Compounds I-7-S (Z1-S).

The short linker control (**I-7-S**) was synthesized as **I-7.** ^{1}H NMR (500 MHz, DMSO-d6) δ ppm 12.02 – 14.31 (m, 1 H), 8.01 (br d, *J* = 9.31 Hz, 1 H), 7.63 (br d, *J* = 8.85 Hz, 1 H), 7.31 (br t, *J* = 5.72 Hz, 1 H), 5.70 (d, *J* = 2.14 Hz, 1 H), 5.10 (br s, 1 H), 4.83 (dd, *J* = 9.31, 1.53 Hz, 1 H), 4.47 (br d, *J* = 2.29 Hz, 1 H), 4.37 (br dd, *J* = 10.30, 1.45 Hz, 1 H), 4.30 – 4.35 (m, 1 H), 4.02 (q, *J* = 9.46 Hz, 1 H), 3.81 (br t, *J* = 7.10 Hz, 1 H), 3.30 – 3.53 (m, 6 H), 3.24 (br dd, *J* = 11.22, 7.71 Hz, 1 H), 2.99 – 3.09 (m, 1 H), 2.88 – 2.97 (m, 1 H), 1.79 (s, 3 H), 1.55 – 1.70 (m, 2 H). ^{13}C NMR (126 MHz, DMSO-d6) δ ppm 169.79, 163.11, 159.19, 157.55, 156.04, 145.43, 117.54, 108.97, 76.36, 69.84, 69.39, 63.61, 51.28, 48.62, 46.53, 38.04, 29.00, 23.06. $[M+H]^+$ = 459.3

Compounds I-9a.

Dissolving compound I-8 (140 mg, 0.168 mmol) and compound A4 (11.29 mg, 0.0672 mmol) separately in 1.5 mL of THF, mixing and stirring, adding a water solution of $CuSO_4\cdot 5H_2O$ (6.294 mg, 0.025 mmol) and sodium ascorbate (Na·VC, 9.98 mg, 0.05 mmol) sequentially to the reaction mixture, reacting at room temperature for 3 h, the mixture is extracted with DCM. The final product is obtained as 100 mg after purification by column chromatography, 33 % yield, Chemical Formula: $C_{75}H_{117}N_{17}O_{34}$, ^{1}H NMR (600 MHz, DMSO-d_6) δ 8.20 (d, *J* = 7.7 Hz, 2H), 8.09 (s, 2H), 7.96 (d, *J* = 9.1 Hz, 2H), 7.38 (t, *J* = 5.8 Hz, 2H), 5.85 (d, *J* = 2.4 Hz, 2H), 5.46 (t, *J* = 2.5 Hz, 2H), 5.17 (td, *J* = 7.7, 2.9 Hz, 2H), 4.82 – 4.75 (m, 2H), 4.62 (t, *J* = 8.6 Hz, 2H), 4.56 (s, 3H),

4.45 (dd, *J* = 10.6, 2.0 Hz, 2H), 4.01 (q, *J* = 10.1 Hz, 2H), 3.81 (t, *J* = 5.3 Hz, 4H), 3.74 (s, 6H), 3.61 – 3.35 (m, 28H), 3.08 – 3.02 (m, 4H), 1.74 (d, *J* = 3.7 Hz, 6H), 1.45 (d, *J* = 3.1 Hz, 18H), 1.40 (s, 18H), 1.34 (d, *J* = 9.2 Hz, 2H), 1.27 – 1.21 (m, 7H), 0.88 – 0.83 (m, 2H). $[M+H]^+$ = 1800.8317

Compounds I-9b.

Dissolving compound I-8 (140 mg, 0.168 mmol) and compound B4 (12.34 mg, 0.0525 mmol) separately in 1.5 mL of THF, mixing and stirring, adding a water solution of $CuSO_4 \cdot 5H_2O$ (6.294 mg, 0.025 mmol) and Na·VC (9.98 mg, 0.05 mmol) sequentially to the reaction mixture, reacting at room temperature for 3 h, extracting with DCM, and finally purifying by column chromatography to obtain the product 106 mg. 25.5 % yield. ^{1}H NMR (600 MHz, Chloroform-*d*) δ 11.38 (s, 2H), 8.43 (d, *J* = 7.9 Hz, 2H), 7.88 (s, 1H), 7.16 (s, 1H), 5.97 (d, *J* = 2.4 Hz, 1H), 5.68 (s, 1H), 5.62 (t, *J* = 5.9 Hz, 1H), 5.16 (dd, *J* = 22.5, 14.3 Hz, 4H), 4.72 – 4.65 (m, 3H), 4.65 (d, *J* = 14.6 Hz, 1H), 4.63 (s, 3H), 4.56 (q, *J* = 4.3 Hz, 5H), 4.18 (q, *J* = 9.8 Hz, 2H), 3.89 (t, *J* = 5.4 Hz, 4H), 3.78 (s, 5H), 3.73 – 3.66 (m, 3H), 3.64 – 3.50 (m, 20H), 3.31 (td, *J* = 11.7, 5.1 Hz, 2H), 3.26 – 3.18 (m, 2H), 1.91 (s, 4H), 1.47 (s, 31H), 1.31 – 1.24 (m, 8H), 0.88 (t, *J* = 7.0 Hz, 1H), 0.87 (s, 0H). $[M+2H]^{2+}$ = 1343.51, $[M+3H]^{3+}$ = 896.01, $[M+4H]^{4+}$ = 672.26.

Compounds I-10a.

The raw material Propargyloxy-PEG$_2$-COOH (2-(2-(2-(Propargyloxy) ethoxy) ethoxy) acetic acid, 14.68 mg, 0.168 mmol) is sequentially added to 3 mL of anhydrous DMF along with 1-(3-dimethylaminopropyl)-3-ethylcarbodiimide (EDCI, 28.31 mg, 0.145 mmol), HOBt (19.6 mg, 0.145 mmol), DMAP (2.96 mg, 0.024 mmol), and DIPEA (31.27 mg, 0.242 mmol). The mixture is stirred under argon protection at room temperature for 30 minutes, then compound I-**9a** (80 mg, 0.048 mmol) is added. The reaction is continued under argon protection and stirred at room temperature. After 12 h, the product is purified by column chromatography to yield 60 mg, with a 63 % yield. $[M+H]^+$ = 1984.6.

Compounds I-10b.

The starting material Propargyloxy-PEG$_2$-COOH (2-(2-(2-(Propargyloxy) ethoxy) ethoxy) acetic acid, 9.83 mg, 0.0486 mmol) is added to 3 mL of anhydrous DMF along with EDCI (18.96 mg, 0.0972 mmol), HOBT (13.127 mg, 0.0972 mmol), DMAP (1.979 mg, 0.0162 mmol), and DIPEA (20.94 mg, 0.1621 mmol) in sequence. The mixture is stirred under argon protection at room temperature for 30 minutes, then compound I-**9b** (80 mg, 0.03 mmol) is added. The reaction is continued under argon protection and stirred at room temperature. After 12 h, the product is purified by column

chromatography to yield 82 mg. 95.3 % yield. ^{1}H NMR (600 MHz, Chloroform-*d*) δ 11.38 (s, 1H), 9.11 (s, 2H), 8.45 (d, *J* = 8.3 Hz, 2H), 7.83 (dd, *J* = 8.2, 1.2 Hz, 2H), 7.79 (d, *J* = 4.8 Hz, 1H), 7.67 (dt, *J* = 8.3, 1.1 Hz, 1H), 7.37 (dddd, *J* = 20.9, 8.1, 6.8, 1.1 Hz, 3H), 6.89 (d, *J* = 12.8 Hz, 2H), 5.93 (d, *J* = 2.4 Hz, 2H), 5.66 – 5.62 (m, 2H), 5.58 – 5.56 (m, 1H), 5.21 (d, *J* = 9.1 Hz, 2H), 5.06 – 4.99 (m, 2H), 4.69 – 4.61 (m, 4H), 4.59 – 4.53 (m, 9H), 4.50 – 4.46 (m, 2H), 4.18 (d, *J* = 2.4 Hz, 1H), 4.14 (d, *J* = 9.8 Hz, 1H), 3.91 – 3.86 (m, 6H), 3.79 (d, *J* = 9.7 Hz, 11H), 3.68 – 3.66 (m, 2H), 3.65 – 3.63 (m, 3H), 3.60 (q, *J* = 5.6, 4.9 Hz, 21H), 3.53 (dd, *J* = 6.3, 4.0 Hz, 3H), 3.42 (p, *J* = 6.5 Hz, 4H), 3.34 (ddd, *J* = 10.9, 7.0, 4.5 Hz, 3H), 3.30 – 3.22 (m, 2H), 1.91 (s, 6H), 1.48 – 1.45 (m, 54H), 1.43 (d, *J* = 5.9 Hz, 3H), 1.37 (s, 1H), 1.33 (s, 1H), 1.29 (d, *J* = 1.0 Hz, 4H), 1.27 (s, 1H), 1.25 (s, 13H), 0.88 (t, *J* = 7.0 Hz, 4H), 0.86 – 0.82 (m, 2H). $[M+3H]^{3+}$=957.42, $[M+4H]^{4+}$ = 718.32

Compounds I-11a (Z2).

Compound I-10a (60 mg, 0.03 mmol) is dissolved in 2 mL of anhydrous DMF, followed by the addition of 2 mL of TFA. The mixture is stirred at room temperature for 4 h, then TFA is removed using a rotary evaporator. The product is purified by C18 reversed-phase chromatography, yielding 46 mg with a 97 % yield. The product (46 mg, 0.029 mmol) is dissolved in 1.5 mL of methanol, to which 200 µl of double-distilled water is added. The pH is adjusted to 12-13 with a 10 M NaOH solution, and the mixture is stirred at room temperature for 4 h. The pH of the reaction system is then adjusted to 4 using a 4 M hydrochloric acid solution in 1,4-dioxane. Finally, the product is purified by C18 reversed-phase chromatography, yielding 40 mg with a 91.7 % yield. ^{1}H NMR (600 MHz, Deuterium Oxide) δ 7.91 (s, 2H), 5.47 (d, *J* = 2.3 Hz, 2H), 4.48 (d, *J* = 3.4 Hz, 9H), 4.29 (dd, *J* = 9.3, 2.4 Hz, 2H), 4.22 (t, *J* = 11.6 Hz, 4H), 4.06 (t, *J* = 2.6 Hz, 4H), 4.02 (dd, *J* = 11.7, 5.0 Hz, 2H), 3.98 (d, *J* = 2.4 Hz, 3H), 3.84 – 3.79 (m, 8H), 3.60 (d, *J* = 2.9 Hz, 7H), 3.59 – 3.55 (m, 6H), 3.52 (h, *J* = 4.4, 3.5 Hz, 8H), 3.47 (tt, *J* = 7.5, 6.1, 4.7 Hz, 16H), 3.43 (d, *J* = 4.4 Hz, 11H), 3.17 (q, *J* = 8.1, 5.3 Hz, 5H), 2.74 (t, *J* = 2.3 Hz, 1H), 1.88 (s, 6H). ^{13}C NMR (201 MHz, D_2O) δ 174.30, 171.95, 169.00, 156.99, 149.15, 143.83, 125.50, 103.81, 79.39, 76.03, 75.99, 75.15, 70.30, 69.68, 69.56, 69.49, 69.44, 69.38, 69.25, 68.75, 68.59, 63.46, 59.48, 58.46, 57.88, 51.08, 50.06, 47.72, 40.06, 21.97. $[M+H]^{+}$ = 1504.680

Compounds I-11b (Z3).

Compound I-10b (60 mg, 0.028 mmol) is dissolved in 2 mL of anhydrous DMF, followed by the addition of 2 mL of TFA. The mixture is stirred at room temperature for 4 h, then TFA is removed using a rotary evaporator. The product is purified by C18 reversed-phase chromatography, yielding 41 mg with a 64.5 % yield. The product (41 mg, 0.018 mmol) is dissolved in 1.5 mL of methanol, to which 200 µl of double-distilled water is added. The pH is adjusted to 12-13 with a 10 M NaOH solution, and the mixture is stirred at room temperature for 4 h. The pH of the reaction system is then adjusted to 4 using a 4 M hydrochloric acid solution in 1,4-dioxane. Finally, the product is purified

by C18 reversed-phase chromatography, yielding 32 mg with an 82.8 % yield. ^{1}H NMR (600 MHz, Deuterium Oxide) δ 7.89 (s, 4H), 5.48 (dd, *J* = 8.6, 2.3 Hz, 3H), 4.46 (t, *J* = 5.0 Hz, 7H), 4.44 (s, 6H), 4.32 – 4.18 (m, 8H), 4.10 – 4.01 (m, 7H), 4.01 – 3.88 (m, 5H), 3.80 (dd, *J* = 9.9, 4.9 Hz, 13H), 3.67 (dd, *J* = 12.7, 4.6 Hz, 1H), 3.58 (d, *J* = 6.9 Hz, 8H), 3.57 – 3.51 (m, 5H), 3.46 (dddt, *J* = 24.0, 14.0, 6.6, 2.9 Hz, 51H), 3.36 – 3.31 (m, 1H), 3.15 (dq, *J* = 10.5, 5.6 Hz, 7H), 3.09 – 3.03 (m, 1H), 1.87 (s, 7H), 1.81 (d, *J* = 3.5 Hz, 3H). ^{13}C NMR (201 MHz, D_2O) δ 174.30, 171.84, 168.61, 158.46, 156.99, 148.79, 143.90, 125.41, 104.26, 79.40, 76.00, 75.22, 70.30, 70.27, 69.66, 69.55, 69.47, 69.41, 69.37, 69.24, 68.73, 68.56, 63.53, 59.63, 57.86, 51.05, 50.03, 40.05, 21.97. $[M+2H]^{2+}$ = 1075.49, $[M+3H]^{3+}$ = 717.01

Compounds II-4.

Compound II-3 (60 mg, 0.0848 mmol) was dissolved in anhydrous DCM (5 mL). To this solution were added sequentially 2-(2-(2-(2-azidoethoxy)ethoxy)ethoxy)ethanamine (N_3–PEG_3–NH_2, 27.78 mg, 0.127 mmol), HOBT (11.47 mg, 0.0848 mmol), and DIPEA (21.94 mg, 0.1697 mmol). The reaction mixture was stirred at room temperature under a nitrogen atmosphere for 8 h. Upon completion, the crude mixture was purified by column chromatography to afford the product (62 mg) in 93.0% yield. $[M+H]^+$ = 787.95.

Compounds II-5.

Compound II-4 (300 mg, 0.382 mmol) and compound B4 (28.06 mg, 0.119 mmol) were separately dissolved in 1.5 mL of THF. The two solutions were combined and stirred. An aqueous solution of $CuSO_4 \cdot 5H_2O$ (14.29 mg, 0.057 mmol) and NaVC (22.68 mg, 0.115 mmol) was added sequentially to the reaction mixture. The reaction was allowed to proceed at room temperature for 3 h. Upon completion, the mixture was extracted with DCM. The organic layer was purified by column chromatography to afford the product (190 mg) in 57.5 % yield. ^{1}H NMR (600 MHz, Chloroform-*d*) δ 11.43 (s, 3H), 8.56 (d, *J* = 7.6 Hz, 3H), 8.21 (d, *J* = 10.2 Hz, 2H), 7.86 (s, 2H), 5.91 (t, *J* = 5.8 Hz, 2H), 5.16 (dd, *J* = 6.9, 2.9 Hz, 3H), 4.63 (s, 4H), 4.55 (t, *J* = 5.1 Hz, 6H), 4.42 (p, *J* = 7.9 Hz, 3H), 4.22 (ddd, *J* = 11.1, 7.3, 4.3 Hz, 3H), 3.89 (t, *J* = 5.2 Hz, 6H), 3.73 (s, 9H), 3.61 (d, *J* = 7.0 Hz, 27H), 3.54 (dp, *J* = 8.1, 4.5 Hz, 6H), 3.33 (q, *J* = 5.7 Hz, 6H), 2.97 (td, *J* = 7.7, 2.9 Hz, 3H), 2.49 (dt, *J* = 13.4, 8.1 Hz, 3H), 2.35 (td, *J* = 7.6, 4.4 Hz, 3H), 2.06 (s, 8H), 1.82 (dt, *J* = 14.1, 7.4 Hz, 3H), 1.49 (d, *J* = 16.1 Hz, 53H), 1.43 (s, 2H), 1.41 – 1.37 (m, 4H), 1.29 (d, *J* = 3.0 Hz, 3H), 1.26 (s, 8H), 1.14 (dt, *J* = 14.3, 7.3 Hz, 3H), 1.05 (dt, *J* = 7.5, 3.7 Hz, 3H), 0.84 (dt, *J* = 19.0, 7.4 Hz, 20H). $[M+H]^+$ = 2596.4876, $[M+2H]^{2+}$ = 1298.2681, $[M+3H]^{3+}$ = 866.1784

Compounds II-6b

The starting material 2-(2-(2-(prop-2-yn-1-yloxy)ethoxy)ethoxy)acetic acid (18.72 mg, 0.093 mmol), EDCI (35.452 mg, 0.185 mmol), HOBT (24.98 mg, 0.185 mmol), DMAP (3.768 mg, 0.032 mmol), and DIPEA (39.8 mg, 0.308 mmol) were successively dissolved in 3 mL of anhydrous DMF. The reaction mixture was stirred at room temperature under an argon atmosphere for 0.5 h. Subsequently, compound II-5 (160 mg, 0.062 mmol) was added, and the reaction was continued under argon at room temperature for 12 h. After the reaction was complete, the crude product was purified by column chromatography to afford the compound II-6 (126 mg) in 73.1 % yield. ^{1}H NMR (600 MHz, Chloroform-*d*) δ 11.43 (s, 2H), 8.56 (d, *J* = 7.6 Hz, 3H), 8.21 (d, *J* = 10.2 Hz, 2H), 7.72 (d, *J* = 9.0 Hz, 2H), 7.38 – 7.34 (m, 1H), 7.33 – 7.30 (m, 1H), 5.81 – 5.78 (m, 2H), 5.30 (s, 3H), 5.17 – 5.14 (m, 3H), 4.58 (s, 4H), 4.55 (t, *J* = 5.3 Hz, 4H), 4.42 (p, *J* = 7.9 Hz, 3H), 4.22 (ddd, *J* = 11.2, 7.5, 4.4 Hz, 2H), 4.17 (d, *J* = 2.4 Hz, 1H), 3.89 (dd, *J* = 9.1, 3.9 Hz, 6H), 3.81 (s, 4H), 3.73 (s, 6H), 3.61 (d, *J* = 6.4 Hz, 20H), 3.54 – 3.51 (m, 4H), 3.41 (p, *J* = 6.6 Hz, 2H), 3.33 (tt, *J* = 8.5, 3.9 Hz, 6H), 2.97 (td, *J* = 7.9, 2.8 Hz, 3H), 2.51 – 2.45 (m, 3H), 2.35 (td, *J* = 7.6, 4.4 Hz, 2H), 2.31 (t, *J* = 7.5 Hz, 1H), 2.05 (s, 6H), 1.82 (dt, *J* = 14.1, 7.6 Hz, 3H), 1.49 (d, *J* = 16.0 Hz, 54H), 1.43 (s, 3H), 1.37 (s, 3H), 1.29 (s, 5H), 1.26 (s, 18H), 1.13 (dt, *J* = 11.9, 6.1 Hz, 4H), 1.06 – 1.02 (m, 3H), 0.85 (ddt, *J* = 19.9, 14.7, 7.0 Hz, 26H). $[M+H]^+$ = 2780.5539, $[M+2H]^{2+}$ = 1390.8079, $[M+3H]^{3+}$ = 927.5346

Compounds II-7 (P3)

Compound II-6 (120 mg, 0.043 mmol) was dissolved in anhydrous DMF (2 mL), followed by the addition of TFA (2 mL). The reaction mixture was stirred at room temperature for 4 h. Upon completion, TFA was removed using a rotary evaporator. The crude product was purified by C18 reverse-phase chromatography to afford the intermediate (81 mg) in 86.5 % yield. Intermediate (80 mg, 0.036 mmol) was dissolved in MeOH (1.5 mL), followed by the addition of deionized water (200 μL). The pH of the solution was adjusted to 12–13 using 10 N aqueous NaOH. The reaction mixture was stirred at room temperature for 4 h. Subsequently, the pH was adjusted to 4 with a solution of 4 N HCl in dioxane. The mixture was purified by C18 reverse-phase chromatography to afford the product (36 mg) in a yield of 46.8 %. ^{1}H NMR (600 MHz, DMSO-d_6) δ 8.97 (s, 1H), 8.01 (s, 3H), 7.67 (s, 2H), 7.06 (s, 2H), 6.76 (s, 2H), 5.22 – 5.18 (m, 3H), 4.51 (t, *J* = 5.3 Hz, 6H), 4.48 (s, 5H), 4.25 (t, *J* = 10.2 Hz, 4H), 3.84 – 3.77 (m, 8H), 3.66 (d, *J* = 13.7 Hz, 8H), 3.58 – 3.00 (m, 79H), 1.81 (s, 7H), 1.58 (d, *J*

= 12.5 Hz, 4H), 1.30 – 1.23 (m, 4H), 1.08 (s, 3H), 1.00 – 0.93 (m, 4H), 0.85 (dt, *J* = 15.4, 7.2 Hz, 18H). ^{13}C NMR (201 MHz, D_2O) δ 179.22, 173.79, 171.79, 157.21, 155.73, 143.88, 125.34, 77.95, 76.00, 70.30, 69.69, 69.66, 69.52, 69.49, 69.43, 69.41, 69.36, 68.75, 68.56, 67.66, 63.59, 59.62, 55.22, 50.25, 50.05, 49.42, 48.62, 44.14, 40.00, 34.50, 21.86, 21.04, 11.72. $[M+2H]^{2+}$ = 1069.6397, $[M+3H]^{3+}$ = 713.4728

Compounds III-2.

Azido-dipolyethylene glycol-propanoic acid (1.084 g, 5.34 mmol), EDCI (1.53 g, 8.01 mmol), HOBT (1.81 g, 8.01 mmol), and DIPEA (1.378 g, 10.68 mmol) were successively added to 10 mL of anhydrous DMF. The reaction system was stirred at room temperature under an argon atmosphere for 0.5 h. Subsequently, compound III-1 (2 g, 6.4 mmol) was added, and the reaction was continued under argon protection with stirring at room temperature for 12 h. After the reaction was complete, the mixture was purified by column chromatography to afford the product (1.76 g) in a yield of 60.65 %. ^{1}H NMR (600 MHz, DMSO-d_6) δ 7.80 (d, *J* = 9.3 Hz, 1H), 7.70 (d, *J* = 9.0 Hz, 1H), 6.64 (t, *J* = 2.6 Hz, 1H), 4.20 – 4.13 (m, 1H), 4.13 – 4.06 (m, 2H), 3.92 (tdd, *J* = 10.5, 8.9, 5.4 Hz, 1H), 3.75 (dt, *J* = 11.2, 8.9 Hz, 1H), 3.63 – 3.52 (m, 6H), 3.50 (dd, *J* = 5.5, 3.3 Hz, 2H), 3.43 – 3.37 (m, 3H), 3.32 (s, 1H), 2.48 (d, *J* = 5.4 Hz, 1H), 2.30 (d, *J* = 7.7 Hz, 1H), 2.29 – 2.23 (m, 1H), 2.19 (ddt, *J* = 17.1, 10.2, 3.1 Hz, 1H), 1.78 (s, 3H), 1.45 (ddd, *J* = 14.0, 7.4, 5.2 Hz, 1H), 1.43 – 1.38 (m, 2H), 1.38 – 1.32 (m, 1H), 1.22 (t, *J* = 7.1 Hz, 3H), 0.84 (t, *J* = 7.4 Hz, 3H), 0.77 (t, *J* = 7.4 Hz, 3H). $[M+H]^{+}$ = 498.27

Compounds III-3

Compound III-2 (1.76 g, 3.541 mmol) and compound B4 (250 mg, 1.063 mmol) were separately dissolved in 1.5 mL of THF. The solutions were combined and stirred. An aqueous solution of $CuSO_4 \cdot 5H_2O$ (132.66 mg, 0.53 mmol) and Na·VC (210.60 mg, 1.06 mmol) was added sequentially to the reaction mixture. The reaction was carried out at room temperature for 3 h. Upon completion, the mixture was extracted with DCM. The organic layer was purified by column chromatography to afford the product (1.28 g) in a yield of 62.8 %. ^{1}H NMR (600 MHz, DMSO-d_6) δ 8.05 (s, 3H), 7.81 (d, *J* = 9.3 Hz, 3H), 7.71 (d, *J* = 8.9 Hz, 3H), 6.64 (t, *J* = 2.5 Hz, 3H), 4.51 (q, *J* = 3.8, 2.4 Hz, 11H), 4.19 – 4.06 (m, 9H), 3.92 (tdd, *J* = 10.5, 9.0, 5.5 Hz, 3H), 3.81 (t, *J* = 5.3 Hz, 6H), 3.75 (dt, *J* = 11.2, 8.9 Hz, 3H), 3.62 – 3.27 (m, 32H), 2.48 (d, *J* = 5.4 Hz, 2H), 2.32 – 2.22 (m, 6H), 2.22 – 2.15 (m, 3H), 1.77 (s, 8H), 1.50 – 1.31 (m, 13H), 1.21 (t, *J* = 7.1 Hz, 10H), 0.83 (t, *J* = 7.4 Hz, 9H), 0.76 (t, *J* = 7.4 Hz, 9H). $[M+2H]^{2+}$ = 875.45

Compounds III-4.

The starting material 2-(2-(2-(prop-2-yn-1-yloxy)ethoxy)ethoxy)acetic acid (57.99

mg, 0.2868 mmol), EDCI (111.85 mg, 0.5736 mmol), HOBT (77.436 mg, 0.5736 mmol), DMAP (11.68 mg, 0.0956 mmol), and DIPEA (123.55 mg, 0.956 mmol) were successively dissolved in 3 mL of anhydrous DMF. The reaction mixture was stirred at room temperature under an argon atmosphere for 0.5 h. Subsequently, compound III-3 (330 mg, 0.1912 mmol) was added, and the reaction was continued under argon protection with stirring at room temperature for 12 h. After completion of the reaction, the crude product was purified by column chromatography to afford the compound III-4 (305 mg) in a yield of 89.72 %. ^{1}H NMR (600 MHz, DMSO-d_6) δ 8.01 (s, 3H), 7.79 (d, J = 9.3 Hz, 3H), 7.69 (dd, J = 8.8, 3.5 Hz, 3H), 6.63 (d, J = 2.6 Hz, 3H), 4.55 – 4.46 (m, 11H), 4.19 – 4.06 (m, 11H), 3.96 – 3.87 (m, 3H), 3.80 (dd, J = 10.7, 5.4 Hz, 8H), 3.74 (dt, J = 11.3, 8.9 Hz, 3H), 3.66 (s, 4H), 3.58 – 3.36 (m, 30H), 2.47 (d, J = 5.5 Hz, 2H), 2.31 – 2.14 (m, 9H), 1.76 (s, 8H), 1.44 (ddd, J = 14.6, 7.4, 2.2 Hz, 5H), 1.42 – 1.36 (m, 6H), 1.36 – 1.31 (m, 3H), 1.27 – 1.16 (m, 14H), 0.83 (t, J = 7.4 Hz, 10H), 0.76 (t, J = 7.4 Hz, 9H). $[M+2Na]^{2+}$=978.98

Compounds III-5 (O3).

Compound III-4 (150 mg, 0.078 mmol) was completely dissolved in MeOH (1 mL). A 5% aqueous NaOH solution was added dropwise, during which precipitate formed and subsequently dissolved, resulting in a clear solution. The reaction was continued for 1.5 h. After completion, the pH was adjusted to 4 using 1 M HCl under an ice-bath condition. The mixture was extracted with n-butanol. The organic layer was purified by column chromatography to afford the product (95 mg) in a yield of 71.89 %. ^{1}H NMR (600 MHz, DMSO-d_6) δ 8.01 (s, 3H), 7.78 (d, J = 9.2 Hz, 4H), 7.68 (d, J = 8.9 Hz, 4H), 6.60 (d, J = 2.7 Hz, 3H), 4.51 (t, J = 5.3 Hz, 6H), 4.49 (s, 6H), 4.13 (d, J = 2.4 Hz, 2H), 4.09 – 4.05 (m, 4H), 3.94 – 3.87 (m, 4H), 3.81 (t, J = 5.3 Hz, 6H), 3.79 (s, 2H), 3.73 (dt, J = 11.3, 9.0 Hz, 5H), 3.67 (s, 5H), 3.59 – 3.35 (m, 36H), 2.27 (hept, J = 7.6, 7.1 Hz, 8H), 2.15 (ddt, J = 17.0, 10.5, 3.0 Hz, 4H), 1.50 – 1.31 (m, 15H), 0.84 (t, J = 7.4 Hz, 9H), 0.77 (t, J = 7.4 Hz, 9H). ^{13}C NMR (201 MHz, MeOD) δ 172.37, 172.29, 170.81, 168.05, 144.25, 137.62, 129.33, 124.45, 82.37, 79.37, 75.79, 74.76, 70.56, 70.01, 69.93, 69.91, 69.87, 69.03, 68.71, 68.07, 66.80, 63.91, 59.64, 57.65, 54.38, 50.02, 36.41, 25.91, 25.41, 21.66, 8.50, 8.29. $[M+2H]^{2+}$ = 912.9729

AVCs were synthesized with following procedures:

Conjugate Fc-N_3

The Fc protein was exchanged into a PBS solution, and a 5 mg/mL N_3-PEG$_4$-TFP (customized by Hunan Huateng Pharmaceutical Co., Ltd.) stock solution in DMSO was prepared. Take 5 mg of the Fc protein and add N_3-PEG$_4$-TFP (0.278 mg, 0.633 μmol), react overnight at room temperature, where the Fc protein is coupled through its N atom. The product was purified by a PD-10 desalting column chromatography and then concentrated by ultrafiltration, resulting in 4.6 mg of Fc-PEG$_4$-N_3.

Conjugate AVC

Then, **compound I-7 (Z1), I-7-S (Z1-S), I-11a (Z2), I-11b (Z3), II-7 (P3) or III-5 (O3)** (20 eq) was added to **Conjugates Fc-N_3** (1 eq), followed by the sequential addition of $CuSO_4 \cdot 5H_2O$ (0.95 mg, 3.81 μmol), THPTA (1.66 mg, 3.81 μmol), Na·VC (2.7mg, 13.6μmol). The reaction proceeds at room temperature for 4 h. After purification using a PD-10 desalting column chromatography and subsequent ultrafiltration and concentration, the conjugate **AVC-Z1, AVC-Z1H, AVC-Z1H-S, AVC-Z2**, **AVCZ3, AVC-P3C** or **AVC-O3** were obtained. CD388 used for comparison in this study was synthesized in-house following the procedure described by Döhrmann et al.[1].

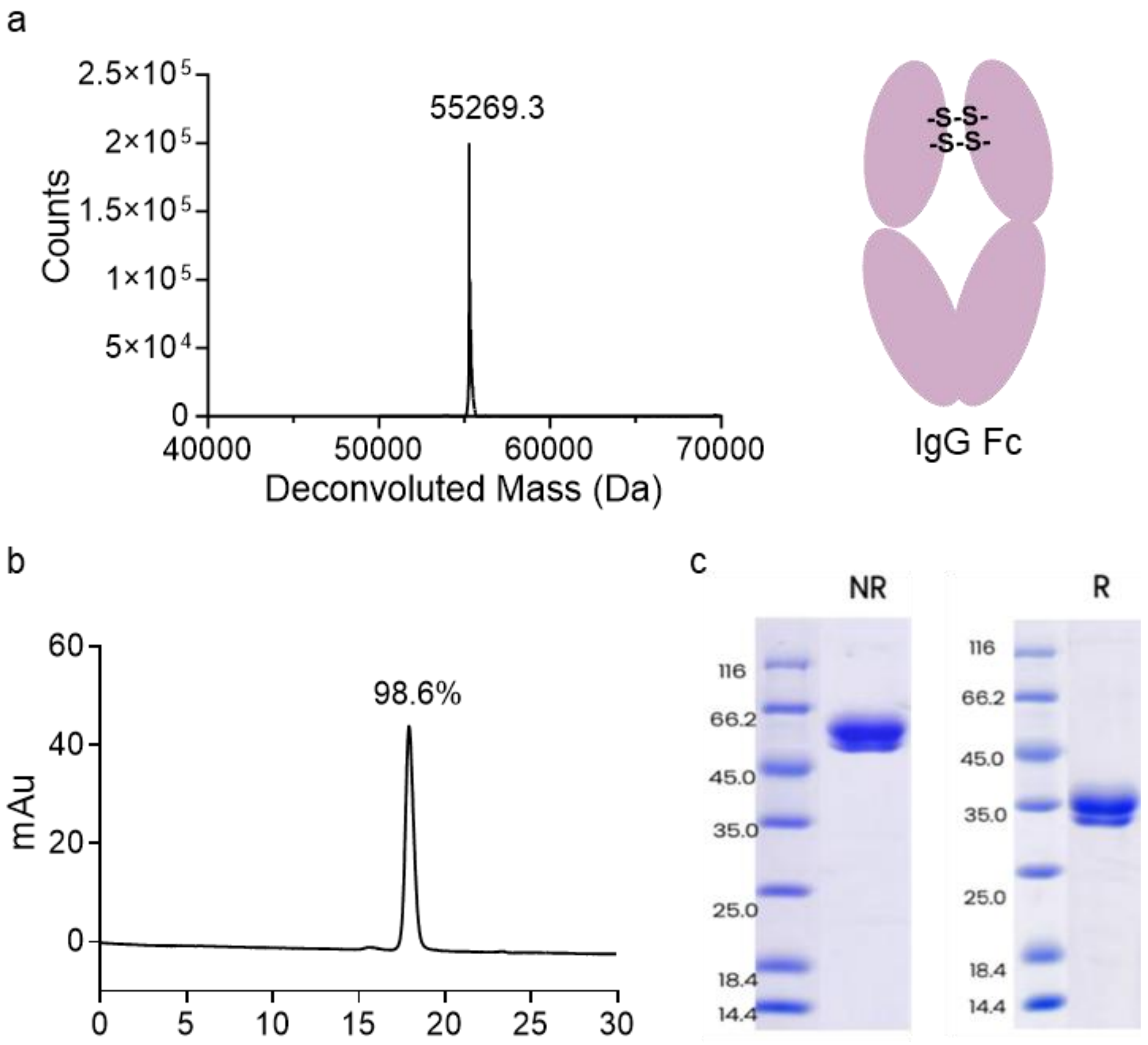

Supplementary Figure 8. Characterizations of Fc. a. UPLC-Q-TOF-MS analysis of Fc. **b.** Size-exclusion chromatography analysis of Fc. **c.** Immunoblot analysis of Fc.

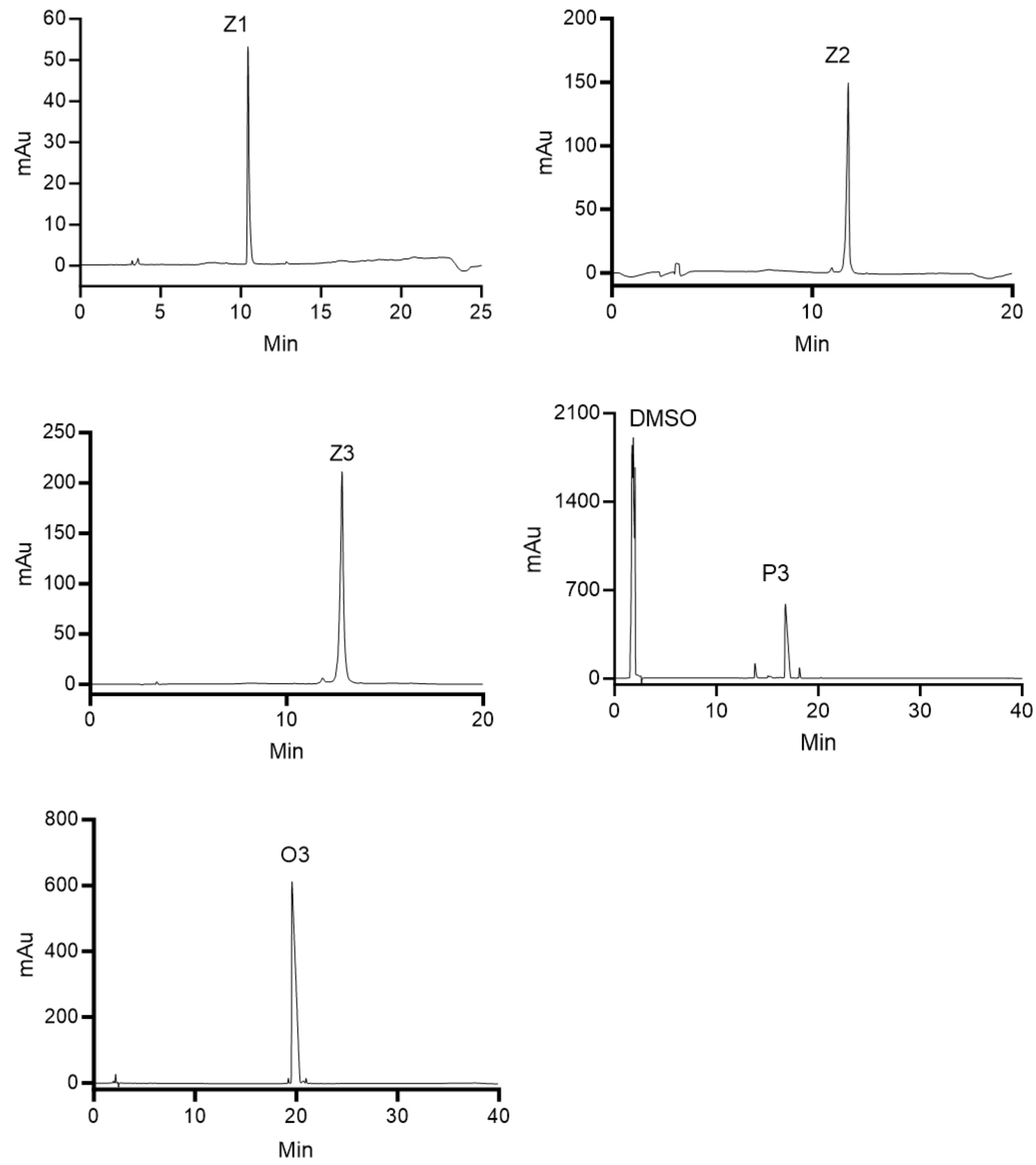

Supplementary Figure 9. Purity analysis of Z1, Z2, Z3, P3 and O3. Z1, Z2, Z3 dissolving in water and P3, O3 dissolving in DMSO were analyzed by high-performance liquid chromatography.

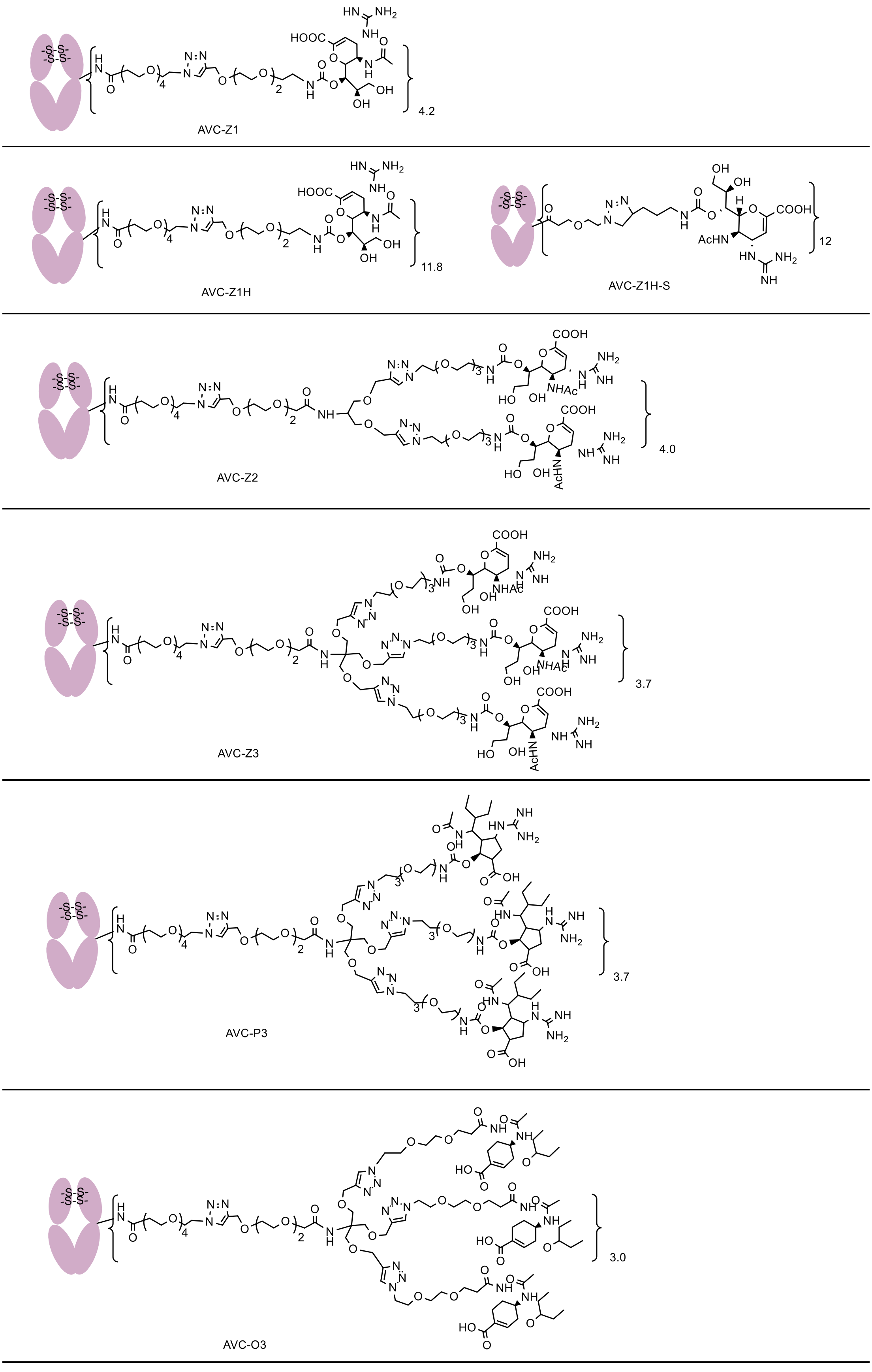

Supplementary Figure 10. Structures of AVCs. The structures and DFRs of AVC-Z1, AVC-Z1H-S, AVC-Z2, AVC-Z1H, AVC-Z3, AVC-P3, AVC-O3 were shown.

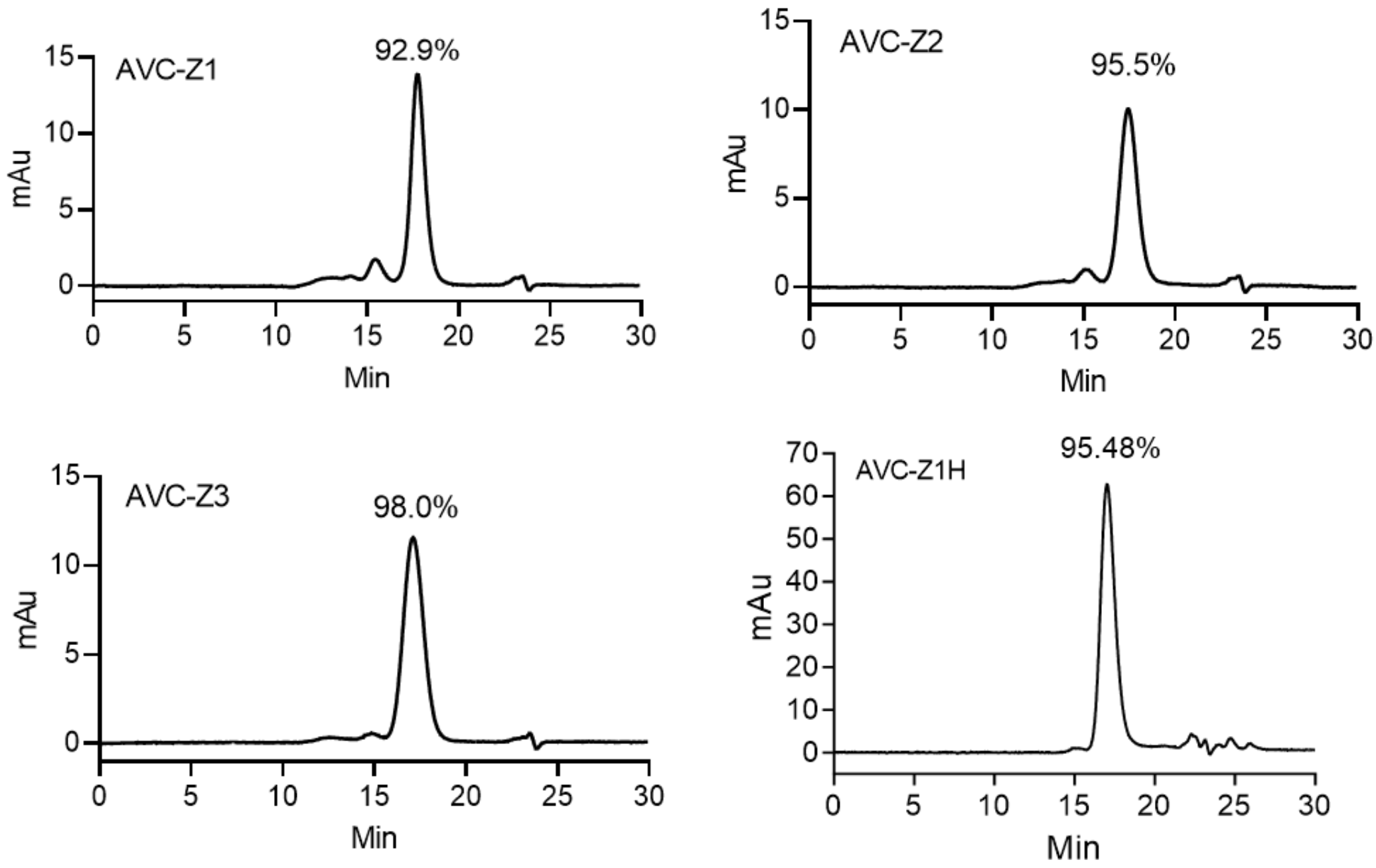

Supplementary Figure 11. Size-exclusion chromatography analysis of AVCs. The monomer contents of AVC-Z1, AVC-Z2, AVC-Z1H and AVC-Z3 exceed 90%.

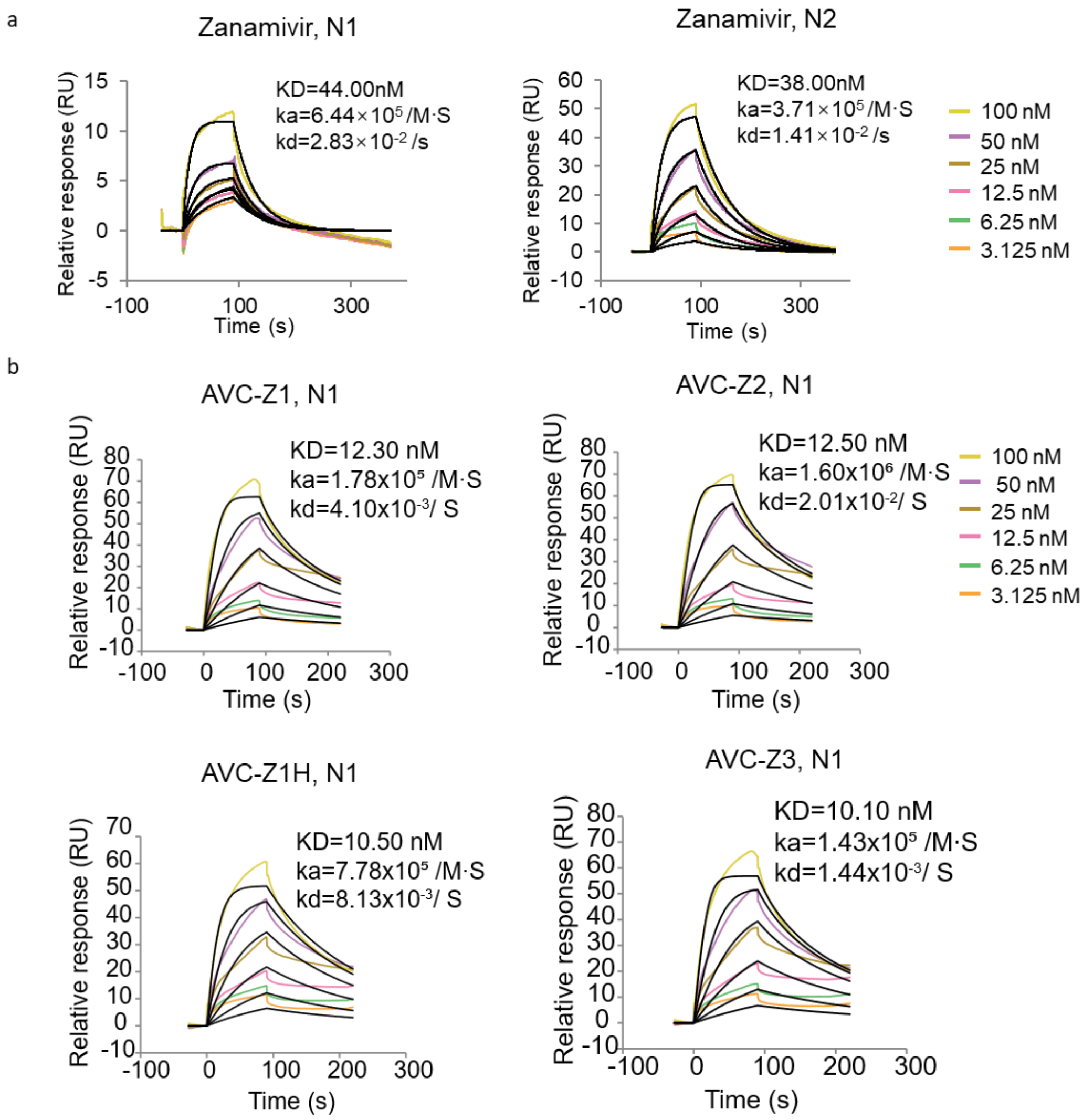

Supplementary Figure 12. SPR analysis of zanamivir and AVCs. AVCs binding with NA (N1) revealed that AVC-Z1, AVC-Z2, AVC-Z1H, and AVC-Z3 exhibited progressively higher affinities for NA. SPR results are representative of three independent experiments.

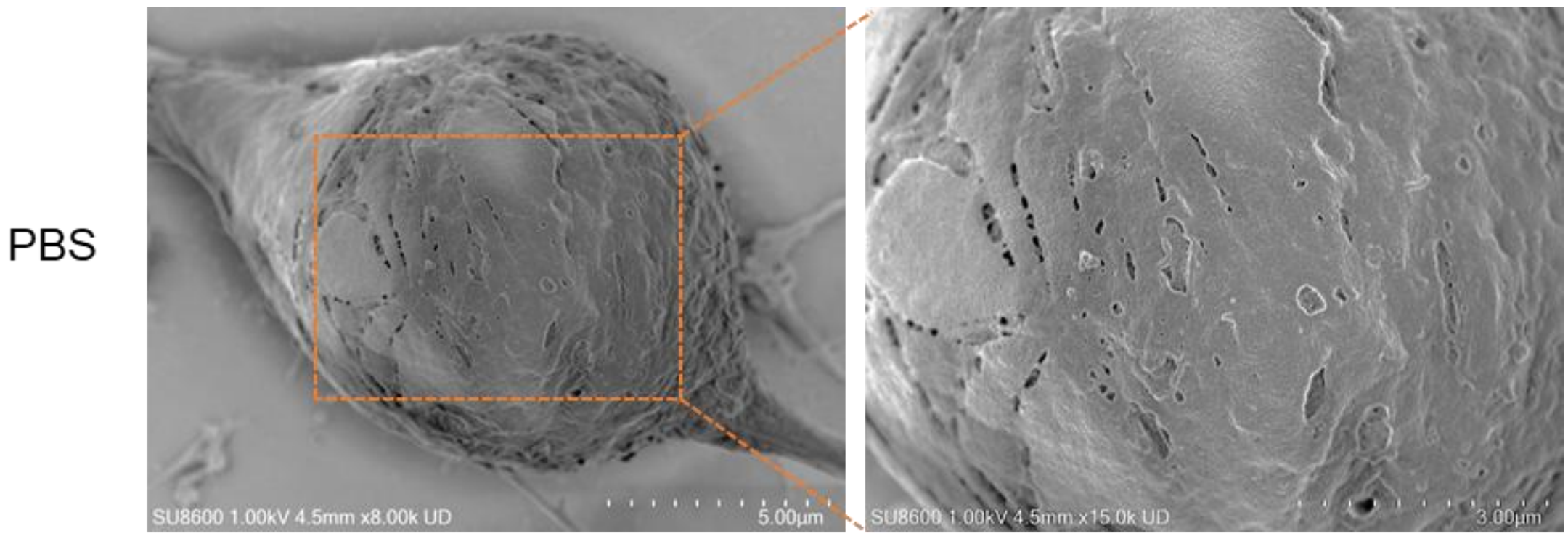

Supplementary Figure 13. TEM micrographs of cell. Cells without virus were used as control in virus cross-linking experiment.

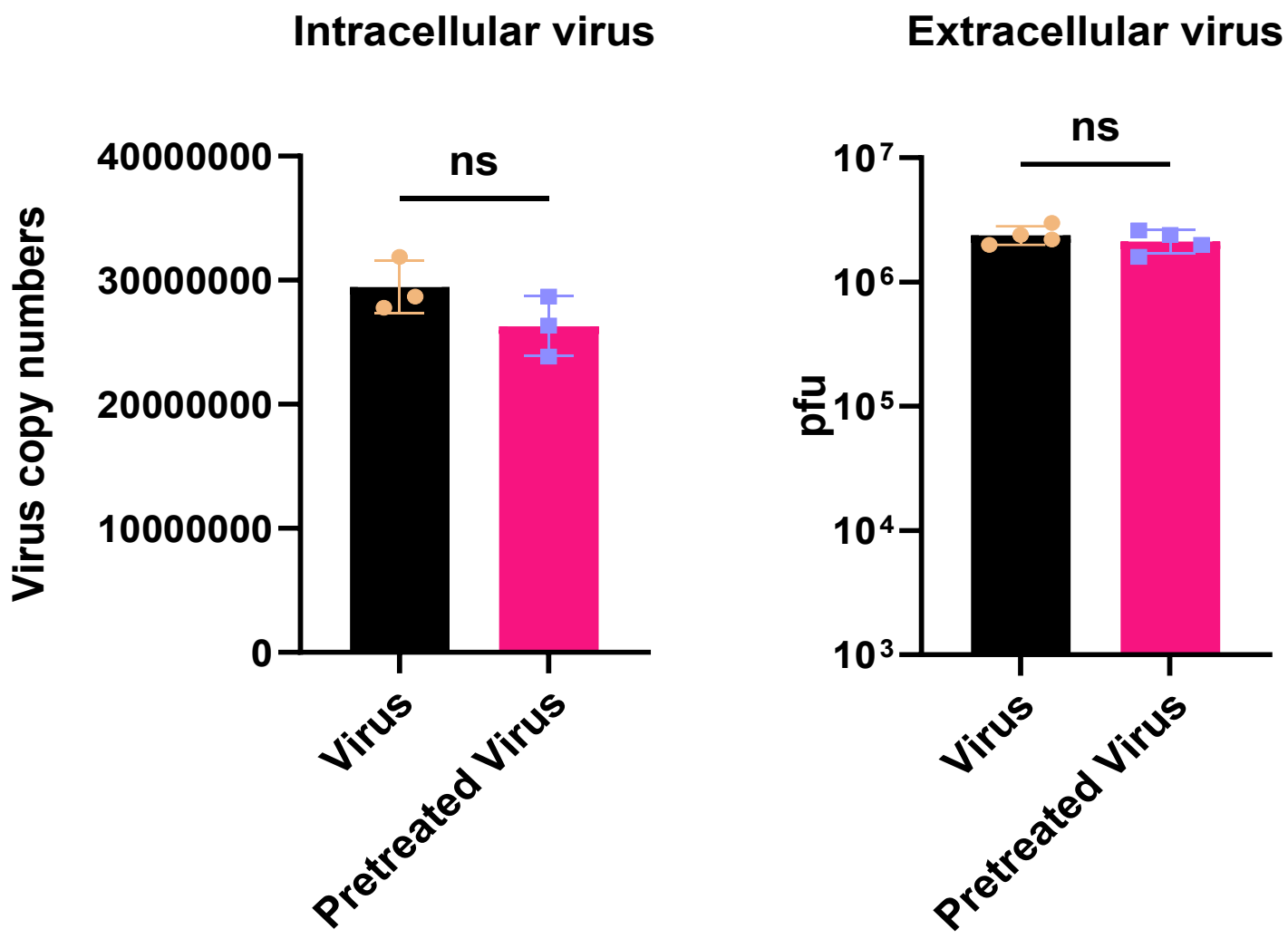

Supplementary Figure 14. The entry blockade assays of AVC-Z1H-S with short linker. Then negative control AVC-Z1H-S also containing the bulky Fc groups did not block the viral entry process. Intracellular (n = 3 independent experiments, P = 0.1673, ns) and extracellular (n = 4 independent experiments, P = 0.4502, ns) data are representative of two and three independent experiments. Data are shown as mean ± s.d. P values were calculated using unpaired two-tailed t-test. Source data are provided as a Source Data file.

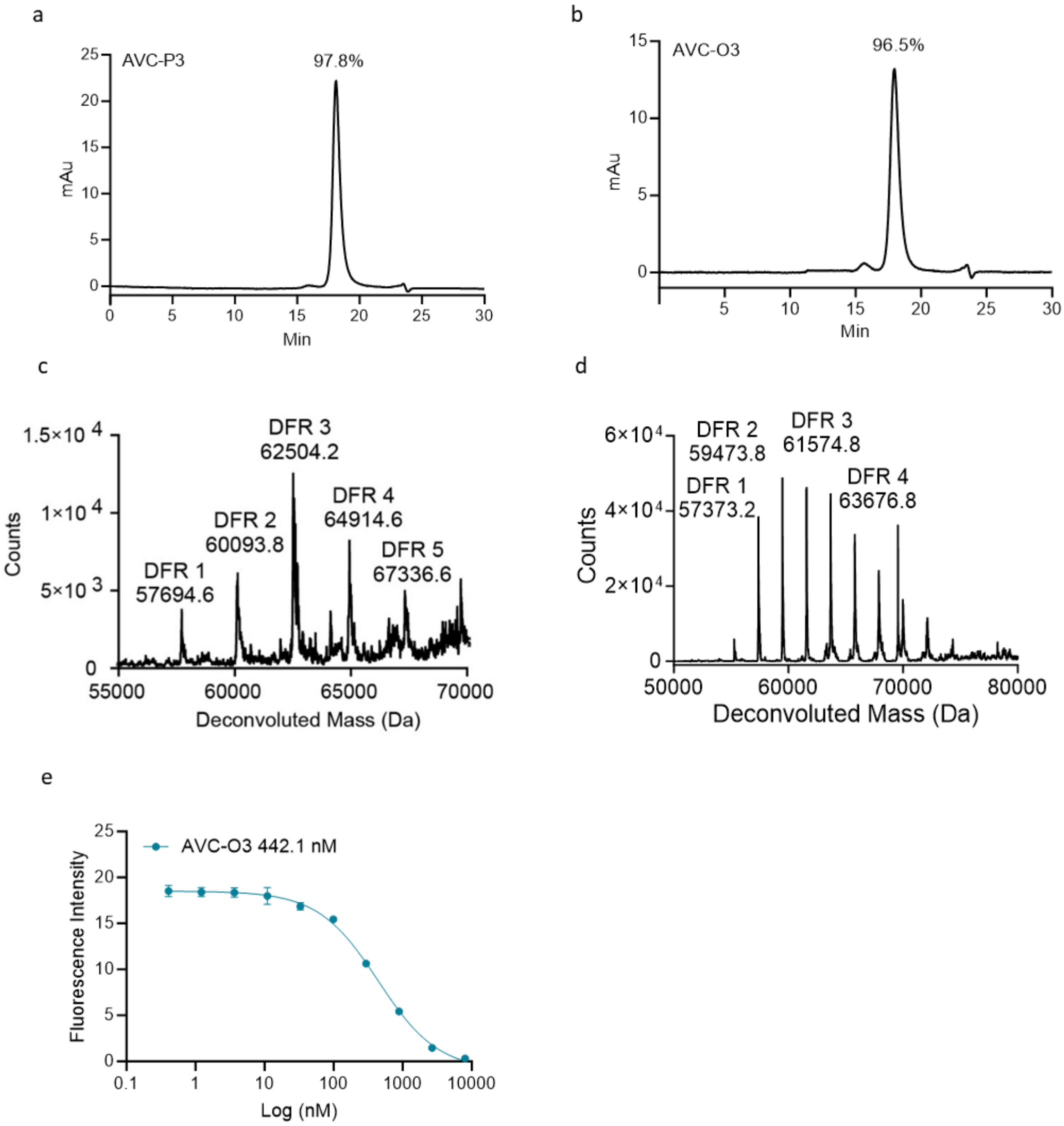

Supplementary Figure 15. Characterization of AVCs. a. SEC analysis of AVC-P2, AVC-P3. **b.** SEC analysis of AVC-O3. **c.** UPLC-Q-TOF-MS analysis of AVC-P3. **d** UPLC-Q-TOF-MS analysis of AVC-O3. **e.** NA inhibition analysis of AVC-O3. NA inhibition data are representative of three independent experiments. Data are shown as mean ± s.d. Source data are provided as a Source Data file.

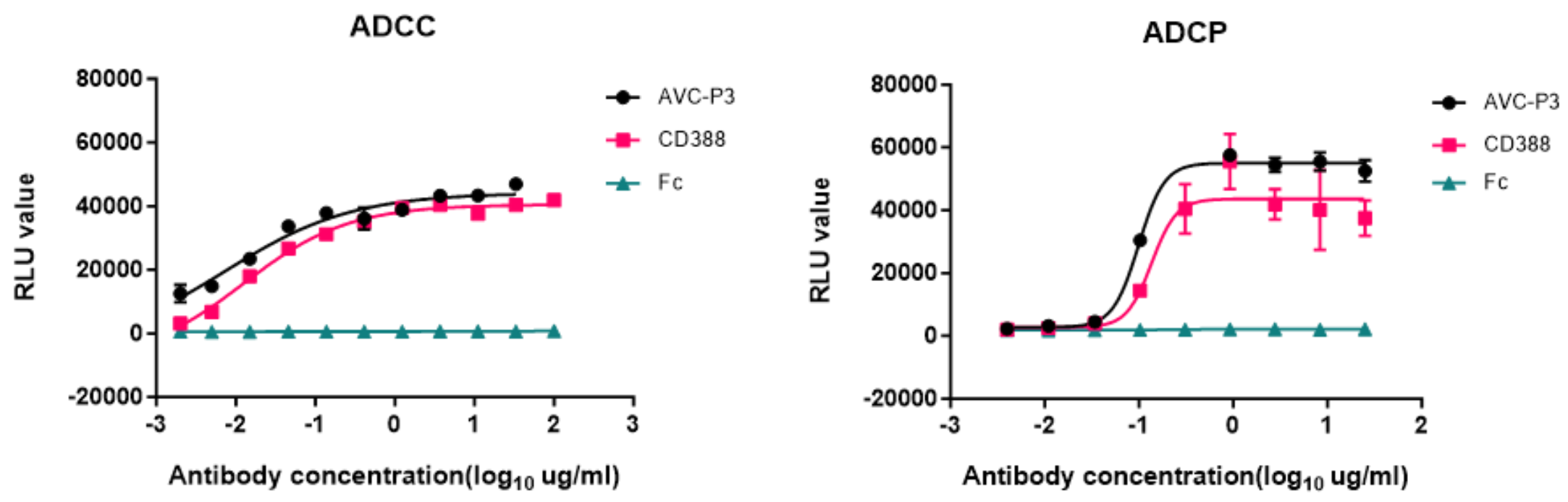

Supplementary Figure 16. ADCC, ADCP effects analysis of AVC-P3, CD388 and Fc. ADCC and ADCP effects analysis of AVC-P3, CD388 and Fc using Jurkat-Lucia™ NFAT-CD16/ Jurkat-Lucia™ NFAT-CD32 cells. Data are representative of two independent experiments. RLU: relative light unit. Data are shown as mean ± s.d. Source data are provided as a Source Data file.

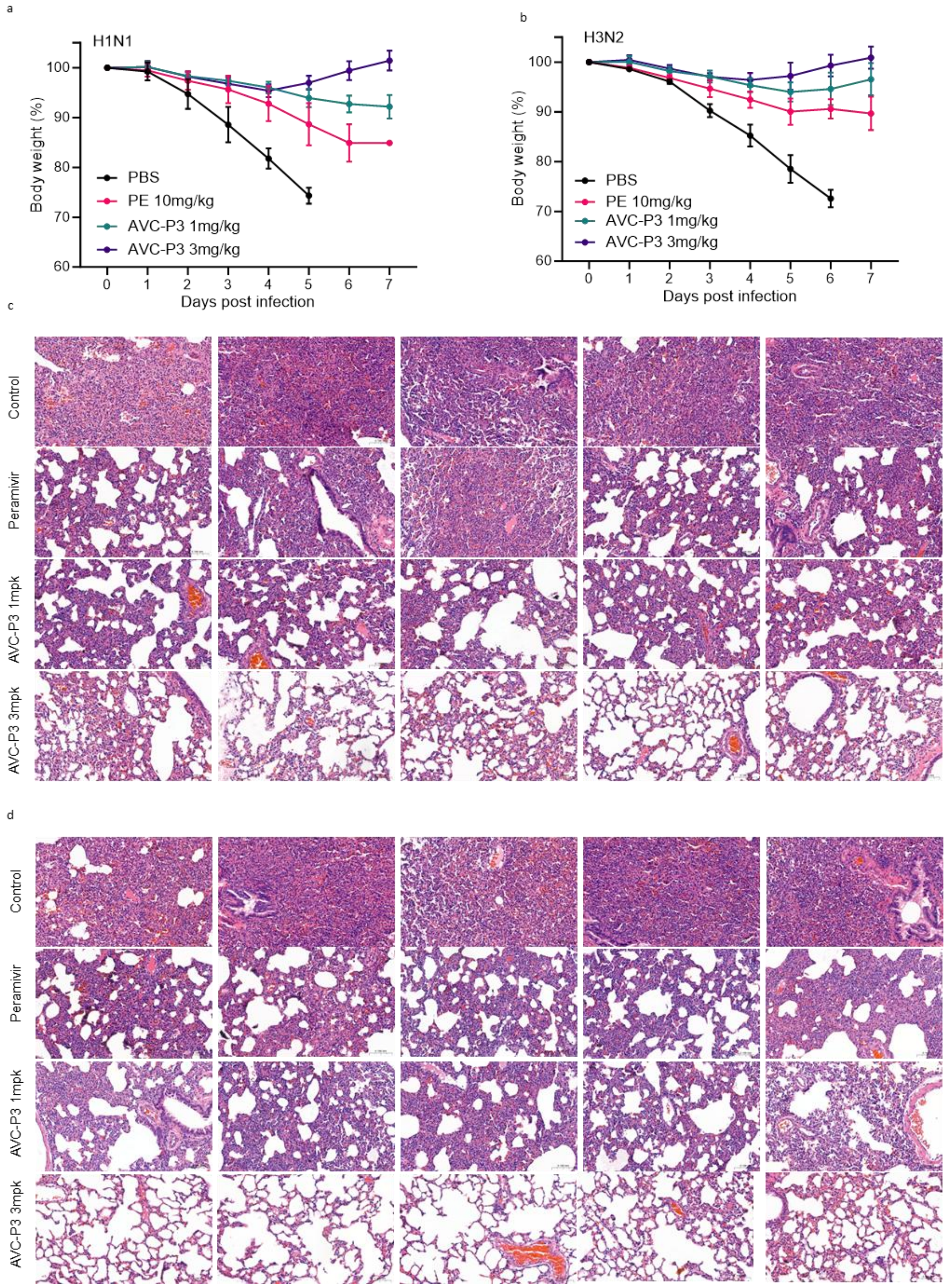

Supplementary Figure 17. Treatment efficacy of AVC-P3 in lethal mouse models of H1N1 and H3N2 infection. a. %body weight change of AVC-P3 in the H1N1 infection model (*n* = 5 mice per group). **b.** %body weight change of AVC-P3 in the H3N2 infection model (*n* = 5 mice per group). **c.** Hematoxylin and eosin staining of lung tissue in the H1N1 infection model treatment with AVC–P3 (*n* = 5 mice per group).

d. Hematoxylin and eosin staining of lung tissue in the H3N2 infection model treatment with AVC–P3 ($n = 5$ mice per group). Source data are provided as a Source Data file.

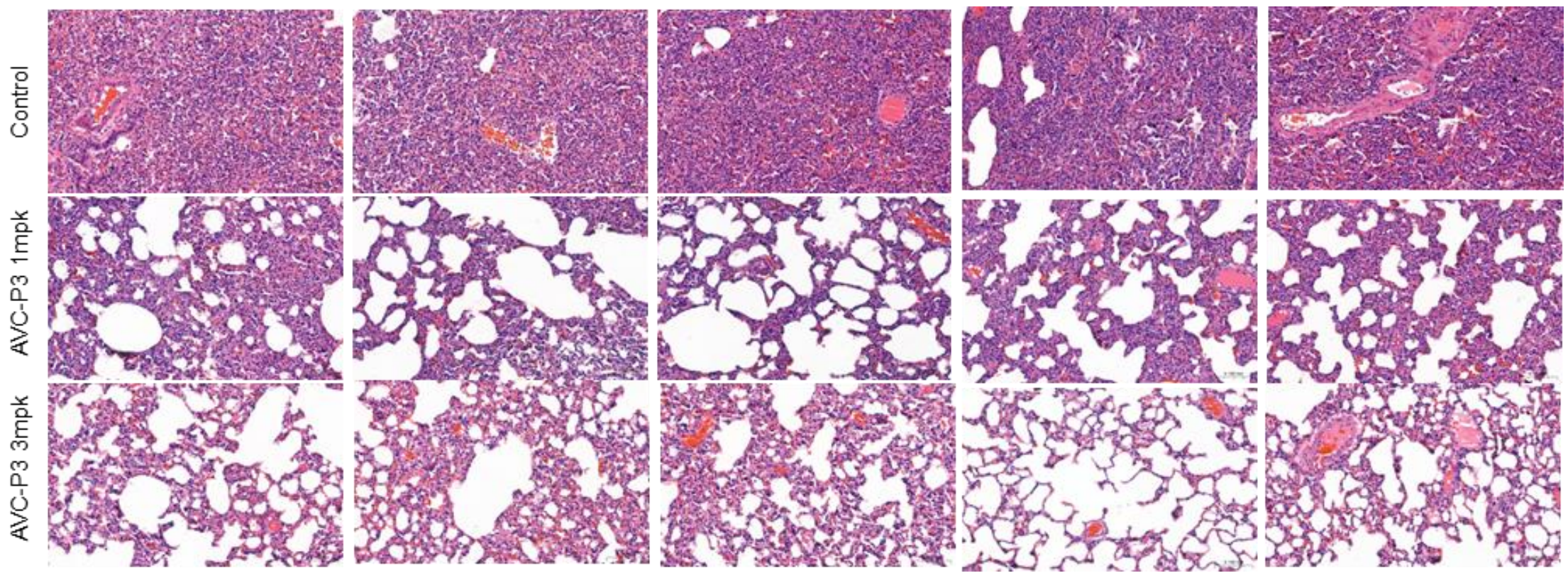

Supplementary Figure 18. Hematoxylin and eosin staining of lung tissue in AVC-3 pretreatment group. Representative H&E stainin of AVC-P3 pretreatment group (1 mg/kg and 3mg/kg, $n = 5$ mice per group).

Supplementary Table 1 The antivirus activities of oseltamivir, AVC-O3 and short linker control AVC-Z1H-S.

Compounds	EC_{50} (nM)						
	A/Puerto Rico/8/1934 (H1N1)	A/Victoria/2570/2009 (H1N1)	A/California/04/2009 (H1N1)	A/Vietnam/2004 (H5N1)	A/Shanghai/02/2013 (H7N9)	B/Florida/04/2006 (BY)	B/Phuket/3073/2013 (BY)
oseltamivir	/	125	29.62	266.67	100.02	100.03	> 2000
AVC-O3	/	> 2000	> 600	> 600	> 600	> 600	> 2000
AVC-Z1H-S	12.84±1.91	/	31.79±2.68	/	/	/	/

Supplementary Table 2 Comprehensive linker information of different AVCs

Comp.	Conjugation sites	No. of atoms (between NAI monomers)	Distance (between NAI monomers)	No. of atoms (between NAI and Fc)	Distance (between NAI and Fc)
AVC-Z1H	Lysine residue of Fc	/	/	33	～36Å
AVC-Z1H-S	Lysine residue of Fc	/	/	15	～18Å
AVC-Z3	Lysine residue of Fc	39	～37Å	33	～36Å
AVC-P3	Lysine residue of Fc	39	～37Å	33	～36Å
CD388	Lysine residue of Fc	15	～18Å	32	35～40 Å

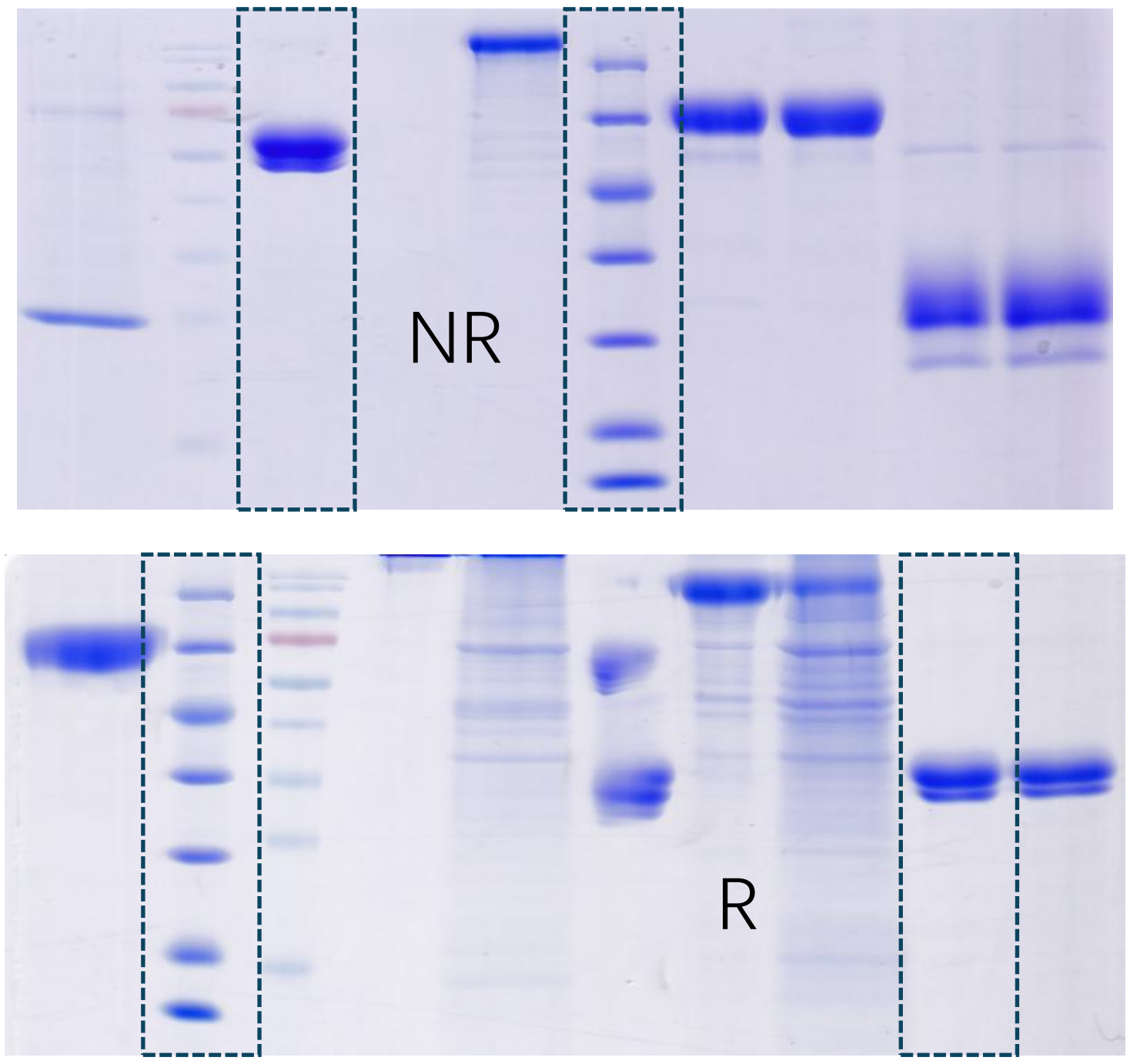

Uncropped scan corresponding to Supplementary Figure 8c

References

1. Döhrmann, S., Levin, J., Cole, J.N. et al. Drug–Fc conjugate CD388 targets influenza virus neuraminidase and is broadly protective in mice. *Nat Microbiol* **10**, 912–926 (2025).
