# Supplementary material for: Multivalent antiviral conjugates enhance activity against Influenza via cross-linking viruses
Source: Nat Commun. 2026 Oct 4;17:11320. doi: 10.1038/s41467-026-78126-3 (PMC13724737; doi:10.1038/s41467-026-78126-3)
Supplement: Supplementary file 2 [file 41467_2026_78126_MOESM2_ESM.pdf]

nature portfolio

Corresponding author(s): Xinbo Zhou

Last updated by author(s): Aug 25, 2026

Reporting Summary

Nature Portfolio wishes to improve the reproducibility of the work that we publish. This form provides structure for consistency and transparency in reporting. For further information on Nature Portfolio policies, see our Editorial Policies and the Editorial Policy Checklist.

Statistics

For all statistical analyses, confirm that the following items are present in the figure legend, table legend, main text, or Methods section.

n/a	Confirmed	
☐	☒	The exact sample size (*n*) for each experimental group/condition, given as a discrete number and unit of measurement
☐	☒	A statement on whether measurements were taken from distinct samples or whether the same sample was measured repeatedly
☐	☒	The statistical test(s) used AND whether they are one- or two-sided<br>*Only common tests should be described solely by name; describe more complex techniques in the Methods section.*
☒	☐	A description of all covariates tested
☐	☒	A description of any assumptions or corrections, such as tests of normality and adjustment for multiple comparisons
☐	☒	A full description of the statistical parameters including central tendency (e.g. means) or other basic estimates (e.g. regression coefficient) AND variation (e.g. standard deviation) or associated estimates of uncertainty (e.g. confidence intervals)
☐	☒	For null hypothesis testing, the test statistic (e.g. F, t, r) with confidence intervals, effect sizes, degrees of freedom and P value noted<br>*Give P values as exact values whenever suitable.*
☒	☐	For Bayesian analysis, information on the choice of priors and Markov chain Monte Carlo settings
☒	☐	For hierarchical and complex designs, identification of the appropriate level for tests and full reporting of outcomes
☒	☐	Estimates of effect sizes (e.g. Cohen's d, Pearson's r), indicating how they were calculated

Our web collection on statistics for biologists contains articles on many of the points above.

Software and code

Policy information about availability of computer code

Data collection	A NanoDropOne spectrophotometer (Thermo Fisher Scientific) was used to measure the UV-vis spectra to quantify AVC concentrations. UPLC-Q-TOF-MS (Waters, USA) was used to measure DFRs. An Agilent 1260 HPLC system (Agilent Technologies, USA) was used for size-exclusion chromatography (SEC) analysis. The Biacore 8K instrument (Cytiva, USA) was use for Binding affinity assay. A SpectraMax Gemini EM Microplate Reader (Molecular Devices, USA) was used to monitor the neuraminidase (NA) inhibition activity IC50. An EnVision Multimode Plate Reader (PerkinElmer, USA) was employed to measure luminescence intensity for the evaluation of in vitro cytotoxicity EC50. A DynaPro NanoStar II system (Wyatt Technology, USA) was employed to measure the size distribution of the complexes via dynamic light scattering (DLS). A JEM-1400 Plus transmission electron microscope (JEOL, Japan) was employed to determine the particle size of the virus-AVC complexes. A Quanta 200 scanning electron microscope (FEI Company, The Netherlands) was used to visualize the virus particles on the surface of infected cells. A Leica DMI8 inverted fluorescence microscope (Leica Microsystems, Germany) was used to capture fluorescence images of the infected cells.
Data analysis	GraphPad Prism (10.2) was used for data analysis and curve-fitting. The Biacore 8K Insight Evaluation Software (Cytiva, version 5.0.18.22102) was utilized for automated data processing and curve fitting to determine kinetic parameters.

For manuscripts utilizing custom algorithms or software that are central to the research but not yet described in published literature, software must be made available to editors and reviewers. We strongly encourage code deposition in a community repository (e.g. GitHub). See the Nature Portfolio guidelines for submitting code & software for further information.

Data

Policy information about availability of data

All manuscripts must include a data availability statement. This statement should provide the following information, where applicable:

- Accession codes, unique identifiers, or web links for publicly available datasets
- A description of any restrictions on data availability
- For clinical datasets or third party data, please ensure that the statement adheres to our policy

All data generated in this study are provided within the paper and its Supplementary Information. Source Data are provided with this paper.

Research involving human participants, their data, or biological material

Policy information about studies with human participants or human data. See also policy information about sex, gender (identity/presentation), and sexual orientation and race, ethnicity and racism.

Reporting on sex and gender	n/a
Reporting on race, ethnicity, or other socially relevant groupings	n/a
Population characteristics	n/a
Recruitment	n/a
Ethics oversight	n/a

Note that full information on the approval of the study protocol must also be provided in the manuscript.

Field-specific reporting

Please select the one below that is the best fit for your research. If you are not sure, read the appropriate sections before making your selection.

☒ Life sciences ☐ Behavioural & social sciences ☐ Ecological, evolutionary & environmental sciences

For a reference copy of the document with all sections, see nature.com/documents/nr-reporting-summary-flat.pdf

Life sciences study design

All studies must disclose on these points even when the disclosure is negative.

Sample size	No sample size calculation was performed. The choice of sample size was guided by extensive laboratory experience and is consistent with established protocols in the field. For biochemical and biophysical characterizations, such as SPR affinity analysis, DLS, and electron microscopy, measurements were performed in at least triplicate to ensure data reproducibility. In vitro functional assays (e.g., cell viability and neuraminidase inhibition) were conducted with at least three independent biological replicates, as these systems typically show low technical variability. For in vivo studies, the number of mice per group was chosen based on our previous experience to provide sufficient power for detecting statistically significant differences in pharmacokinetic and pharmacodynamic parameters while minimizing animal usage.
Data exclusions	No data were excluded
Replication	All in vitro experiments were independently repeated at least three times with biologically independent samples, and all findings were reproducible. All in vivo experiments were performed using biologically independent mice, with group sizes as specified in each figure legend, and results were consistent across replicate experiments.
Randomization	Mice were randomly assigned to experimental groups. For in vitro experiments, group allocation was not relevant.
Blinding	No blinding was performed in vitro and in vivo experiments in order to make comparisons between specific treatments

Reporting for specific materials, systems and methods

We require information from authors about some types of materials, experimental systems and methods used in many studies. Here, indicate whether each material, system or method listed is relevant to your study. If you are not sure if a list item applies to your research, read the appropriate section before selecting a response.

Materials & experimental systems

n/a	Involved in the study
☐	☒ Antibodies
☐	☒ Eukaryotic cell lines
☒	☐ Palaeontology and archaeology
☐	☒ Animals and other organisms
☒	☐ Clinical data
☒	☐ Dual use research of concern
☒	☐ Plants

Methods

n/a	Involved in the study
☒	☐ ChIP-seq
☒	☐ Flow cytometry
☒	☐ MRI-based neuroimaging

Antibodies

Antibodies used	Monoclonal Anti-Human-IgG-Fc Antibody,Mouse IgG1 (clone: 6F11C8) (IGG-S307, ACRO Biosystems) used at 1:500 dilution for Capture.<br>Peroxidase-labeled goat anti-human IgG (Fc) polyclonal antibody (catalog no. 5220-0279, SeraCare), used at 1:1000 dilution for ELISA.
Validation	The antibodies were validated by the suppliers for ELISA. Additionally, the authors validated the antibody by demonstrating a strong linear correlation ($R^2 > 0.999$) with standard human IgG and observing no signal in negative control samples (buffer only and non-target protein wells).

Eukaryotic cell lines

Policy information about cell lines and Sex and Gender in Research

Cell line source(s)	MDCK and A549 cells were obtained from Institute of Basic Medicine, Chinese Academy of Medical Sciences (Beijing, China)
Authentication	Cell lines have not been subjected to additional authentication
Mycoplasma contamination	All cell lines regularly tested negative for mycoplasma infection using the MycoFluor™ Mycoplasma Detection Kit(M7006,Thermo)
Commonly misidentified lines (See ICLAC register)	No commonly misidentified cells were used in this study.

Animals and other research organisms

Policy information about studies involving animals; ARRIVE guidelines recommended for reporting animal research, and Sex and Gender in Research

Laboratory animals	All animal experiments were conducted using 6-to-8-week-old female mice, including C57BL/6J, BALB/c, and DBA/2 strains for pharmacodynamic studies, and BALB/c, C57BL/6J, and humanized FcRn mice (generated on a C57BL/6J background) for pharmacokinetic evaluations. Animals were housed in a specific pathogen-free (SPF) facility under controlled environmental conditions with a constant temperature and humidity, and a 12-hour light/dark cycle. Mice were provided with ad libitum access to a standard rodent diet and water. All procedures were approved by the Institutional Animal Care and Use Committee (IACUC) and performed in accordance with relevant ethical regulations.
Wild animals	No wild animals were involved in this study
Reporting on sex	Only female mice were used, as they are more docile for group housing and handling, and the influenza infection models used in this study are not sex-dependent. Sex was not considered as a variable in the study design.
Field-collected samples	The study did not involve field-collected samples.
Ethics oversight	All animal experiments involving virus infection in this study were approved by the Ethics Committee of the Guangdong–Hong Kong Joint Laboratory of Emerging Infectious Diseases (Approval No. SUMC2022-051) and the WuXi Institutional Animal Care and Use Committee (Approval No. ID01-QD008-2023v1.2). All experiments were performed in accordance with relevant institutional and national guidelines and regulations.

Note that full information on the approval of the study protocol must also be provided in the manuscript.

Plants

Seed stocks	n/a
Novel plant genotypes	n/a
Authentication	n/a
