# Supplementary material for: Multivalent antiviral conjugates enhance activity against Influenza via cross-linking viruses
Source: Nat Commun. 2026 Oct 4;17:11320. doi: 10.1038/s41467-026-78126-3 (PMC13724737; doi:10.1038/s41467-026-78126-3)
Supplement: Supplementary file 3 [file 41467_2026_78126_MOESM3_ESM.pdf]

nature portfolio

Peer Review File

Multivalent anti-viral conjugates enhance activity against Influenza via cross-linking viruses

Corresponding Author: Professor Xinbo Zhou

This file contains all reviewer reports in order by version, followed by all author rebuttals in order by version.

Version 0:

Reviewer comments:

Reviewer #1

(Remarks to the Author)
In this work, the authors developed a programmable multivalent antivirus conjugates (AVCs) platform based on click chemistry, enabling flexible combination of valencies and conjugated small-molecule neuraminidase inhibitors (NAIs). By conjugating mono-, di-, or trivalent NAIs to an antibody Fc fragment, they systematically investigated the impact of valency on antiviral activity. The trivalent zanamivir-Fc conjugate (AVC-Z3) demonstrated enhanced activity by inducing inter-virion cross-linking and viral aggregation, thereby blocking viral entry while restricting viral release, a dual-stage mechanism distinct from conventional neuraminidase inhibition. Further optimization led to AVC-P3 (trivalent peramivir-Fc conjugate), which exhibited superior in vivo half-life and antiviral efficacy compared to AVC-Z3 and the clinical-stage candidate CD388, with potent activity against multiple influenza strains including H1N1, H3N2, H5N1, and influenza B. The modular nature of this platform, combined with the promising preclinical profile of AVC-P3, supports its potential as a next-generation prophylactic and therapeutic agent against influenza. Overall, the study is technically sound and well-executed, and the findings represent a meaningful advance in the field. I believe this work merits publication in Nature Communications pending the following minor revisions to improve clarity and completeness.

1. The discussion section should include a more explicit comparative analysis between AVC-P3 and CD388, particularly regarding their structural design, half-life differences, and in vivo efficacy. The current manuscript touches upon these points but would benefit from a consolidated discussion contextualizing the advantages of the AVC platform relative to this clinical-stage benchmark.

2. The authors propose that inter-virion cross-linking contributes to the inhibition of viral invasion. It would strengthen the mechanistic interpretation to further elaborate on the proposed model, specifically how virion aggregation on the cell surface prevents entry, and whether this effect is dependent on multivalent engagement or influenced by Fc-mediated interactions.

3. In Figure 3, please clarify whether panel c represents an enlarged view of panel b or a distinct experimental condition. Ambiguity in the relationship between subpanels should be resolved.

4. Several figures require attention to detail. For instance, the Y-axis in Figure 6f is incorrectly labeled; fonts and units are inconsistent across figures. A thorough revision is recommended to ensure uniformity and accuracy.

5. Key references on zanamivir conjugates are missing, including prior work from the Hidde L. Ploegh group (PMID: 23185023) and the Jianzhu Chen group (PMID: 41183201). Citing these studies would appropriately contextualize the current work within the broader landscape of Fc-conjugated antiviral strategies.

Reviewer #2

(Remarks to the Author)
This manuscript presents a promising strategy for the design of multivalent antiviral conjugates. By employing the Fc domain as a scaffold to construct a trivalent neuraminidase inhibitor platform, the authors have demonstrated superior antiviral efficacy and broad-spectrum protection against multiple influenza strains through both in vitro and in vivo experiments. This work not only provides potential long-acting drug candidates for clinical translation but also proposes a novel molecular

mechanism of physical intervention (cross-linking), which possesses considerable scientific value and application prospects.
Notably, the design concept of small molecule-macromolecule conjugate antiviral drugs has been previously applied in influenza virus research, and relevant studies can be cited to highlight the comparative advantages of the current work. For instance, Liu X, Balligand T, Le Gall C, Ploegh HL. A monoclonal anti-hemagglutinin stem antibody modified with zanamivir protects against both influenza A and B viruses. Proc Natl Acad Sci U S A. 2025 Apr 15;122(15):e2424889122. This study developed a monoclonal anti-hemagglutinin stem antibody modified with zanamivir, which exhibited protection against both influenza A and B viruses; however, it focused on antibody modification with a single antiviral moiety, lacking the multivalent design and cross-linking mechanism proposed herein. Another study, (Liu X, Balligand T, Carpenet C, Ploegh HL. An armed anti-immunoglobulin light chain nanobody protects mice against influenza A and B infections. Sci Immunol. 2023 Jun 23;8(84):eadg9459.), reported an armed anti-immunoglobulin light chain nanobody for influenza protection, but the nanobody-based scaffold has inherent limitations in valency adjustment and in vivo stability compared to the Fc domain used in this manuscript. In their earlier work (Bioconjug Chem. 2021 Aug 18;32(8):1548-1553), Liu et al. designed neuraminidase-targeted imaging and therapeutic agents, yet these agents were monovalent and failed to achieve the enhanced efficacy derived from multivalency and cross-linking. Additionally, Liu X, et al (Nat Commun. 2020 Nov 5;11(1):5597), proposed a dual-mechanism immunotherapy for influenza, but it relied on a combination of different functional modules rather than the intrinsic cross-linking effect of a single multivalent conjugate. In contrast, the current work innovatively constructs a trivalent platform using the Fc domain as a scaffold, and the unique cross-linking mechanism distinguishes it from the aforementioned studies, endowing it with more potent and broad-spectrum antiviral activity.
While the authors have tentatively demonstrated the contribution of the "cross-linking" mechanism to the enhancement of anti-influenza potency via well-designed experiments, the following refinements in experimental design and presentation are suggested to strengthen the mechanistic arguments and meet the publication standards of high-impact journals:
1. Inclusion of a necessary short-linker control group to identify the primary driver of enhanced antiviral activity (avidity vs. cross-linking). It is recommended to supplement the study with a trivalent conjugate featuring a short linker as a critical negative control. This control group would possess the avidity gain inherent to multivalency but, due to geometric constraints, would be unable to physically bridge separate viral particles. If this control displays significantly lower experimental activity compared to the long-linker groups (such as AVC-Z3), it would definitively confirm that "cross-linking" rather than mere "receptor saturation" is the core mechanism of action for this design.
2. Inclusion of a short-linker control group to determine the root cause of entry blockade. In the entry blockade assays (Figure 3e), a short-linker control group incapable of inducing cross-linking should be included. By comparing the inhibitory effects of AVC-Z3 versus this short-linker counterpart during the viral entry process, the authors can exclude the possibility that the inhibition is simply due to the bulky Fc-conjugate sterically shielding adjacent hemagglutinin (HA) binding sites. This would prove that physical viral aggregation is indeed the primary driver of impaired viral entry.
3. To substantiate the central claim that an optimal linker length triggers effective cross-linking, it is essential for the authors to provide a comprehensive table in the Supporting Information. This table must explicitly detail the physical parameters for each experimental group, including the specific number of PEG units, the exact conjugation sites, and the calculated extended lengths. Such detailed information will help readers better understand the relationship between linker properties and cross-linking efficiency, thereby strengthening the mechanistic rigor of the study.
4. Refinement of the Mechanistic Logic in the Introduction. The authors note in the Abstract and Background that while CD388 has validated the clinical potential of Fc-conjugation, its underlying pharmacodynamic mechanism remains elusive. However, the manuscript currently lacks a clear discussion regarding the structural and mechanistic distinctions or connections between the proposed trivalent platform and CD388. This section should be expanded to better contextualize the current work, highlighting how the trivalent design and cross-linking mechanism address the limitations of CD388 and advance the field of Fc-conjugated antiviral drugs.
By effectively decoupling the contributions of affinity versus cross-linking through short-linker experiments and supplementing the aforementioned details, the authors will significantly strengthen the scientific rigor and impact of the "cross-linking" mechanism for multivalent antibody conjugates. This is a relatively good piece of work, and I recommend publication once these points of evidence are refined.

Version 1:

Reviewer comments:

Reviewer #2

(Remarks to the Author)
This manuscript systematically constructs programmable multivalent antiviral conjugates (AVCs) via click chemistry, and successfully develops trivalent Fc-conjugated AVC-P3 based on peramivir. The study elucidates a novel dual antiviral mechanism: inter-virion cross-linking simultaneously blocks viral entry and progeny release. Comprehensive in vitro assays and multiple mouse infection models (H1N1, H3N2, H5N1, influenza B, severe infection, drug-resistant strains) demonstrate that AVC-P3 exhibits superior efficacy to CD388, baloxavir and free neuraminidase inhibitors, with long systemic circulation and dual prophylactic/therapeutic potency. The authors have fully supplemented, validated and addressed all questions raised by reviewers regarding structural design, antiviral mechanism, pharmacokinetics, in vivo efficacy, control experiments and synthetic characterizations. The revised manuscript features rigorous logic and solid experimental evidence, fully meeting the journal’s publication requirements. I recommend acceptance of this manuscript.

Open Access This Peer Review File is licensed under a Creative Commons Attribution 4.0 International License, which permits use, sharing, adaptation, distribution and reproduction in any medium or format, as long as you give appropriate credit to the original author(s) and the source, provide a link to the Creative Commons license, and indicate if changes were made.
In cases where reviewers are anonymous, credit should be given to 'Anonymous Referee' and the source.
The images or other third party material in this Peer Review File are included in the article’s Creative Commons license, unless indicated otherwise in a credit line to the material. If material is not included in the article’s Creative Commons license and your intended use is not permitted by statutory regulation or exceeds the permitted use, you will need to obtain permission directly from the copyright holder.
To view a copy of this license, visit https://creativecommons.org/licenses/by/4.0/

Point-by-point responses to the reviewers' comments (Manuscript ID: NCOMMS-26-020227-T)

We would like to thank all reviewers for their constructive comments and suggestions on our manuscript. These comments are all valuable and very helpful for revising and improving the quality of our manuscript. We have carefully considered their constructive feedback and conducted several additional investigations to address their concerns and strengthen our findings. Our responses to the reviewers' comments on a point-by-point basis are given here in blue.

Response to Reviewer 1:

In this work, the authors developed a programmable multivalent antivirus conjugates (AVCs) platform based on click chemistry, enabling flexible combination of valencies and conjugated small-molecule neuraminidase inhibitors (NAIs). By conjugating mono-, di-, or trivalent NAIs to an antibody Fc fragment, they systematically investigated the impact of valency on antiviral activity. The trivalent zanamivir-Fc conjugate (AVC-Z3) demonstrated enhanced activity by inducing inter-virion cross-linking and viral aggregation, thereby blocking viral entry while restricting viral release, a dual-stage mechanism distinct from conventional neuraminidase inhibition. Further optimization led to AVC-P3 (trivalent peramivir-Fc conjugate), which exhibited superior in vivo half-life and antiviral efficacy compared to AVC-Z3 and the clinical-stage candidate CD388, with potent

activity against multiple influenza strains including H1N1, H3N2, H5N1, and influenza B. The modular nature of this platform, combined with the promising preclinical profile of AVC-P3, supports its potential as a next-generation prophylactic and therapeutic agent against influenza. Overall, the study is technically sound and well-executed, and the findings represent a meaningful advance in the field. I believe this work merits publication in Nature Communications pending the following minor revisions to improve clarity and completeness.

Response: We thank the reviewer for the positive feedback of our manuscript and appreciate the suggestions for further improving our manuscript. In response to the suggestions, we have added discussions about the comparisons of AVC-P3 and CD388, inter-virion cross-linking mechanism. In addition, we have carefully corrected the errors the figures and updated the references. The detailed responses to the line-by-line comments are provided below.

Point 1. The discussion section should include a more explicit comparative analysis between AVC-P3 and CD388, particularly regarding their structural design, half-life differences, and in vivo efficacy. The current manuscript touches upon these points but would benefit from a consolidated discussion contextualizing the advantages of the AVC platform relative to this clinical-stage benchmark.

Response: Thank you for your professional advice. The comparative analysis between AVC-P3 and CD388, including structural design, half-life differences, and *in vivo* efficacy are as follows. (1) In structural design, AVC-P3 select the neuraminidase (NA) inhibitor peramivir and adopts a trivalent design to conjugate a total of 11 peramivir molecular, facilitating multivalent cross-linking with viruses. In compression, CD388 select the NA inhibitor zanamivir and adopts a bivalent design to conjugate a total of 9 zanamivir molecular. (2) In half-life differences, AVC-P3 exhibits a markedly longer half-life in than CD388 (253 h vs. 163 h), which may confer superior long-term protective efficacy when combined with AVC. (3) In the H1N1 influenza infection model, the median protective dose (ED_{50}) of AVC-P3 is substantially lower than that of CD388. AVC-P3 achieves 50% animal protection even at an ultralow dose of 0.05 mg/kg, whereas CD388 provides no protective effect at the identical dosage. In future clinical translation, AVC-P3 may present prominent advantages over CD388, including stronger antiviral activity and an extended half-life, which translates to a longer duration of protective action. These discussions have been been incorporated into the “Discussion section” (line 464-479 on page 20-21).

Point 2. The authors propose that inter-virion cross-linking contributes to the inhibition of viral invasion. It would strengthen the mechanistic

interpretation to further elaborate on the proposed model, specifically how virion aggregation on the cell surface prevents entry, and whether this effect is dependent on multivalent engagement or influenced by Fc-mediated interactions.

Response: Thank you for your professional advice. Viruses are colloidal particles with limited half-lives and no motility. They must diffuse to host cells before decaying [PMID: 10755888]. Multivalent engagement aggregation by AVC-P3 hinders diffusion and reduces both the surface-to-volume ratio and the number of infectious particles, decreasing adsorption and the fraction of cells reachable. So, this effect is mainly dependent on multivalent engagement. These discussions have been been incorporated into the “Discussion section” (line 485-489 on page 21-22).

Point 3. In Figure 3, please clarify whether panel c represents an enlarged view of panel b or a distinct experimental condition. Ambiguity in the relationship between subpanels should be resolved.

Response: Thank you for your careful work. These are two separate experiments. We have removed the auxiliary dashed lines from the Fig. 3c, Fig. 4e and Fig. 6 for a more tidy and clean presentation of the Figures.

Point 4. Several figures require attention to detail. For instance, the Y-axis in Figure 6f is incorrectly labeled; fonts and units are inconsistent across

figures. A thorough revision is recommended to ensure uniformity and accuracy.

Response: Thank you for your careful work. The labeling of the Y-axis in Fig. 6c, 6f has been unified with that in Figure 6 and the font in the Fig. 6b-6g has been enlarged. In addition, revisions to Figure 6i, 6j were made in accordance with the ethical endpoints.

Point 5. Key references on zanamivir conjugates are missing, including prior work from the Hidde L. Ploegh group (PMID: 23185023) and the Jianzhu Chen group (PMID: 41183201). Citing these studies would appropriately contextualize the current work within the broader landscape of Fc-conjugated antiviral strategies.

Response: Thank you for your careful work. We described more about the zanamivir conjugates in the Introduction section and cited this meaningful paper in the "Introduction section" (line 68-74 on page 3) as following:

Small molecule-macromolecule conjugate antiviral drugs has been also previously reported. Hidde L. Ploegh group and Jianzhu Chen group have developed zanamivir conjugates for influenza protection, focusing on antibody modification with a single antiviral moiety (PMID: 41183201, PMID: 23185023). Philip S. Low group and Hidde L. Ploegh group have reported a dual-mechanism immunotherapy for influenza, recruiting or activating immune effectors (PMID: 37352373, PMID: 33154358).

Response to Reviewer 2:

This manuscript presents a promising strategy for the design of multivalent antiviral conjugates. By employing the Fc domain as a scaffold to construct a trivalent neuraminidase inhibitor platform, the authors have demonstrated superior antiviral efficacy and broad-spectrum protection against multiple influenza strains through both in vitro and in vivo experiments. This work not only provides potential long-acting drug candidates for clinical translation but also proposes a novel molecular mechanism of physical intervention (cross-linking), which possesses considerable scientific value and application prospects.

Notably, the design concept of small molecule-macromolecule conjugate antiviral drugs has been previously applied in influenza virus research, and relevant studies can be cited to highlight the comparative advantages of the current work. For instance, Liu X, Balligand T, Le Gall C, Ploegh HL. A monoclonal anti-hemagglutinin stem antibody modified with zanamivir protects against both influenza A and B viruses. Proc Natl Acad Sci U S A. 2025 Apr 15;122(15):e2424889122. This study developed a monoclonal anti-hemagglutinin stem antibody modified with zanamivir, which exhibited protection against both influenza A and B viruses; however, it focused on antibody modification with a single antiviral moiety, lacking the multivalent design and cross-linking mechanism proposed herein. Another study, (Liu X, Balligand T, Carpenet C, Ploegh HL. An armed anti-

immunoglobulin light chain nanobody protects mice against influenza A and B infections. Sci Immunol. 2023 Jun 23;8(84):eadg9459.), reported an armed anti-immunoglobulin light chain nanobody for influenza protection, but the nanobody-based scaffold has inherent limitations in valency adjustment and in vivo stability compared to the Fc domain used in this manuscript. In their earlier work (Bioconjug Chem. 2021 Aug 18;32(8):1548-1553), Liu et al. designed neuraminidase-targeted imaging and therapeutic agents, yet these agents were monovalent and failed to achieve the enhanced efficacy derived from multivalency and cross-linking. Additionally, Liu X, et al (Nat Commun. 2020 Nov 5;11(1):5597), proposed a dual-mechanism immunotherapy for influenza, but it relied on a combination of different functional modules rather than the intrinsic cross-linking effect of a single multivalent conjugate. In contrast, the current work innovatively constructs a trivalent platform using the Fc domain as a scaffold, and the unique cross-linking mechanism distinguishes it from the studies, endowing it with more potent and broad-spectrum antiviral activity.

While the authors have tentatively demonstrated the contribution of the "cross-linking" mechanism to the enhancement of anti-influenza potency via well-designed experiments, the following refinements in experimental design and presentation are suggested to strengthen the mechanistic arguments and meet the publication standards of high-impact journals:

Response: Thank you for your professional advice. (1) According to your comment, we have described smaller molecule-macromolecule conjugate antiviral drugs (line 68-74 on page 3) and cited these meaningful papers in the introduction section. References 19, 20, 21, 22 have newly added. (2) we have designed the short-chain trivalent AVC variants (PEG1 and PEG0 linker) and the short-chain monovalent AVC variants (AVC-Z1H-S). Then we have performed in vitro antiviral assays and time-course experiments with AVC-Z1H-S to strengthen the cross-linking mechanism. The detailed responses to the line-by-line comments are provided below.

Point 1. Inclusion of a necessary short-linker control group to identify the primary driver of enhanced antiviral activity (avidity vs. cross-linking). It is recommended to supplement the study with a trivalent conjugate featuring a short linker as a critical negative control. This control group would possess the avidity gain inherent to multivalency but, due to geometric constraints, would be unable to physically bridge separate viral particles. If this control displays significantly lower experimental activity compared to the long-linker groups (such as AVC-Z3), it would definitively confirm that "cross-linking" rather than mere "receptor saturation" is the core mechanism of action for this design.

Response: Thank you for your professional advice. According to your advice, we have designed two short-linker control groups, short trivalent

zanamivir conjugates and short monovalent zanamivir conjugate. (1) Firstly, we have designed two trivalent conjugates (079-A7 and 080-A7) featuring short PEG1 and PEG0 linkers. However, during the synthesis, the click reaction failed to yield the desired product under various click reaction conditions (Table R1), but formation of the byproduct A4 was detected. The Scheme (Scheme R1) and LC-MS monitoring data (Figure R1) of the reaction are shown below. Based on the exploratory experiments, it is preliminarily inferred that the trivalent zanamivir with short linkers (PEG1 and PEG0) are likely unstable, resulting in cleavage of the carbamate linkage during the click reaction and formation of byproduct A4.

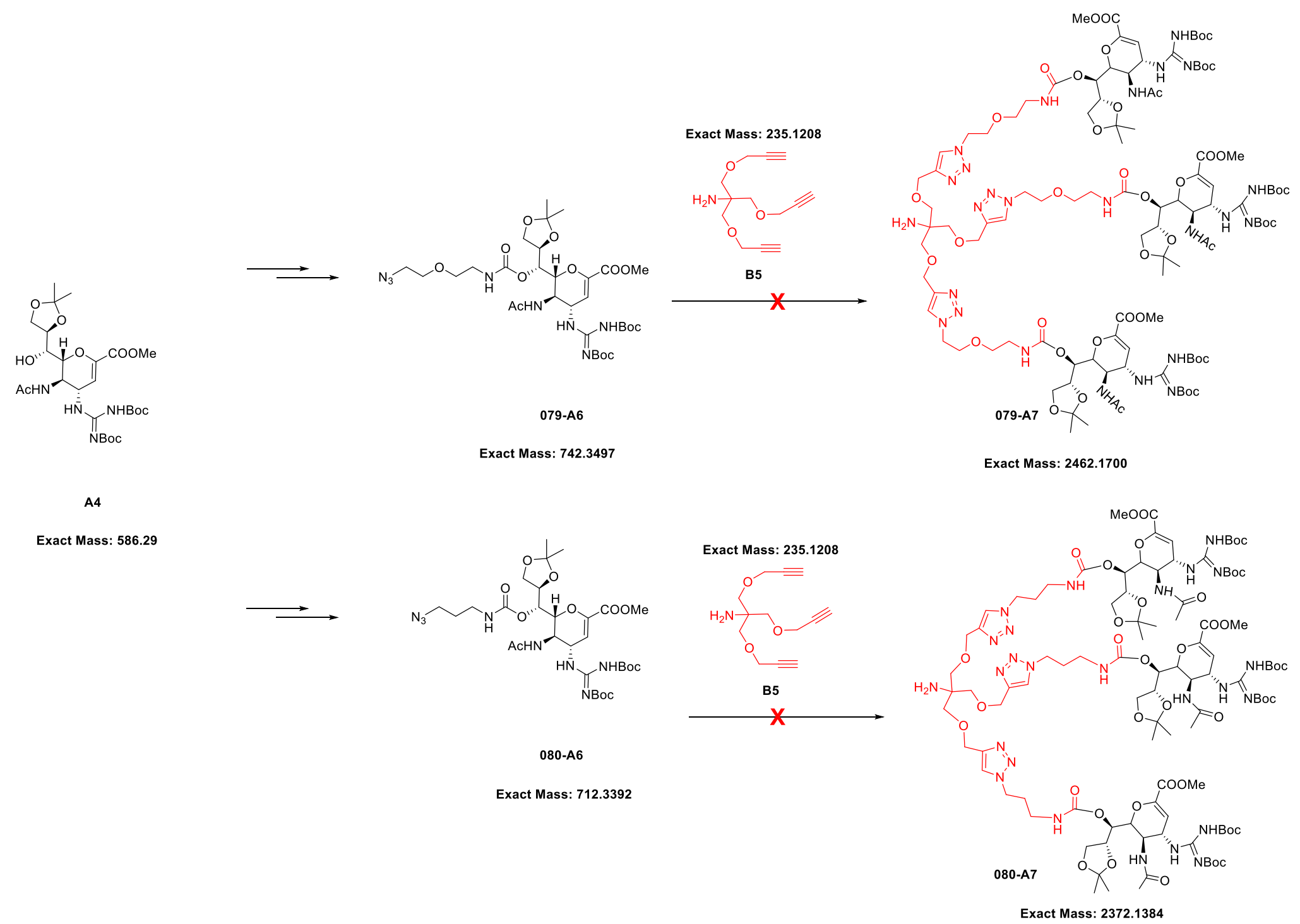

Scheme R1. Unsuccessful synthesis of trivalent zanamivir controls. The click reaction of (079-A7 and 080-A7) with short PEG1 and PEG0 linkers failed to proceed.

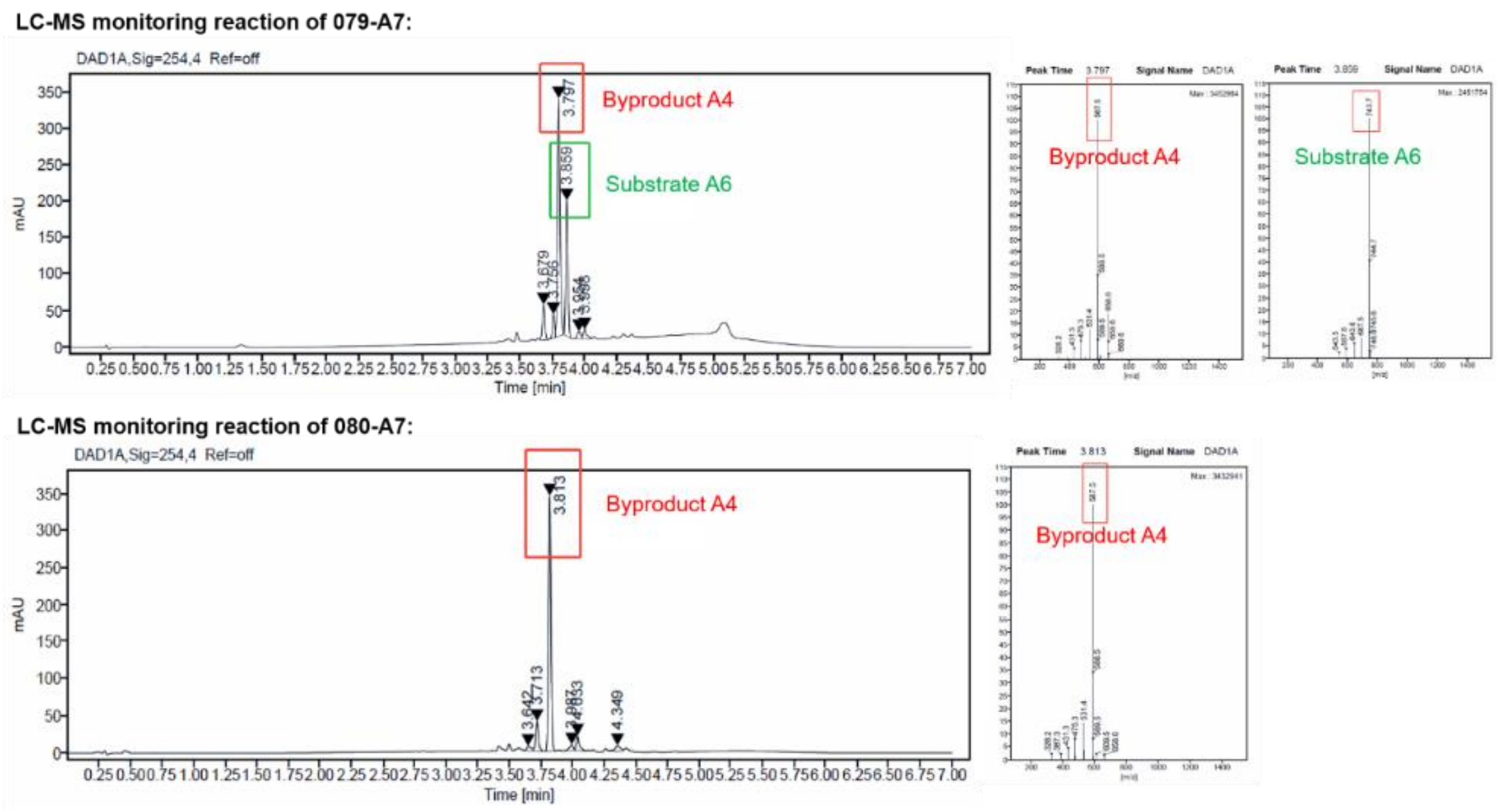

Figure R1. LC-MS monitoring click reaction of 079-A7 and 080-A7.

The click reaction was not

Table R1 Screening and Evaluation of Click Reaction Conditions

Cond.	NaVC (eq.)	$CuSO_4 \cdot 5H_2O$ (eq.)	Reaciton Solvent	Tem.	Time	Results
A	1	0.5	THF/H_2O(1:1)	rt	2h	A6 was completely consumed; byproduct A4 appeared as the main peak, and target product A7 accounted for less than 1%.
B	1	0.5	$tBuOH/H_2O$(1:1)	rt	2h	A6 was fully consumed, with byproduct A4 as the dominant peak and target product A7 at approximately 10%. The product peak disappeared after standing overnight.
C	1	0.5	DCM/H_2O(1:1)	rt	2h	
D	2	0.5	THF/H_2O(1:1)	rt	2h	
E	0.6	0.3	THF/H_2O(1:1)	rt	2h	

F	1	0.5	THF/H_2O(1:1)	0°C	2h	A6 was fully consumed, byproduct A4 was the main peak, and target product A7 was less than 1%.
G	1	0.5 containing THPTA	THF/H_2O(1:1)	rt	2h	A6 was completely consumed, byproduct A4 was the main peak, and target product A7 was less than 1%.

(2) So, we have alternatively synthesised monovalent AVC-Z1H with shorter PEG linker (AVC-Z1H-S, Supplementary Scheme 7). AVC-Z1H-S possesses a similar DFR value with AVC-Z1H, but its antiviral activity is significantly reduced, with median IC_{50}s of 31.79 nM against CA07 and 12.84 nM against PR8. In comparison, AVC-Z1H showed potent antiviral activity, with median IC_{50}s of 20.90 nM against CA07 and 1.03 nM against PR8. The reduced activity was presumably due to the absence of virus cross-linking capacity. These results have been been incorporated into the manuscript (line 156-161 on page 6-7). The scheme of AVC-Z1H has been supplemented as Supplementary Scheme 7 and the antiviral activity of AVC-Z1H-S has been supplemented into Supplementary Table 1.

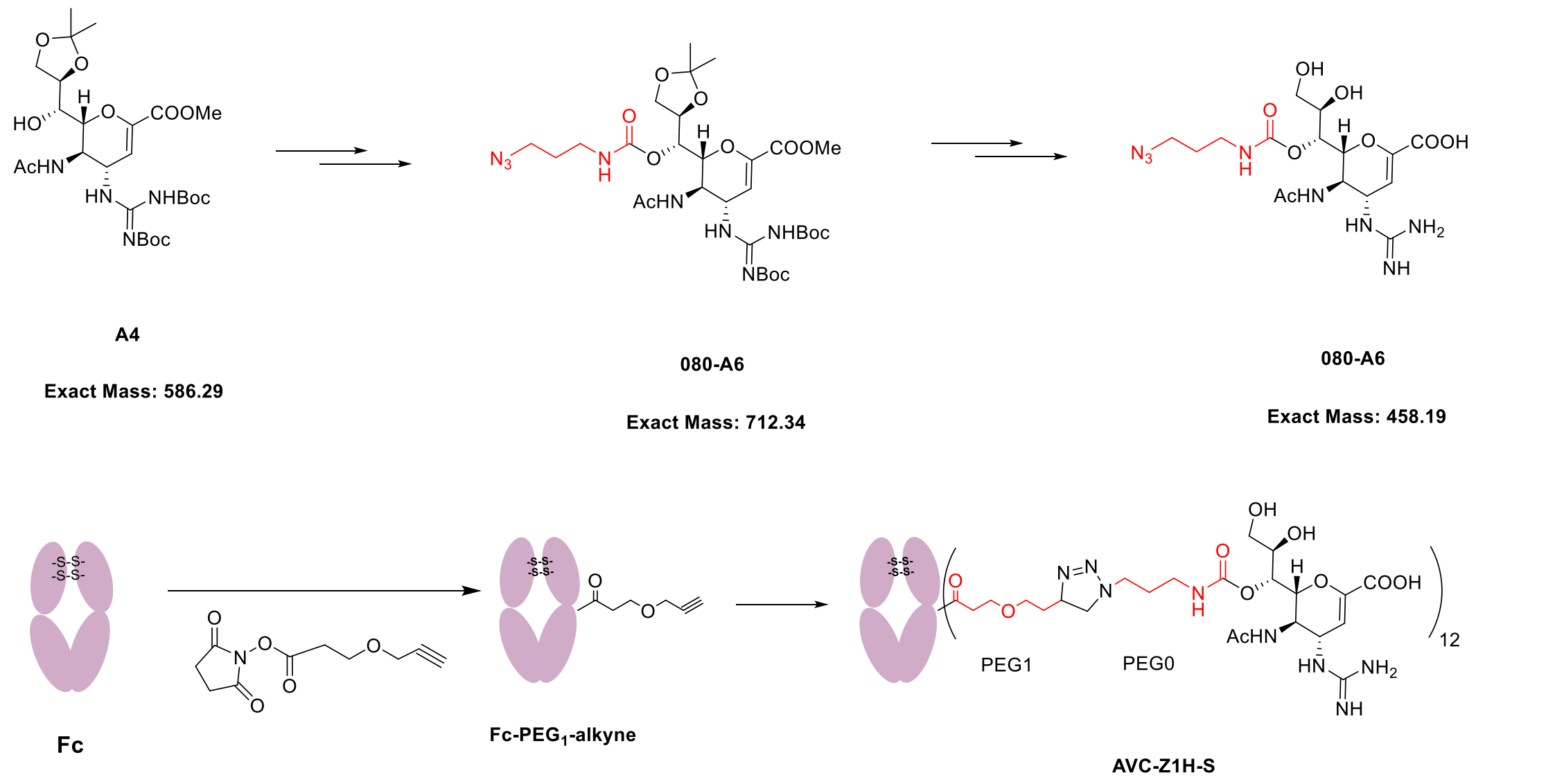

Supplementary Scheme 7. Synthesis of negative control AVC-Z1-S. The reactions and conditions are as the same as AVC-Z1.

Table R2 Antiviral activity of AVC-Z1H and AVC-Z1H-S

Compounds	PEG linkers	EC_{50} (nM)	
		A/Puerto Rico/8/1934 (H1N1)	A/California /07/2009(H1N1)
AVC-Z1H	PEG4+PEG3	1.63±0.02	20.90±2.65
AVC-Z1H-S	PEG1+PEG0	12.84±1.91	31.79±2.68

Point 2. Inclusion of a short-linker control group to determine the root cause of entry blockade. In the entry blockade assays (Figure 3e), a short-linker control group incapable of inducing cross-linking should be included. By comparing the inhibitory effects of AVC-Z3 versus this short-linker counterpart during the viral entry process, the authors can exclude the possibility that the inhibition is simply due to the bulky Fc-conjugate sterically shielding adjacent hemagglutinin (HA) binding sites. This would

prove that physical viral aggregation is indeed the primary driver of impaired viral entry.

Response: Thank you for your professional advice. Due to the synthetic difficulties encountered with the above short-chain trivalent conjugate controls, (1) we conducted the entry blockade assays using the short-linker AVC-Z1H-S. The results showed that AVC-Z1H-S also containing the bulky Fc groups did not block the viral entry process (Supplementary Fig. 7), indicating that the inhibition is not simply due to the bulky Fc-conjugate sterically shielding adjacent hemagglutinin (HA) binding sites. The inter-viral cross-linking might need a sufficiently long linker. These results have been been supplemented as Supplementary Fig. 7 and the description have been incorporated into the manuscript (line 221-227 on page 9-10).

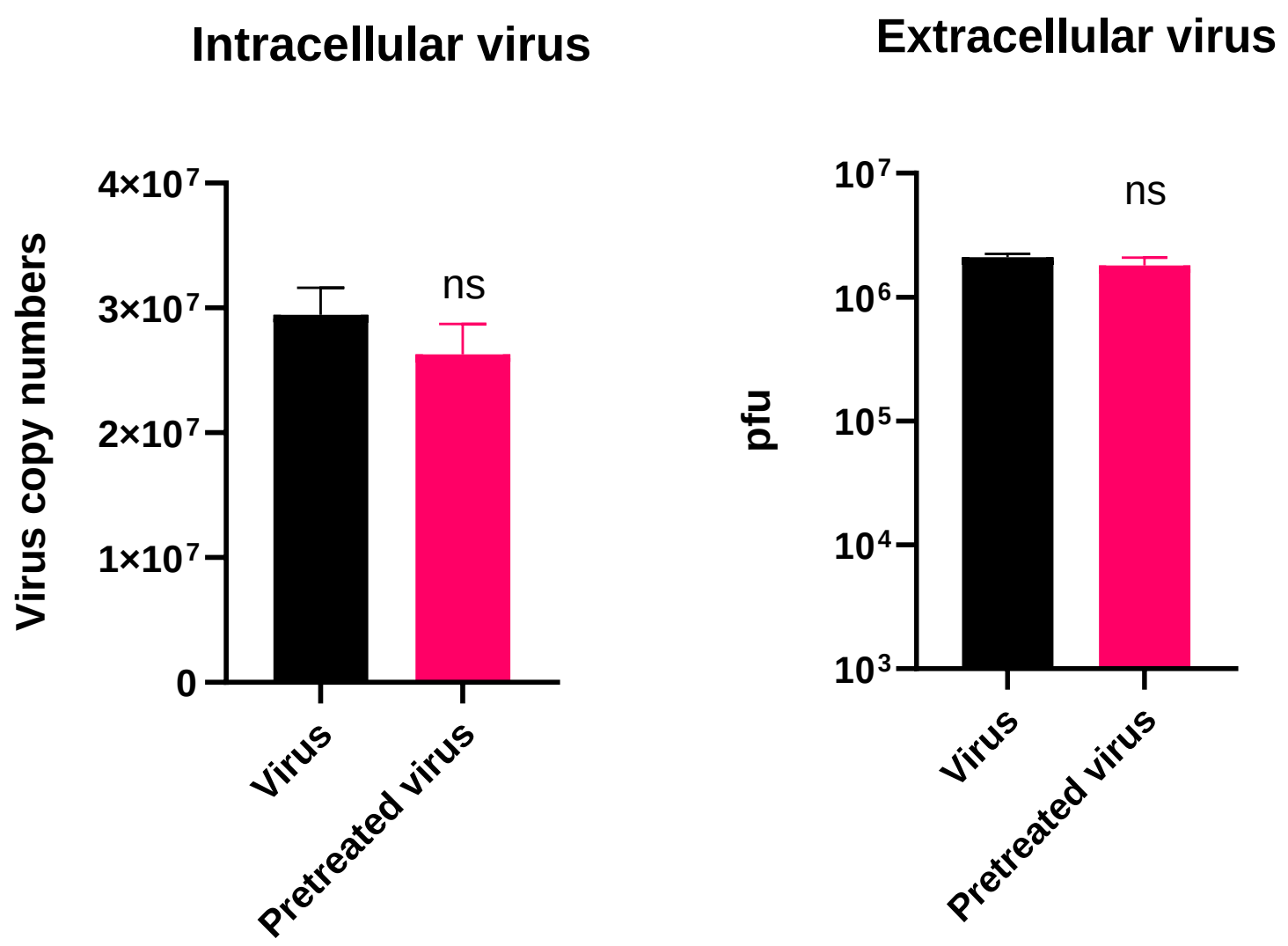

Supplementary Fig. 7. The entry blockade assays of AVC-Z1H-S with short linker. Then negative control AVC-Z1H-S also containing the bulky Fc groups did not block the viral entry process. Data are representative of

two independent experiments. data are shown as mean ± s.d.

(2) Furthermore, we validate the inter-viral cross-linking using trivalent small molecules (Z3). The particle size of the multivalent zanamivir–NA complexes increased, correlated positively with zanamivir valence. The results suggested that multivalent small molecules (eg. Z3) could also cross-link NA protein (Figure R3). Z3 produced significantly larger NA aggregates (~30 nm) than Z2 and Z1 (10~20 nm).

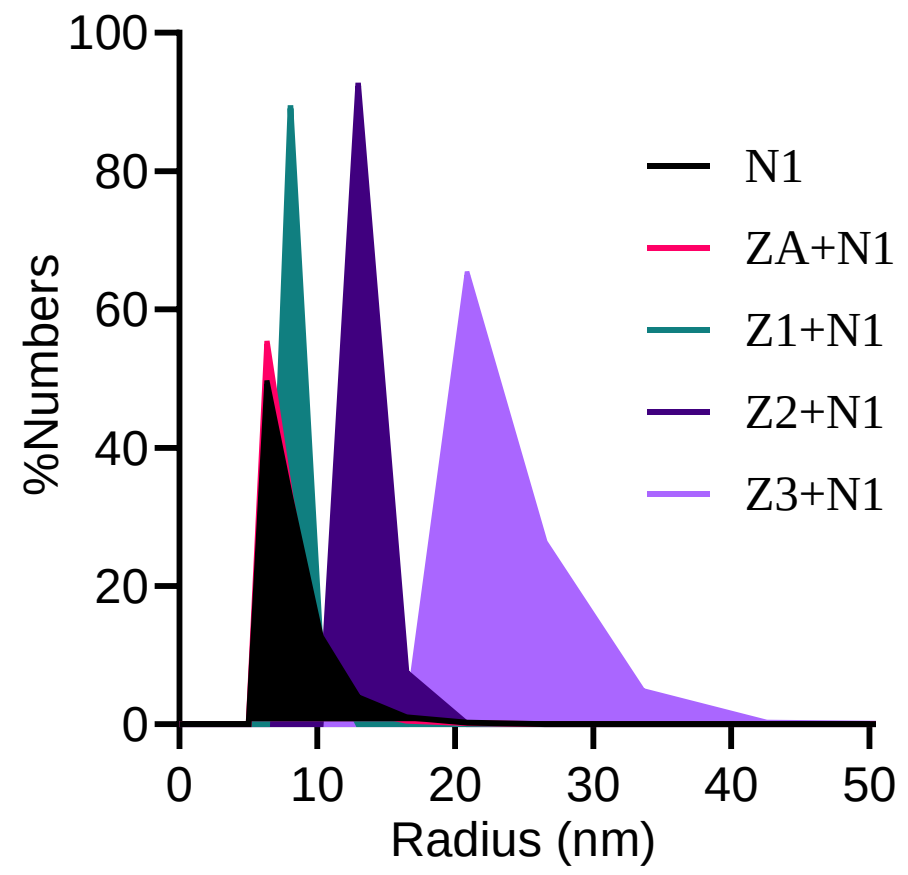

Figure R3. Particle size distribution patterns of N1 and multivalent zanamivir complex. Zanamivir + N1, Z1+N1, Z2 + N1 and Z3+N1 revealed that the multivalent zanamivir could also cross-link NA protein.

Point 3. To substantiate the central claim that an optimal linker length triggers effective cross-linking, it is essential for the authors to provide a comprehensive table in the Supporting Information. This table must explicitly detail the physical parameters for each experimental group, including the specific number of PEG units, the exact conjugation sites,

and the calculated extended lengths. Such detailed information will help readers better understand the relationship between linker properties and cross-linking efficiency, thereby strengthening the mechanistic rigor of the study.

Response: Thank you for your professional advice. According to your comments, we have provided a comprehensive table in the Supporting Information, including specific number of PEG units, the exact conjugation sites, and the calculated extended lengths. This comprehensive table have been been supplemented as the Supplementary Table 2. And the detailed discussions about the linker difference between trivalent platform and CD388 have also supplemented in manuscript (line 469-475 on page 21).

Supplementary Table 2 Comprehensive information of different AVCs

Comp.	Conjugation sites	No. of atoms (between NAI monomers)	Distance (between NAI monomers)	No. of atoms (between NAI and Fc)	Distance (between NAI and Fc)
AVC-Z1H	Lysine residue of Fc	/	/	33	～36Å
AVC-Z1H-S	Lysine residue of Fc	/	/	15	～18Å
AVC-Z3	Lysine residue of Fc	39	～37Å	33	～36Å
AVC-P3	Lysine residue of Fc	39	～37Å	33	～36Å
CD388	Lysine residue of Fc	15	～18Å	32	35～40 Å

Point 4. Refinement of the Mechanistic Logic in the Introduction. The authors note in the Abstract and Background that while CD388 has validated the clinical potential of Fc-conjugation, its underlying pharmacodynamic mechanism remains elusive. However, the manuscript currently lacks a clear discussion regarding the structural and mechanistic distinctions or connections between the proposed trivalent platform and CD388. This section should be expanded to better contextualize the current work, highlighting how the trivalent design and cross-linking mechanism address the limitations of CD388 and advance the field of Fc-conjugated antiviral drugs.

Response: Thank you for your professional advice. For a more detailed comparison between trivalent AVC-P3 and CD388, the key distinctions or connections are as follows: In structural design, AVC-P3 select the neuraminidase (NA) inhibitor peramivir and adopts a trivalent design to conjugate a total of 11 peramivir molecular, facilitating multivalent cross-linking with viruses. In compression, CD388 select the NA inhibitor zanamivir and adopts a bivalent design to conjugate a total of 9 zanamivir molecular. Furthermore, trivalent platform adopts a longer and more flexible linker design compared with CD388. The flexible 39-atom central-linker of trivalent platform that separates the peramivir monomers by ~39 Å. However, the distance between zanamivir monomers of CD388 as ~18 Å. In mechanism, the additional conjugated NAIs and the extended

linker of trivalent platform facilitated cross-linking with viruses, thereby coupling blockade of viral entry with restriction of progeny release. This cross-linking–mediated dual-stage inhibition represents a mechanistic shift from enzymatic suppression to physical viral immobilization. These discussions have been been incorporated into the manuscript (line 464-479 on page 20-21).
